# Synthesis of 3-selanylbenzo[*b*]furans promoted by SelectFluor®†

**DOI:** 10.1039/d0ra01907k

**Published:** 2020-04-07

**Authors:** Maurício Carpe Diem Ferreira Xavier, Eduardo Martarelo Andia Sandagorda, José Sebastião Santos Neto, Ricardo Frederico Schumacher, Márcio Santos Silva

**Affiliations:** Laboratório de Síntese Orgânica Limpa – LASOL, Centro de Ciências Químicas, Farmacêuticas e de Alimentos – CCQFA, Universidade Federal de Pelotas – UFPel Capão do Leão RS Brazil silva.ms@ufpel.edu.br ricardo.schumacher@ufsm.edu.br; Departamento de Química, Universidade Federal de Santa Maria – UFSM Santa Maria RS Brazil; Departamento de Química, Universidade Federal de Santa Catarina – UFSC Florianópolis SC Brazil

## Abstract

A simple and practical protocol for the synthesis of 3-selanyl-benzo[*b*]furans mediated by the SelectFluor® reagent was developed. This novel methodology provided a greener alternative to generate 3-substituted-benzo[*b*]furans *via* a metal-free procedure under mild conditions. The intramolecular cyclization reaction was carried out employing an electrophilic selenium species generated *in situ* through the reaction between SelectFluor® and organic diselenides. The formation of this electrophilic selenium species (RSe-F) was confirmed by heteronuclear NMR spectroscopy, and its reactivity was explored.

## Abstract 2

This novel methodology provided a greener alternative to generate 3-substituted-benzo[*b*]furans mediate by Selectfluor® reagent. The formation of this electrophilic selenium species (RSe-F) was confirmed by heteronuclear NMR spectroscopy.

## Graphical abstract

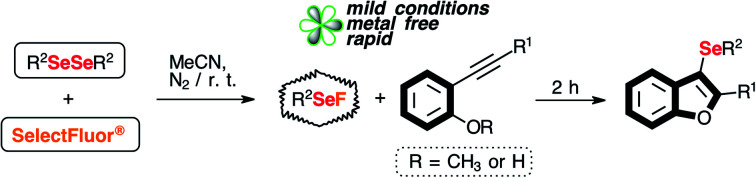

The benzo[*b*]furan scaffold is an important structural motif that is present in natural products and in synthetic compounds with therapeutic proprieties.^1^ Substituted benzo[*b*]furans have shown a broad range of biological activities,^2^ being found in a variety of pharmaceutical targets, such as Viibryd® and Ancoron® (Fig. 1).^3^ These drugs are used for treatment of depression and for cardiac arrhythmias, respectively. An efficient method to obtain substituted benzo[*b*]furans is the intramolecular cyclization reaction between 2-alkynylphenol or 2-alkynylanisole derivatives with different electrophilic species to generate a wide variety of 3-substituted-benzo[*b*]furans. This strategy is especially useful because of the atom-economic synthesis under mild conditions.^4^

**Fig. 1:**
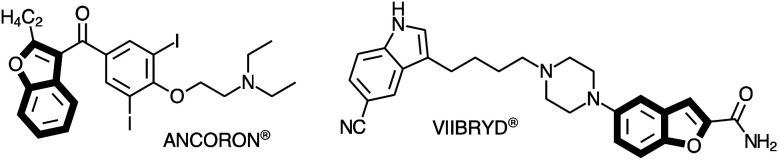
Substituted benzo[*b*]furans in commercial drugs.

Organoselenium compounds have attracted great interest due the large number of biological applications and their versatile reactivity.^5^ From a synthetic point of view, the ease cleavage of the Se–Se bond in diselenide compounds can generate species with different reactivity, as radical, electrophile, and nucleophile. This ample usefulness becomes the diselenides in key synthetic intermediates to introduce selenium moiety in organic compounds or to catalyse organic transformations.^5,6^

Despite the recent advances in the synthesis of 3-selanyl-benzo[*b*]furans, new electrophiles and reactional conditions were explored (Scheme 1).^7–9,11–15^ Initially, the establishing work by Larock and co-workers toward the synthesis of 3-selanyl-benzo[*b*]furans through the intramolecular cyclization of 2-(phenylethynyl)anisole with PhSeCl in CH_2_Cl_2_ at room temperature.^7^ In 2009, Zeni and co-workers demonstrated the synthesis of 3-selanyl-benzo[*b*]furans employing PhSeBr as an active electrophile.^8^ A pioneering protocol was reported by Zeni and co-workers, which employed FeCl_3_ (1.0 equiv.) and diorganyl diselenides in CH_2_Cl_2_ at 45 °C.^9^ Additionally, Lewis acids have been used as effective catalysts in Se–Se bonding cleavage to access functionalized selenium compounds.^10^ Afterward, alternative methods were developed, such as the synthesis of 3-selanyl-benzo[*b*]furans mediated by PdCl_2_/I_2_, I_2_/water, and CuI (1.5 equiv.).^11–14^ More recently, Liu and co-workers reported a radical cyclization reaction using selenium powder as selenium source and AgNO_3_ as catalyst in DMSO at 100 °C.^15^

**Scheme 1:**
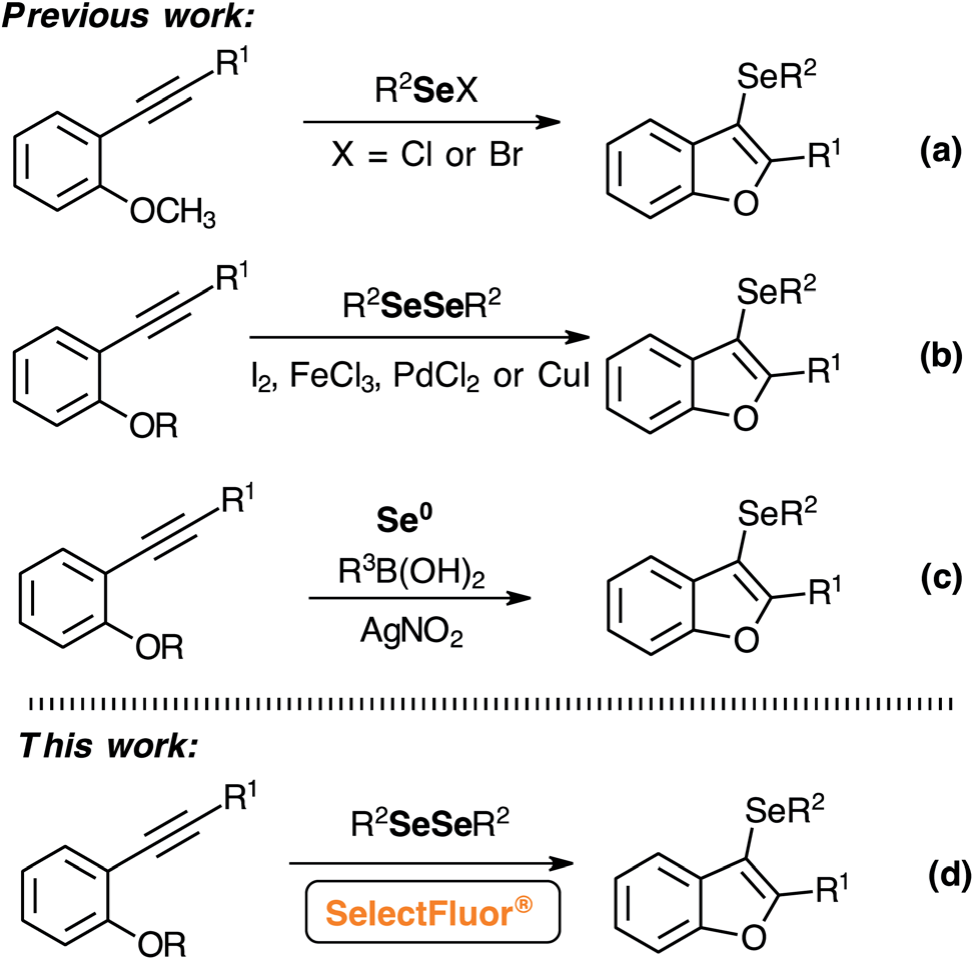
Methodologies to prepare 3-selanyl-benzo[*b*]furans.

Although, there are different methodologies to prepare 3-selanyl-benzo[*b*]furans and other functionalized selenium compounds through the reaction between diselenides compounds with oxidant reagents or Lewis acids, alternative electrophilic selenium species should be employed to avoid metals and/or toxic reagents.^9–15^ Furthermore, RSeCl and RSeBr,^7,8^ obtained from the reaction of diselenides with SO_2_Cl_2_ (or Cl_2_) and Br_2_ respectively, are commercially available and largely used as selenylating agent. However, these species present a low stability under moisture, and the high nucleophilicity of chloride and bromide leaving groups can lead to undesirable side reactions.

On the other hand, SelectFluor® is a versatile reagent used for different applications, such as fluorination reactions,^16^ C–H functionalization^17^ and organic function transfer.^18^ In addition, SelectFluor® has been used as an efficient method for intramolecular annulation reactions, due its higher reactivity.^19^ This ample application together with the desirable characteristics of the SelectFluor®, such as the higher stability, non-hydroscopic solid and hazard-free source of fluorine,^20^ promoted new possibilities to investigate fluorine chemistry. In 2004, Poleschner and Seppelt prepared PhSeF derivatives by the reaction between diorganyl diselenides and XeF_2_ in CH_2_Cl_2_ as a solvent at −40 °C.^21^ The products were characterized by low-temperature ^19^F and ^77^Se NMR, and it was the first confirmation of this type of electrophilic selenium compound. Although electrophilic selenium catalysis (ESC) with electrophilic fluoride reagents as oxidants has been demonstrated in the functionalization of alkenes,^22^ fewer knowledge about the reactivity of this selenium electrophilic species is available in the literature.^23^

Based on the development of new electrophilic selenium reagents,^9–14,24^ herein, we describe a metal-free synthesis of 3-selanyl-benzo[*b*]furans under mild conditions using this very reactive electrophilic selenium species (RSe-F), generated *in situ* at room temperature by the reaction of diorganyl diselenides with SelectFluor® reagent (Scheme 1). Moreover, the higher reactivity of RSe-F species could be explored for the insertion of selenium moiety in other building blocks because the environmentally friendly reactional condition, and the replacing chlorine and bromine by the non-nucleophilic fluorine counter ion, can partially circumvented some side reactions.

## Results and discussion

We commenced optimization of the reaction conditions using 2-phenylalkynylanisole 1a and diphenyl diselenide 2a as standard substrates. The reaction conditions were investigated as outlined in Table 1.

**Table 1:** Optimization of the reaction conditions for the synthesis of 3-phenylselanyl-benzo[*b*]furan 3a^a^

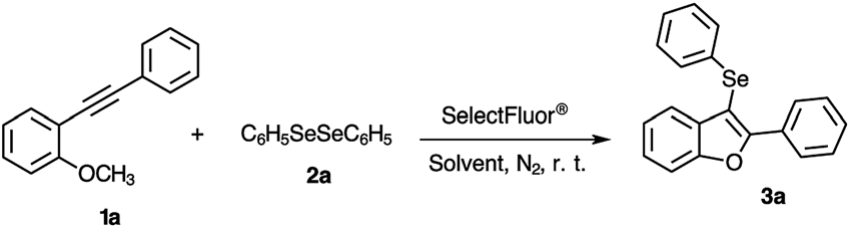					
#	2a (mmol)	F® (mmol)	Solvent (3.0 mL)	Time (h)	Yield^b^ (%)
1	0.125	0.250	MeCN	2	97
2^c^	0.125	0.250	MeCN	2	67
3^d^	0.125	0.250	MeCN	2	70
4	0.125	0.125	MeCN	2	90
5	0.150	0.125	MeCN	2	92
6	0.125	0.062	MeCN	2	40
7	0.125	0.125	DMSO	24	N.R.
8	0.125	0.250	DMF	24	79
9	0.125	0.250	THF	24	55
10	0.125	0.250	EtOH	24	61
11	0.125	0.250	PEG-400	24	57
12	0.125	0.250	Glycerine	24	45

a Reactions performed using 2-phenylalkynylanisole 1a (0.250 mmol) with diphenyl diselenide 2a and solvent under N_2_ atmosphere.

b Yields of isolated product.

c Reaction performed under air atmosphere.

d The reaction was performed using 1.0 mL of MeCN; N.R. = no reaction.

Initially, the reaction was carried out using 0.250 mmol of 1a, 0.125 mmol of 2a and 0.250 mmol of SelectFluor® in MeCN at room temperature under N_2_ atmosphere. After 2.0 h, the yield of product 3a was 90% (Table 1, entry 1). When the reaction was performed under air atmosphere, product 3a was obtained in just 67% yield (Table 1, entry 2 *vs.* 1). The reduction of the amount of MeCN solvent was not beneficial for the reaction (Table 1, entry 3 *vs.* 1). When the amount of SelectFluor® was decreased to 0.125 mmol, the reaction performance was similar to entry 1 (Table 1). When the reaction was carried out with a small excess of diphenyl diselenide 2a, no effective improvement in the reaction condition was observed (Table 1, entry 5 *vs.* 1). These outcomes clearly demonstrate that the dependence on the amount of diorganyl diselenide with SelectFluor® reagent is not stoichiometric. A solvent evaluation was performed (Table 1, entries 7–12), with DMF demonstrating satisfactory yield but a longer reaction time (Table 1, entry 8 *vs.* 1). With DMSO solvent the formation of product 3a was not detected by TLC and GC (Table 1, entry 7). With THF, EtOH, PEG-400 and glycerine solvents the yields and reaction times were unsatisfactory (Table 1, entries 9–11). It is noticeable that this synthetic protocol is sensitive to water content, since the reaction using wet MeCN resulted in yield decrease and the formation of seleninic acid was observed by ^77^Se-{^1^H} NMR.

**Scheme 2:**
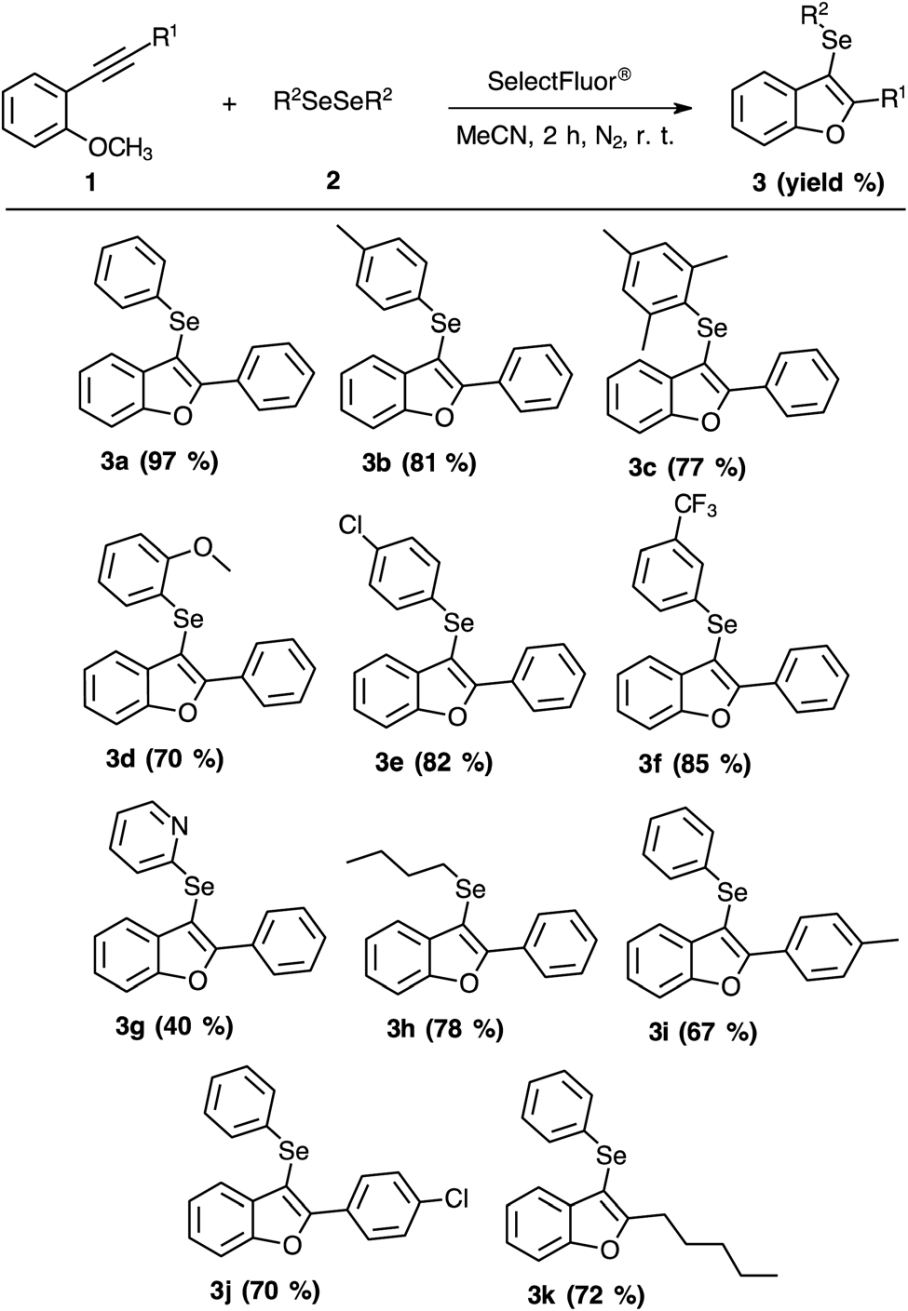
Substrate scope for the synthesis of 3-organylselanyl-benzo[*b*]furans 3a–k.

Next, we turned our attention to the reactional scope (Scheme 2), evaluating different 2-organylalkynylanisoles 1 with diverse diorganyl diselenides 2 under optimized reaction conditions (Table 1, entry 4).

**Scheme 3:**
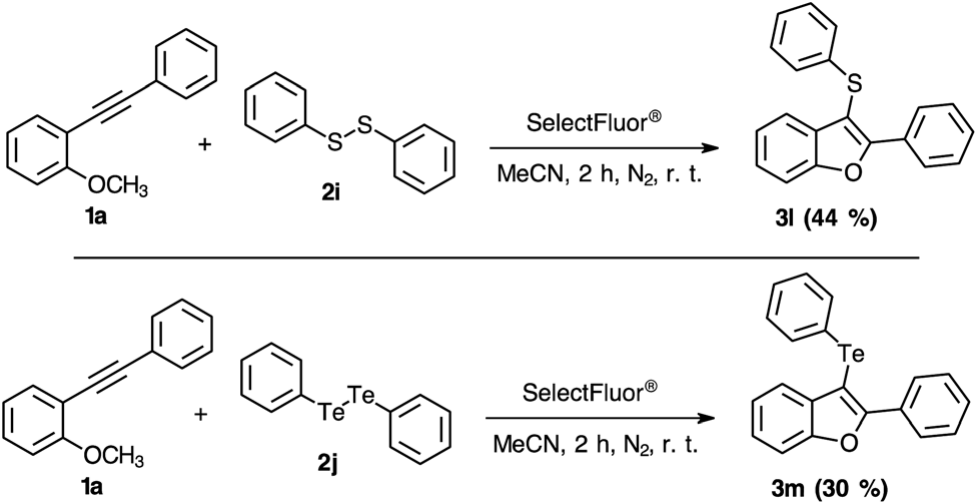
Chalcogenide scope for the synthesis of 3-chalcogenyl-benzo[*b*]furans 3l–m.

As summarized in Scheme 2, the substitution patterns on the phenyl moiety were satisfactory in all examples. The presence of methyl substituent, an electron-donating group, at *para*-position or *ortho* and *para*-positions afforded excellent yields (Scheme 2, 3b and 3c). The presence of methoxyl group at *ortho*-position also provided a satisfactory yield (Scheme 2, product 3d). When electron-withdrawing groups were evaluated, the conversion to the products 3e (*para*-chloride) and 3f (*meta*-CF_3_) yielded 82% and 85%, respectively (Scheme 2). When we examined the presence of pyridyl moiety on the aromatic diselenide, a moderate yield was obtained (Scheme 2, 3g). In an attempt to improve the performance of pyridyl moiety, a reaction to obtain the product 3g was carried out in 24 h at room temperature or employing heating (oil bath) of 50 °C for 2 h. However, both experimental changes were not affective to increase the yield of compound 3g. Furthermore, when the aromatic diselenides were switched for an aliphatic diselenide (Scheme 2, 3h) the performance of the reaction remained suitable.

Similarly, a substrate scope of the 2-organylalkynylanisoles 1 was also carried out (Scheme 2). These substrates were prepared by the Sonogashira coupling reaction between terminal alkynes with 2-bromoanisole.^7^ Once with the 2-organylalkynylanisoles 1 in hands, we started with evaluating the effect of electron-donating and electron-withdrawing groups. As can be seen in Scheme 2, *para*-methyl (3i) or *para*-chloride (3j) substituents gave a satisfactory efficiency, 67% and 70% of yield, respectively. When the aryl group was replaced by the alkyl group in the alkyne reagent the yield was similar (Scheme 2, 3k).

After determining the substrate scope regarding substituted diorganyl diselenides 2 and 2-organylalkynylanisoles 1, the reactivity of others diorganyl dichalcogenides (S and Te) were tested (Scheme 3). Notably, the reaction efficiency was reduced employing sulphur or tellurium elements. When diphenyl disulphide was used instead of diselenide, the yield has changed to 44% (Scheme 3, 3l). Considering the tellurium atom on the electrophilic intramolecular cyclization reaction, the yield was just 30% (Scheme 3, 3m). Based on the literature, it is possible to check the higher stability of the S–F bond,^25^ that could explain the lower effectivity of the 3-sulphuryl-benzo[*b*]furan 3l synthesis. However, there is not enough data describing the Te–F bonding stability and reactivity.^21,26^

**Scheme 4:**
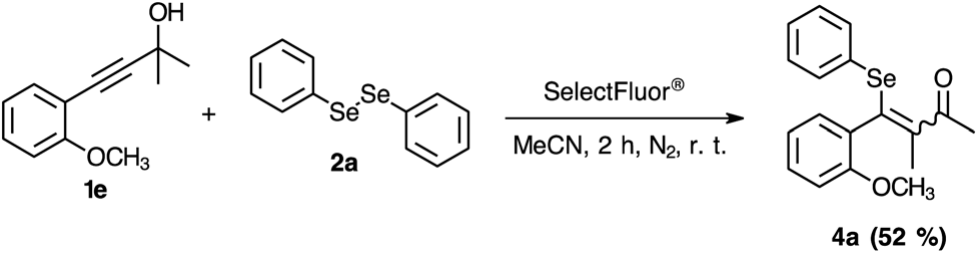
Reactivity of 2-propagylanisole 1e in the reaction with the electrophilic Se–F species.

**Fig. 2:**
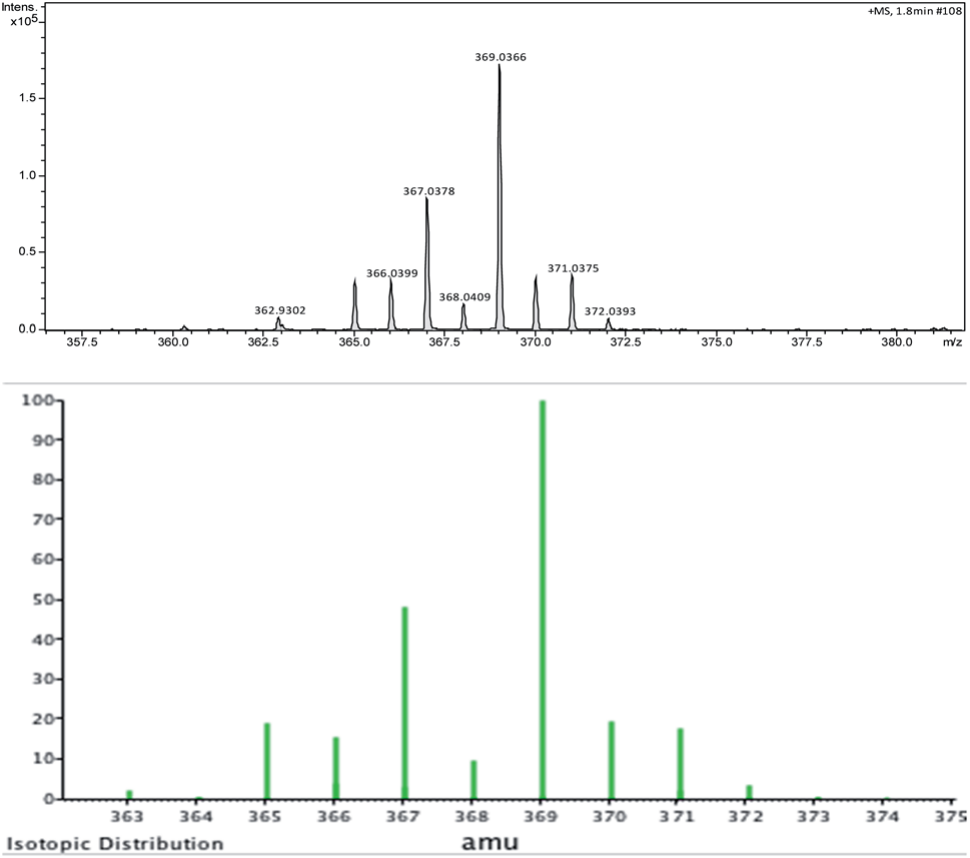
HRMS and isotopic distribution of product 4a (up: experimental analysis; and down: calculated MW + Na^+^ = 369.0369).

To further extend the practicability of this reaction, the reactivity of the 2-propagylanisole 1e was explored. Thus, the reaction was carried out using 0.250 mmol of 1e, 0.125 mmol of 2a and 0.250 mmol of SelectFluor® in MeCN at room temperature under N_2_ atmosphere. After 2.0 h, the yield was moderate (Scheme 4, 4a). To our surprise, the ^1^H and ^13^C NMR analyses have demonstrated altered standard spectra. These outcomes encouraged us to perform additional analyses to evaluate the product obtained. Consequently, HRMS (Fig. 2), infrared and NMR (ESI: ^77^Se-{^1^H}, COSY, HSQC and HMBC) analyses were carried out to check the structural assignment. All these findings support that a semi-pinacol rearrangement occurred, which an insertion of the C_6_H_5_Se group has occurred followed by a CH_3_ group shifting and providing the ketone 4a product (Scheme 4). Moreover, this reactivity of the 2-propagylanisole 1e is comparable with the literature, by isomerization^27^ or addition of others electrophilic species.^28^

**Scheme 5:**
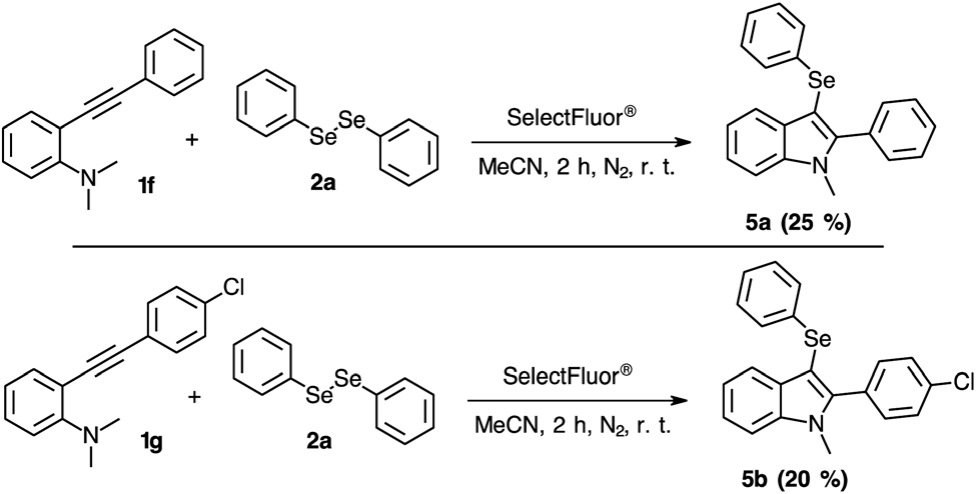
Substrate scope for the synthesis of 3-selanylindoles 5a–b.

On evaluating the 2-organylalkynylanilines 1f and 1g to obtain 3-selanylindoles 5a–b, the results were less successful than the 3-selanyl-benzo[*b*]furans 3 (Scheme 5). This lower effectivity was established by a complex mixture of products in the TLC and ^1^H NMR analyses, obtaining the indoles 5a and 5b in just 25% and 20% of yield, respectively. The possible by-products can be suggested by the higher reactivity of the indoles with fluorine reagents.^29^ Additionally, a complete structural elucidation of the product 5b was performed to undoubtedly confirm the product 5b and obtain information about ^15^N NMR chemical shift profile (see ESI†).

**Scheme 6:**
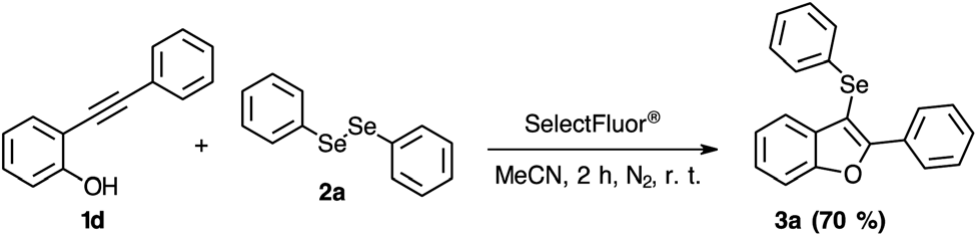
Synthesis of 3-phenylselanyl-benzo[*b*]furan 3a employing 2-phenylalkynylphenol 1d.

The reactivity of the 2-phenylalkynylphenol 1d was also evaluated. According to Scheme 6, it is possible to observe that the phenol organic functional group is not sensitive to this higher reactive selenium electrophilic species, which the product 3a was obtained in 70% of yield.

In order to gain insight into the mechanism, ^1^H, ^13^C-{^1^H}, ^19^F and ^77^Se-{^1^H} NMR analyses of a mixture between the diphenyl diselenide 2a and SelectFluor® were performed (ESI).^30^ For this purpose, 0.075 mmol of 2a and 0.150 mmol of SelectFluor® were solubilized in 1.0 mL of deuterated CD_3_CN and the NMR analyses were carried out at 25 °C. It was observed that a shielding has happened in the ^1^H and ^13^C-{^1^H} NMR chemical shifts of the SelectFluor® reagent, reflecting the leaving of fluorine atom. A deshielding was observed in the aromatic groups of the 2a compound around 1.0 ppm in the ^1^H NMR spectrum. Considering the ^19^F NMR experiment, the disappearance of ^19^F NMR chemical shift of the SelectFluor (ESI: 48.0 ppm) and the arising of a signal at −182.0 ppm, suggested the formation of a new fluorine compound. Finally, evaluating the ^77^Se-{^1^H} NMR analysis a new signal arisen at 1430.0 ppm, probably related to the Se–F bonding formation.^21,31^ When the ^19^F NMR experiment was performed employing 0.075 mmol of 2a with 0.075 mmol of SelectFluor®, similarly to the reaction conditions, a signal at −46.5 ppm was detected. But, the ^77^Se-{^1^H} NMR experiment did not show new peak, only regarding to the diphenyl diselenide compound.

**Scheme 7:**
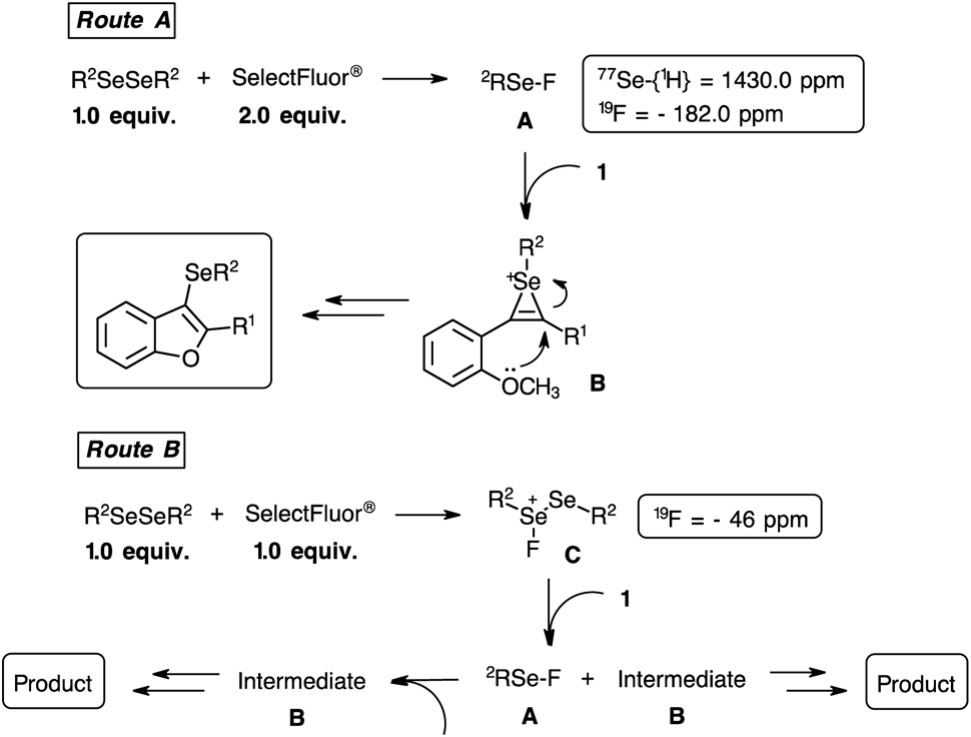
Plausible mechanism routes for the synthesis of 3-organylselanyl-benzo[*b*]furans 3.

Considering the NMR results^21,31^ and based on the literature,^7–9^ we have suggested a plausible mechanism for intramolecular cyclization reaction (Scheme 7). We have proposed two routes, based on the stoichiometric of the reagents, which both routes have the same intermediate detected by heteronuclear NMR spectroscopy. Initially, the formation of a higher reactive selenium electrophilic species A (^77^Se-{^1^H} = 1430.0 ppm and ^19^F = −182.0 ppm) is performed by the reaction between 1.0 equiv. of diselenide compound with 2.0 equiv. of SelectFluor® reagent (Scheme 7, route A). Next, the electrophilic selenium A reacts with the 2-organylalkynylanisole 1 to provides the intermediate B. Therefore, an intramolecular cyclization occurs by the oxygen attack on the activated triple bond and producing the 3-selanyl-benzo[*b*]furans. The CH_3_ or H leaving group of the methoxyl or phenol organic function, respectively, could be favoured by the nucleophilic attack of the nitrogen atom derived from the SelectFluor® residue. Considering the route B, a sub stoichiometric amount of SelectFluor® (1.0 equiv.) was employed. To explain the effectiveness of this experimental condition, the formation of an electrophilic selenium species C (^19^F = −46.5 ppm) was suggested, which the attack of 2-organylalkynylanisole 1 provides the intermediates A and B. Consequently, the intermediate B produces the desired product, and the intermediate A forms the species B, also resulting the 3-selanyl-benzo[*b*]furan compound.

Related to the cleavage of the Se–Se bonding for the formation of the species A, there are some studies that support an homolytic cleavage.^32^ Our tests to evaluate this type of cleavage have demonstrated a yield reduction. Under the standard reaction conditions, the reaction between 1a and 2a reagents was performed in the presence of 2,2,6,6-tetramethylpiperidin-1-oxyl (TEMPO) or benzene-1,4-diol, used as radical inhibitors (2.0 equiv.). The product yield in each test was 40% and 42%, respectively. Although, these findings support an homolytic cleavage of Se–Se, the higher reactivity of SelectFluor® against these radical inhibitors limit the conclusions of the reaction pathway. On the other hand, when we evaluate the mechanism involved in the ditelluride 2j, a different outcome was provided. At first, TEMPO as a radical inhibitor was added, and following our optimal experimental conditions, no product 3m was obtained. Considering the weak Te–Te bonding and the Te-oxidation facility,^33^ this result demonstrated that the reaction might occurred through a radical pathway.

## Conclusions

In summary, a simple and efficient protocol for the synthesis of 3-selanyl-benzo[*b*]furans was developed. The methodology provided a greener alternative to generate 3-substituted-benzo[*b*]furans *via* a metal-free procedure under mild conditions. Additionally, we have confirmed the formation of the Se–F bonding, and its reactivity as an electrophilic selenium species was assessed. Compared with traditional methods, this methodology is a mild, metal-free and simple tool for the generation of selenium electrophile. These results demonstrate new possibilities of reaction application, since little information can be found in the literature about the reactivity of RSe-F electrophiles.

## Experimental section

All commercial reagents and solvents were used without additional purification. TLC was performed on silica gel plates (Merck silica gel 60, F_254_), and the spots were visualized with UV light (254 and 365 nm) or by charring the plate dipped in vanillin solution. For the FTIR (Fourier Transform Infrared) in the attenuated total reflection mode (FTIR-ATR), the samples were submitted to KI and placed on the crystal surface of a FTIR Bruker Alpha-P spectrometer, obtained at the range of 4000–1500 cm^−1^. ^1^H, ^13^C, ^19^F, ^77^Se-{^1^H}, COSY, HSQC and HMBC NMR spectra were recorded using an NMR spectrometer with 400 MHz (Bruker, Avance III HD model). The probe was a 5 mm direct F-BBO (fluoride broadband observed). Spectra were recorded in deuterated chloroform at 298 K (25 °C). The reported data include chemical shift (*δ*), multiplicity, coupling constant (*J*) in hertz, and integrated intensity. The following abbreviations were used to explain multiplicities: s = singlet, d = doublet, dd = doublet of doublet, dt = doublet of triplet, t = triplet, td = triplet of doublet, q = quartet, quint = quintet, sex = sextet and m = multiplet. The ^19^F NMR chemical shifts are reported in ppm relative to PhCF_3_ (*δ* −63 ppm). The ^77^Se-{^1^H} NMR chemical shifts are reported in ppm relative to the internal standard C_6_H_5_SeSeC_6_H_5_ (*δ* 463 ppm). The NMR pulse sequence employed for ^77^Se-{^1^H} NMR experiments was gated decoupling. HRMS (*m*/*z*) were measured by ESI technique.

### General protocol for the preparation of 2-alkynylanisoles 1*via* Sonogashira coupling reaction^5^

To a two-necked round bottom flask containing PdCl_2_(PPh_3_)_2_ (1 mol%) and Et_3_N (3.0 mL) was added 2-bromoanisole (1.0 mmol) and terminal alkyne (1.5 mmol). The resulting solution was stirred for 5 minutes at room temperature. After this time, it was added CuI (2 mol%) and the reaction mixture was allowed to stir at 75 °C for 12 hours. After, the mixture was diluted with ethyl acetate (20.0 mL) and washed with saturated brine (2 × 20.0 mL). The organic phase was separated, dried over MgSO_4_ and concentrated under vacuum. The residue was purified by flash chromatography and eluted with hexane.

### General protocol for the preparation of 3-selanylalkynylanisoles 3

The corresponding diorganyl diselenides (0.125 mmol) was added to a round bottom flask flowed by addition of dry CH_3_CN solvent (2.0 mL). To this solution, SelectFluor® (0.250 mmol) was added under N_2_ atmosphere. As the SelectFluor® was dissolved the reaction colour changed from yellow to red-brown. After 5 minutes from the addition of SelectFluor®, a solution of 2-alkynylanisole 1 in dry CH_3_CN solvent (0.250 mmol in 1.0 mL) was added to the reaction mixture. The reaction colour usually changed from red-brown to a clear brown. The reaction progress was monitored by TLC. After reaction completion ethyl acetate (20.0 mL) and distilled water (20.0 mL) were added and the aqueous layer was washed with ethyl acetate (2 × 20.0 mL). Then, the combined organic layers were washed with distilled water (10.0 mL) to remove any remaining organic solvent. After removal of the solvent, column chromatography was performed using silica gel and either hexane or a mixture of hexane and ethyl acetate depending on the polarity of the product 3. The characterization data of synthesized products 3 are described in the ESI.†

#### 2-Phenyl-3-(phenylselanyl)benzo[*b*]furan 3a

Yield: 0.084 g (97%). White solid, mp = 40–41 °C. ^1^H NMR (CDCl_3_, TMS, 400 MHz) *δ* (ppm) = 8.12 (d, *J* = 7.2 Hz, 2H), 7.47–7.42 (m, 2H), 7.37–7.19 (m, 6H), 7.14 (dt, *J* = 7.4 and 1.0 Hz, 1H), 7.09–7.02 (m, 3H). ^13^C NMR (100 MHz, CDCl_3_) *δ* (ppm) = 157.2, 154.1, 131.8, 131.3, 130.1, 129.3, 129.2, 129.1, 128.4, 127.7, 126.2, 125.2, 123.4, 121.1, 111.1, 99.6. MS: *m*/*z* (rel intensity) 350 (M^+^ 32.4); 270 (100.0), 255 (7.8), 241 (17.0), 165 (28.5), 134 (8.4), 115 (4.5), 77 (5.4).

#### 2-Phenyl-3-[(4-methylphenyl)selanyl]benzo[*b*]furan 3b

Yield: 0.073 g (81%). Yellow solid, mp = 77 °C.^2^^1^H NMR (400 MHz, CDCl_3_) *δ* (ppm) = 8.13 (d, *J* = 7.2 Hz, 2H), 7.44 (t, *J* = 8.2 Hz, 2H), 7.38–7.34 (m, 2H), 7.33–7.24 (m, 1H), 7.22 (td, *J* = 7.7 Hz and 1.4 Hz, 1H), 7.15–7.11 (m, 3H), 6.88 (d, *J* = 8.0 Hz, 2H), 2.14 (s, 3H). ^13^C NMR (100 MHz, CDCl_3_) *δ* (ppm) = 156.9, 154.0, 136.1, 131.9, 130.1, 130.0, 129.5, 129.1, 128.4, 127.7, 127.4, 125.1, 123.3, 121.2, 111.1, 100.1, 20.9.

#### 2-Phenyl-3-(mesitylselanyl)benzo[*b*]furan 3c

Yield: 0.072 g (77%). Yellow solid, mp = 145–148 °C. ^1^H NMR (400 MHz, CDCl_3_) *δ* (ppm) = 8.04 (d, *J* = 7.3 Hz, 2H), 7.40–7.34 (m, 3H), 7.29 (m, 1H), 7.10 (dt, *J* = 1.4 and 8.0 Hz, 1H), 6.93 (dt, *J* = 7.5 and 1.0 Hz, 1H), 6.85 (m, 1H), 6.76 (s, 2H), 2.30 (s, 6H), 2.13 (s, 3H). ^13^C NMR (100 MHz, CDCl_3_) *δ* (ppm) = 153.9, 153.4, 142.2, 138.1, 131.6, 130.6, 128.9, 128.6, 128.3, 127.4, 126.4, 124.6, 122.8, 120.6, 110.9, 102.3, 24.1, 20.8. MS: *m*/*z* (rel intensity) 392 (M^+^ 37.9); 311 (2.3); 281 (4.8); 194 (100.0); 165 (33.3); 139 (5.4); 119 (14.8); 91 (16.7); 77 (10.4); 44 (12.2). HRMS calculated for C_23_H_20_OSe 392.0675, found: 392.0676.

#### 3-(2-Methoxyphenylselanyl)-2-(phenyl)benzo[*b*]furan 3d

Yield: 0.063 g (70%). Yellow oil. ^1^H NMR (400 MHz, CDCl_3_) *δ* (ppm) = 8.19 (d, *J* = 7.8 Hz, 2H), 7.57–7.52 (m, 2H), 7.43–7.31 (m, 4H), 7.24–7.21 (m, 1H), 7.10 (t, *J* = 7.7 Hz, 1H), 6.84 (d, *J* = 8.1 Hz, 1H), 6.78 (d, *J* = 7.7 Hz), 6.66 (t, *J* = 7.5 Hz, 1H), 3.93 (s, 3H). ^13^C NMR (100 MHz, CDCl_3_) *δ* (ppm) = 158.0, 156.4, 154.2, 132.1, 130.1, 129.2, 128.4, 128.1, 127.8, 126.8, 125.2, 123.4, 121.8, 121.3, 120.6, 111.1, 110.2, 97.8, 55.8. EM: *m*/*z* (rel intensity) 380 (M^+^ 85.4), 300 (100.0), 268 (13.8), 257 (17.0), 207 (14.5), 194 (35.7), 165 (64.5), 91 (14.4), 77 (25.6), 63 (12.2). HRMS calculated for C_21_H_16_O_2_Se + Na = 403.02125, found: 403.0220.

#### 3-[(4-Chlorophenyl)selanyl]-2-phenylbenzo[*b*]furan 3e

Yield: 0.078 g (82%). Yellow solid, mp = 87 °C. ^1^H NMR (400 MHz, CDCl_3_) *δ* (ppm) = 8.08 (d, *J* = 7.0 Hz, 2H), 7.46 (d, *J* = 8.2 Hz, 1H), 7.40–7.27 (m, 4H), 7.24 (td, 7.7 Hz, 1.4 Hz, 1H), 7.16–7.09 (m, 3H), 7.03–7.00 (m, 2H). ^13^C NMR (100 MHz, CDCl_3_) *δ* (ppm) = 157.3, 154.0, 132.3, 131.5, 130.4, 129.9, 129.5, 129.4, 129.3, 128.4, 127.7, 125.3, 123.5, 120.9, 111.2, 99.3. MS: *m*/*z* (rel intensity) 384 (M^+^ 37.2); 304 (100.0), 268 (22.1), 241 (18.6), 165 (38.8), 134 (13.5), 63 (3.7).

#### 2-Phenyl-3-[(3-trifluormethylphenyl)selanyl]benzo[*b*]furan 3f

Yield: 0.088 g (85%). Yellow solid, mp = 80 °C. ^1^H NMR (400 MHz, CDCl_3_) *δ* (ppm) = 8.17 (d, *J* = 7.3 Hz, 2H), 7.61–7.49 (m, 2H), 7.49–7.32 (m, 7H), 7.26–7.18 (m, 2H). ^13^C NMR (100 MHz, CDCl_3_) *δ* (ppm) = 157.7, 154.2, 132.7, 132.0, 131.5 (q, *J* = 34.4 Hz), 131.4, 129.8, 129.6, 129.5, 128.5, 127.8, 125.6 (q, *J* = 3.6 Hz), 125.5, 123.0 (q, *J* = 272.9), 123.6, 122.9 (q, *J* = 3.6 Hz), 120.9, 111.3, 98.7. MS: *m*/*z* (rel intensity) 418 (M^+^ 35.6), 338 (100.0), 309 (7.3), 268 (4.3), 241 (6.7), 165 (29.8), 139 (6.1), 115 (3.9).

#### 2-Phenyl-3-(2-pyridylselanyl)benzo[*b*]furan 3g

Yield: 0.024 g (40%). Yellow solid, mp = 46 °C. ^1^H NMR (400 MHz, CDCl_3_) *δ* (ppm) = 8.45 (ddd, *J* = 4.8, 1.8, 0.8 Hz, 1H), 8.23–8.20 (m, 2H); 7.60–7.55 (m, 2H), 7.47–7.35 (m, 4H), 7.32–7.29 (m, 2H), 7.01 (ddd, *J* = 7.4, 4.9, 1 Hz, 1H), 6.9 (dt, *J* = 8.0, 1.0 Hz). ^13^C NMR (100 MHz, CDCl_3_) *δ* (ppm) = 157.6, 157.4, 154.2, 150.0, 136.9, 131.7, 129.9, 129.5, 128.5, 127.8, 125.4, 123.6, 122.9, 121.1, 120.5, 111.3, 99.0. HRMS calculated for C_18_H_13_NOSe + Na = 374.0059, found: 374.0055.

#### 3-(Butylselanyl)-2-phenylbenzo[*b*]furan 3h

Yield: 0.066 g (78%). Yellow oil. ^1^H NMR (400 MHz, DMSO-*d*_6_) *δ* (ppm) = 8.19 (d, *J* = 7.8 Hz, 2H), 7.64 (d, *J* = 7.8 Hz, 2H), 7.52–7.55 (m, 2H), 7.47–7.43 (m, 1H), 7.40–7.32 (m, 1H), 2.80 (t, *J* = 7.2 Hz, 2H), 1.43 (quint, *J* = 7.1 Hz, 2H), 1.24 (sext, *J* = 7.3 Hz, 2H), 0.68 (t, *J* = 7.3 Hz, 2H). ^13^C NMR (100 MHz, DMSO-*d*_6_) *δ* (ppm) = 155.33, 153.26, 131.88, 129.76, 129.29, 128.65, 127.30, 125.44, 123.58, 120.73, 111.31, 99.90, 31.76, 27.65, 21.91, 13.22. EM: *m*/*z* (rel intensity) 330 (M^+^ 35.3), 274 (11.7), 245 (12.5), 194 (100.0), 165 (31.8), 41 (5.9).

#### 3-(Phenylselanyl)-2-(4-methylphenyl)benzo[*b*]furan 3i

Yield: 0.060 g (67%). White solid, mp = 62 °C. ^1^H NMR (400 MHz, CDCl_3_) *δ* (ppm) = 8.10 (d, *J* = 8.2 Hz, 2H), 7.55–7.49 (m, 2H), 7.34–7.20 (m, 6H), 7.18–7.10 (m, 3H), 2.39 (s, 3H). ^13^C NMR (100 MHz, CDCl_3_) *δ* (ppm) = 157.5, 154.0, 139.4, 131.9, 131.5, 129.0, 127.6, 127.3, 126.1, 124.9, 123.3, 121.0, 111.0, 98.8, 21.4. MS: *m*/*z* (rel intensity) 364 (31), 363 (4), 284 (100), 269 (11), 255 (9), 241 (13), 178 (33), 165 (4), 15 (1), 77 (22).

#### 2-(4-Chlorophenyl)-3-(phenylselanyl)benzo[*b*]furan 3j

Yield: 0.069 g (70%). Yellow solid, mp = 71–73 °C. ^1^H NMR (400 MHz, CDCl_3_) *δ* (ppm) = 8.07 (d, *J* = 8.7 Hz, 2H), 7.44–7.41 (m, 2H), 7.30 (d, *J* = 8.7 Hz, 2H), 7.23 (t, *J* = 7.7 Hz, 1H), 7.19–7.11 (m, 3H), 7.08–7.01 (m, 3H). ^13^C NMR (100 MHz, CDCl_3_) *δ* (ppm) = 155.9, 154.0, 135.2, 131.8, 131.1, 129.3, 129.2, 128.9, 128.7, 128.5, 126.4, 125.4, 123.5, 121.2, 111.2, 100.2. MS: *m*/*z* (rel intensity) 384 (M^+^ 51.34), 304 (100.0), 281 (7.35), 268 (25.64), 241 (19.30), 207 (17.43), 199 (16.27), 163 (25.01), 134 (7.98), 73 (10.12).

#### 2-(^*n*^Pentyl)-3-(phenylselanyl)benzo[*b*]furan 3k

Yield: 0.067 g (72%). Clear oil. ^1^H NMR (400 MHz, CDCl_3_) *δ* (ppm) = 7.47–7.42 (m, 2H), 7.28–7.22 (m, 4H), 7.18–7.10 (m, 3H), 2.97 (t, *J* = 7.5 Hz, 2H), 1.73 (p, *J* = 6.8 Hz, 1H), 1.32–1.29 (m, 4H), 0.84 (t, *J* = 6.5 Hz, 3H). ^13^C NMR (100 MHz, CDCl_3_) *δ* (ppm) = 163.8, 154.4, 131.8, 130.7, 129.13, 129.10, 126.0, 124.0, 123.0, 120.3, 110.9, 100.0, 31.3, 27.9, 27.3, 22.3, 13.9. EM: *m*/*z* (rel intensity) 344 (M^+^ 80.1), 287 (36.2), 264 (41.0), 207 (100.0), 178 (33.6), 131 (37.1), 115 (8.5), 102 (8.7), 77 (10.0). HRMS calculated for C_19_H_20_OSe + Na = 367.05764, found: 367.0549.

#### 2-(4-Chlorophenyl)-3-(phenylselanyl)benzo[*b*]furan 3l

Yield: 0.035 g (44%). White solid, mp = 76 °C. ^1^H NMR (400 MHz, CDCl_3_) *δ* (ppm) = 8.23 (d, *J* = 7.4 Hz, 2H), 7.56 (d, *J* = 8.2 Hz, 1H), 7.49–7.37 (m, 5H), 7.36–7.28 (m, 2H), 7.24–7.19 (m, 4H). ^13^C NMR (100 MHz, CDCl_3_) *δ* (ppm) = 157.5, 153.9, 136.1, 130.8, 129.7, 129.4, 129.0, 128.5, 127.4, 126.5, 125.5, 125.2, 123.4, 120.4, 111.3, 104.6. MS: *m*/*z* (rel intensity) 302 (M^+^ 100.0), 273 (10.5), 241 (17.1), 225 (36.0), 197 (32.1), 165 (34.9), 152 (10.8), 139 (9.5), 105 (32.1), 77 (13.0), 51 (7.6).

#### 2-(Phenyl)-3-(phenylteluryl)benzo[*b*]furan 3m

Yield: 0.031 g (30%). Orange solid, mp = 80 °C.^2^^1^H NMR (400 MHz, CDCl_3_) *δ* (ppm) = 8.12 (d, *J* = 7.1 Hz, 2H), 7.54–7.53 (m, 2H), 7.47–7.44 (m, 4H), 7.42–7.38 (m, 1H), 7.34 (dt, *J* = 7.5 and 1.4 Hz, 1H), 7.25–7.23 (m, 1H), 7.18–7.08 (m, 3H). ^13^C NMR (100 MHz, CDCl_3_) *δ* (ppm) = 159.2, 154.6, 134.9, 134.3, 130.6, 129.5, 129.3, 128.5, 128.3, 127.2, 125.2, 123.3, 123.1, 114.8, 111.0, 82.6. EM: *m*/*z* (rel intensity) 400 (M^+^ 19.7), 270 (100.0), 241 (19.2), 207 (5.7), 193 (5.9), 165 (63.1), 139 (12.4), 115 (8.8), 77 (21.7), 51 (8.4).

#### 4-(2-Methoxyphenyl)-3-methyl-4-(phenylselanyl)but-3-en-2-one 4a

Yield: 0.037 g (42%). Yellow oil. ^1^H NMR (400 MHz, CDCl_3_) *δ* (ppm) = 7.47 (dd, *J* = 7.7, 1.8 Hz, 1H_11_), 7.40 (ddd, *J* = 8.4 Hz, 7.4 Hz and 1.8 Hz, 1H, H_9_), 7.28–7.25 (m, 2H_14,14′_), 7.19–7.13 (m, 3H_15,15′,16_), 6.92–6.86 (m, 2H, H_8,10_), 3.77 (s, 3H, H_12_), 2.17 (s, 3H_1_), 2.04 (s, 3H, H_4_). ^13^C NMR (100 MHz, CDCl_3_) *δ* (ppm): 194.2 (C_2_), 158.4 (C_7_), 146.2 (C_6_), 133.3 (C_9_), 131.2 (C_11_), 131.1 (C_14,14′_), 128.9 (C_15,15′_), 127.6 (C_5_), 127 (C_3_), 126.3 (C_16_), 120.3 (C_10_), 111.4 (C_8_), 55.7 (C_12_), 24.9 (C_1_), 22.6 (C_4_). MS: *m*/*z* (rel intensity) 346 (19.6), 331 (3.4), 315 (2.9), 265 (3.7), 189 (21.9), 174 (17.8), 158 (20.1), 135 (100.0), 129 (14.1), 105 (3.5), 92 (11.2), 77 (34.4), 51 (4.9). IR (cm^−1^) 2936, 1636, 1479, 1248, 1016, 734. HRMS calculated for C_18_H_19_O_2_Se + Na = 369.0360, found: 369.0366.

#### 1-Methyl-2-phenyl-3-(phenylselanyl)indole 5a

Yield: 0.023 g (25%). Pale yellow oil. ^1^H NMR (500 MHz, CDCl_3_) *δ* (ppm) = 7.66 (d, *J* = 6.5 Hz, 1H), 7.35–7.38 (m, 6H), 7.28–7.31 (m, 1H), 7.18–7.19 (m, 1H), 7.13–7.14 (m, 2H), 7.02–7.07 (m, 3H), 3.67 (s, 3H). ^13^C NMR (125 MHz, CDCl_3_) *δ* (ppm) = 145.8, 137.7, 134.6, 131.2, 130.7, 130.6, 128.8, 128.6, 128.3, 128.1, 125.2, 122.6, 120.8, 120.6, 109.7, 96.3, 31.7. EM: *m*/*z* (rel intensity) 363 (M^+^ 21.0), 283 (100.0), 267 (11.7), 204 (10.8), 190 (3.8), 165 (4.7), 141 (6.3), 77 (5.2).

#### 2-(4-Chlorophenyl)-2-(phenylselanyl)-1-methylindol 5b

Yield: 0.020 g (20%). Yellow solid, mp = 103–104 °C. ^1^H NMR (400 MHz, CDCl_3_) *δ* (ppm) = 7.60 (d, *J* = 7.9 Hz, 1H, H_5_), 7.35–7.32 (m, 3H, H_8,11,11′_), 7.28–7.22 (m, 3H, H_7,12,12′_), 7.13 (ddd, *J* = 7.5 Hz, 7.0 Hz and 1.0 Hz, 1H, H_6_), 7.08–6.99 (m, 5H, H_15–17_), 3.65 (s, 3H, H_1_). ^13^C NMR (100 MHz, CDCl_3_) *δ* (ppm) = 144.4 (C_2_), 137.7 (C_9_), 134.8 (C_13_), 134.3 (C_14_), 132.0 (C_11,11′_), 130.5 (C_4_), 129.6 (C_10_), 128.9 (C_12,12′_), 128.4 (C_15,15′,16_), 128.3 (C_15,15′,16_), 125.3 (C_17_), 122.9 (C_7_), 121.0 (C_6_), 120.7 (C_5_), 109.7 (C_8_), 96.9 (C_3_), 31.7 (C_1_). ^15^N NMR (40 MHz, CDCl_3_) *δ* (ppm) = 130 ppm. EM: *m*/*z* (rel intensity) 397 (M^+^ 23.4), 317 (100.0), 281 (16.7), 267 (8.8), 204 (9.7), 141 (15.1), 77 (3.7).

## Conflicts of interest

There are no conflicts to declare.

## Supplementary Material

RA-010-D0RA01907K-s001
