# Supplementary material for: Synthesis of 3-selanylbenzo[b]furans promoted by SelectFluor®
Source: RSC Adv. 2020 Apr 7;10(24):13975–83. doi: 10.1039/d0ra01907k (PMC9051628; doi:10.1039/d0ra01907k)
Supplement: RA-010-D0RA01907K-s001 [file RA-010-D0RA01907K-s001.pdf]

This journal is © The Royal Society of Chemistry 2020

General Information

Proton nuclear magnetic resonance spectra (^{1}H NMR) were recorded at 400 MHz on a Bruker Avance III HD spectrometer. Chemical shifts (δ) are reported in ppm, and the abbreviations to denote the multiplicity of a particular signal are s (singlet), d (doublet), t (triplet), q (quartet), quint (quintet), sext (sextet), td (triple doublet) and m (multiplet). NMR data are reported as follows: chemical shift (δ), multiplicity, coupling constant (*J*) in Hertz and integrated intensity. ^{1}H NMR chemical shifts were referenced to the tetramethylsilane (TMS; 0.0 ppm) internal standard. Selenium-77 nuclear magnetic resonance spectra (^{77}Se NMR) were recorded at 76 MHz and referenced to the diphenyl diselenide ($C_6H_5SeSeC_6H_5$; 473.0 ppm) external standard. Fluorine-19 nuclear magnetic resonance spectra (^{19}F NMR) were obtained at 376 MHz and referenced to the trifluoro-toluene ($C_6H_5CF_3$; - 63.7 ppm) external standard. Carbon-13 nuclear magnetic resonance spectra (^{13}C NMR) were recorded at 100 MHz and referenced to deuterated solvent ($CDCl_3$; 77.0 ppm or DMSO-d_6; 39.51 ppm or CDCN-d_3; 1.39 ppm). Spectra were recorded in either $CDCl_3$ or DMSO-d_6 solutions. Mass spectra (MS) were obtained on a gas chromatograph coupled to a Shimadzu GCMS-QP2010 mass spectrometer. Fragments are described by its mass/charge ratio (*m*/*z*) with the relative abundance (%) in parentheses. High resolution mass spectra were recorded using a Bruker micrOTOF-QII (source type: APCI). The samples were dissolved in HPLC-grade acetonitrile and injected into the APCI source by means of a syringe pump at a flow rate of 5.0 μL/min. The Compass 1.3 for micrOTOF-Q II software (Bruker daltonics, USA) was used for data acquisition, processing, and isotopic simulations. For the FTIR (Fourier Transform Infrared) in the attenuated total reflection mode (FTIR-ATR), the samples submitted to KI and placed on the crystal surface of a FTIR Bruker Alpha-P spectrometer, obtained from an average of 24 scans at the range of 4000-1500 cm^{-1}. Melting point were determined using a PDF III Marte with 0,1 °C precision. The NMR, IR and Mass analyses were carried out at the Central Analítica – Centro de Ciências Química, Farmacêuticas e de Alimentos – Universidade Federal de Pelotas – UFPel – Pelotas – Brazil. The HRMS analyses were recorded at the Universidade de Caxias do Sul – UCS – Brazil.

The reactions were monitored by thin layer chromatography (TLC) that was performed using Merck silica gel (60 F254), 0.25 mm thickness. For

visualization, TLC plates were either placed under UV light, or stained with iodine vapor, or 5% vanillin in 10% H_2SO_4 and heating. The reactions were monitored by TLC, according on the disappearance of starting materials. Aldrich technical grade silica gel (pore size 60 Å, 230–400 mesh) was used for flash chromatography using hexane/ethyl acetate as eluent. Air- and moisture-sensitive reactions were conducted in flame-dried or oven dried glassware equipped with tightly fitted rubber septa and under a positive atmosphere of dry nitrogen. Acetonitrile was dried over molecular sieves 3A prior to use. All other solvents and reagents that were commercially available (Sigma Aldrich®) were used without any previous treatment. Temperatures above room temperature were maintained by use of a mineral oil bath with an electrically heated coil connected to an adjustable controller.

General Procedure for the Preparation of 2-alkynylanisoles 1 via Sonogashira coupling:[1]

To a two-necked round bottom flask containing $PdCl_2(PPh_3)_2$ (1 mol%) and Et_3N (3 mL) was added 2-bromoanisole (1 mmol) and terminal alkyne (1.5 mmol). The resulting solution was stirred for 5 minutes at room temperature. After this time, was added CuI (2 mol%) and the reaction mixture was allowed to stir at 75 °C for 12 hours. After this time, the mixture was diluted with ethyl acetate (20 mL) and washed with saturated brine (2 x 20 mL). The organic phase was separated, dried over $MgSO_4$ and concentrated under vacuum. The residue was purified by flash chromatography and eluted with hexane.

General procedure for preparation of 3-selanylbenzo[b]furans 3a-k.

Diorganyl disselenide **2** (0,15 mmol) was added to a round botton flask followed by addition of dry MeCN (2 mL). To this solution, selectfluor (0,125 mmol) was added under N_2 atmosphere. As the selectfluor was dissolved the reaction color changed from yellow to red-brown. After 5 min from the addition of selectfluor, a solution of 2-alkynylanisol **1** in dry MeCN (0,25 mmol in 1 mL of dry MeCN) was added to the reaction mixture, the reaction color usually changed from red-brown to clear brown. The reaction progress was monitored by TLC.

[1] Yue, D.; Yao, T.; Larock, R. C. *J. Org. Chem.* **2005**, 70, 10292-10296.

After reaction completion ethyl acetate (20 mL) and distilled water (20 mL) were added and the aquous layer was washed with ethyl acetate (2 x 20 mL). Then, the combined organic layers were then washed with 10 mL of distilled water to remove any remaining MeCN. After removal of the solvent, column cromatography was performed using silica gel and either hexane or a mixture of hexane and ethyl acetate depending on the polarity of the product **3**.

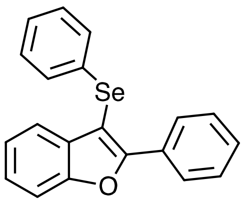

3a (97 %)

2-Phenyl-3-(phenylselanyl)benzo[*b*]furan **3a**:[1,2] Yield: 0.084 g (97%), White solid, mp = 40 - 41 °C.[2] ^{1}H NMR ($CDCl_3$, TMS, 400 MHz) δ (ppm) = 8.12 (d, J = 7.2 Hz, 2H), 7.47 – 7.42 (m, 2H), 7.37 – 7.19 (m, 6H), 7,14 (dt, J = 7.4 and 1.0 Hz, 1H), 7.09 – 7.02 (m, 3H). ^{13}C NMR (100 MHz, $CDCl_3$) δ (ppm) = 157.2, 154.1, 131.8, 131.3, 130.1, 129.3, 129.2, 129.1, 128.4, 127.7, 126.2, 125.2, 123.4, 121.1, 111.1, 99.6. MS: *m*/*z* (rel intensity) 350 (M^+, 32.4); 270 (100,0), 255 (7.8), 241 (17.0), 165 (28.5), 134 (8.4), 115 (4.5), 77 (5.4).

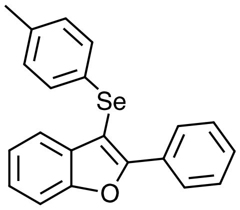

3b (81 %)

2-Phenyl-3-[(4-methylphenyl)selanyl]benzo[*b*]furan **3b**:[3] Yield: 0.073 g (81%); Yellow solid, mp = 77 °C.[2] ^{1}H NMR (400 MHz, $CDCl_3$) δ (ppm) = 8.13 (d, J = 7.2 Hz, 2H), 7.44 (t, J = 8.2 Hz, 2H), 7.38 – 7.34 (m, 2H), 7.33 – 7.24 (m, 1H), 7.22 (td, J = 7.7 Hz and 1.4 Hz, 1H), 7.15 – 7.11 (m, 3H), 6.88 (d, J = 8.0 Hz, 2H), 2.14 (s, 3H). ^{13}C NMR (100 MHz; $CDCl_3$) δ (ppm) = 156.9, 154.0, 136.1, 131.9, 130.1, 130.0, 129.5,129.1, 128.4, 127.7, 127,4, 125.1, 123.3, 121.2, 111.1, 100.1, 20.9.

---

[1] Yue, D.; Yao, T.; Larock, R. C. *J. Org. Chem.* **2005**, 70, 10292-10296.
[2] Gay, R. M.; Manarin, F.; Schneider, C. C.; Barancelli, D. A.; Costa, M. D.; Zeni, G. *J. Org. Chem.* **2010**, 75, 5701–5706.
[3] Du, H.-A., Zhang, X.-G., Tang, R.-Y. Li, J.-H. *J. Org. Chem.* **2009**, 74, 7844–7848.

MS: *m*/*z* (rel intensity) 364 (M^+ 33.1), 284 (100.0), 269 (13.5), 241 (14.6), 165 (24.8), 91 (5.2), 65 (3.1).

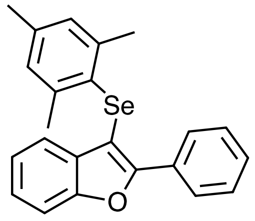

3c (77 %)

2-Phenyl-3-(mesitylselanyl)benzo[*b*]furan **3c**: Yield: 0.072 g (77%); Yellow solid, mp = 145 - 148 °C. ^{1}H NMR (400 MHz, $CDCl_3$) *δ* (ppm) = 8.04 (d, *J* = 7.3 Hz, 2H), 7.40 – 7.34 (m, 3H), 7,29 (m, 1H), 7,10 (dt, *J* = 1.4 and 8,0 Hz, 1H), 6.93 (dt, *J* = 7.5 and 1.0 Hz, 1H), 6.85 (m, 1H), 6.76 (s, 2H), 2.30 (s, 6H), 2.13 (s, 3H). ^{13}C NMR (100 MHz, $CDCl_3$) *δ* (ppm) = 153.9, 153.4, 142.2, 138.1, 131.6, 130.6, 128.9, 128.6, 128.3, 127.4, 126.4, 124.6, 122.8, 120.6, 110.9, 102.3, 24.1, 20.8. MS: *m*/*z* (rel intensity) 392 (M^+, 37.9); 311 (2.3); 281 (4.8); 194 (100.0); 165 (33.3); 139 (5.4); 119 (14.8); 91 (16.7); 77 (10.4); 44 (12.2). HRMS calculated for $C_{23}H_{20}OSe$ 392.0675, found: 392.0676.

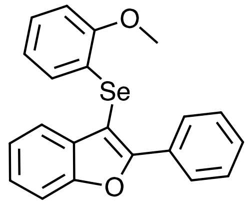

3d (70 %)

3-(2-Methoxyphenylselanyl)-2-(phenyl)benzo[b]furan **3d**:[1] Yield.: 0.063 g (70%), yellow oil. ^{1}H NMR (400 MHz, $CDCl_3$) δ (ppm) = 8.19 (d, *J* = 7.8 Hz, 2H), 7.57 – 7.52 (m, 2H), 7.43 – 7.31 (m, 4H), 7.24 – 7.21 (m, 1H), 7.10 (t, *J* = 7.7 Hz, 1H), 6.84 (d, *J* = 8.1 Hz, 1H), 6.78 (d, *J* = 7.7 Hz), 6.66 (t, *J* = 7.5 Hz, 1H), 3.93 (s, 3H). ^{13}C NMR (100 MHz, $CDCl_3$) δ (ppm) = 158.0, 156.4, 154.2, 132.1, 130.1, 129.2, 128.4, 128.1, 127.8, 126.8, 125.2, 123.4, 121.8, 121.3, 120.6, 111.1, 110.2, 97.8, 55.8. EM: *m*/*z* (rel intensity) 380 (M^+, 85.4), 300 (100.0), 268 (13.8), 257 (17.0), 207 (14.5), 194 (35.7), 165 (64.5), 91 (14.4), 77 (25.6), 63 (12.2). HRMS calculated for $C_{21}H_{16}O_2Se$ + Na = 403.02125, found: 403.0220.

---

[1] Faming Zhuanli Shenqing 2018, CN 108191800 A 20180622

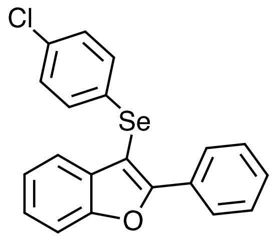

3e (82 %)

3-[(4-Chlorophenyl)selanyl]-2-phenylbenzo[*b*]furan **3e**:[1] Yield: 0.078 g (82%); Yellow solid, mp = 87 °C. ^{1}H NMR (400 MHz, $CDCl_3$) δ (ppm) = 8.08 (d, *J* = 7.0 Hz, 2H), 7,46 (d, *J* = 8.2 Hz, 1H), 7.40 – 7.27 (m, 4H), 7.24 (td, 7.7 Hz, 1.4 Hz, 1 H), 7.16 – 7.09 (m, 3H), 7.03 – 7.00 (m, 2H). ^{13}C NMR (100 MHz, $CDCl_3$) δ (ppm) = 157.3, 154.0, 132.3, 131.5, 130.4, 129.9, 129.5, 129.4, 129.3, 128.4, 127.7, 125.3, 123.5, 120.9, 111.2, 99.3. MS: *m*/*z* (rel intensity) 384 (M^+; 37.2); 304 (100.0), 268 (22.1), 241 (18.6), 165 (38.8), 134 (13.5), 63 (3.7).

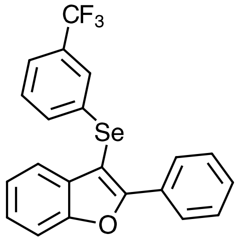

3f (85 %)

2-Phenyl-3-[(3-trifluormethylphenyl)selanyl]benzo[*b*]furan **3f**:[1] Yield.: 0.088 g (85%); Yellow solid, mp = 80 °C. ^{1}H NMR (400 MHz, $CDCl_3$) δ (ppm) = 8.17 (d, *J* = 7.3 Hz, 2H), 7.61 – 7.49 (m, 2H), 7.49 – 7.32 (m, 7H), 7.26 – 7.18 (m, 2H). ^{13}C NMR (100 MHz, $CDCl_3$) δ (ppm) = 157.7, 154.2, 132.7, 132.0, 131.5 (q, *J* = 34.4 Hz), 131.4, 129.8, 129.6, 129.5, 128.5, 127.8, 125.6 (q, *J* = 3.6 Hz), 125.5, 123.0 (q, *J* = 272.9), 123.6, 122.9 (q, *J* = 3.6 Hz), 120.9, 111.3, 98.7. MS: *m*/*z* (rel intensity) 418 (M^+ 35.6), 338 (100.0), 309 (7.3), 268 (4.3), 241 (6.7), 165 (29.8), 139 (6.1), 115 (3.9).

---

[1] Gay, R. M.; Manarin, F.; Schneider, C. C.; Barancelli, D. A.; Costa, M. D.; Zeni, G. *J. Org. Chem.* **2010**, 75, 5701–5706.

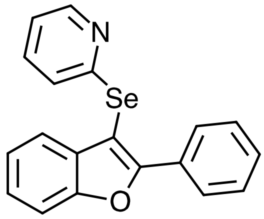

3g (40 %)

2-Phenyl-3-(2-pyridylselanyl)benzo[*b*]furan **3g**: Yield: 0.024 g (40%), Yellow solid, mp = 46 °C. ^{1}H NMR (400 MHz, $CDCl_3$) δ (ppm) = 8.45 (ddd, *J* = 4.8, 1.8 , 0.8 Hz, 1H), 8.23 – 8.20 (m, 2 H); 7.60 – 7.55 (m, 2H), 7.47 – 7.35 (m, 4H), 7.32 – 7.29 (m, 2H), 7.01 (ddd, *J* = 7.4, 4.9, 1 Hz, 1H), 6.9 (dt, *J* = 8.0, 1.0 Hz). ^{13}C NMR (100 MHz, $CDCl_3$) δ (ppm) = 157.6, 157.4, 154.2, 150.0, 136.9, 131.7, 129.9, 129.5, 128.5, 127.8, 125.4, 123.6, 122.9, 121.1, 120.5, 111.3, 99.0. HRMS calculated for $C_{18}H_{13}NOSe$ + Na = 374.0059, found: 374.0055.

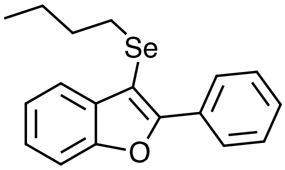

3h (78 %)

3-(Butylselanyl)-2-phenylbenzo[*b*]furan **3h**:[1] Yield: 0.066 g (78%), Yellow oil. ^{1}H NMR (400 MHz, DMSO-d_6) δ (ppm) = 8.19 (d, *J* = 7.8 Hz, 2H), 7.64 (d, *J* = 7.8 Hz, 2H), 7.52 – 7.55 (m, 2H), 7.47 – 7.43 (m, 1H), 7.40 – 7.32 (m, 1 H), 2.80 (t, *J* = 7.2 Hz, 2H), 1.43 (quint, *J* = 7.1 Hz, 2H), 1.24 (sext, *J* = 7.3 Hz, 2H), 0.68 (t, *J* = 7.3 Hz, 2H). ^{13}C NMR (100 MHz, DMSO-d_6) δ (ppm) = 155.33, 153.26, 131.88, 129.76, 129.29, 128.65, 127.30, 125.44, 123.58, 120.73, 111.31, 99.90, 31.76, 27.65, 21.91, 13.22. EM: *m*/*z* (rel intensity) 330 (M^+, 35.3), 274 (11.7), 245 (12.5), 194 (100.0), 165 (31.8), 41 (5.9).

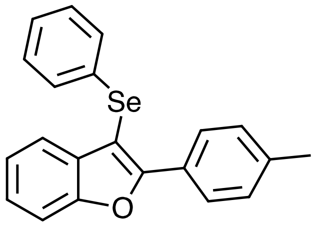

3i (67 %)

3-(Phenylselanyl)-2-(4-methylphenyl)benzo[b]furan **3i**:[1] Yield.: 0.060 g (67%), White solid, mp = 62 °C. ^{1}H NMR (400 MHz, $CDCl_3$) δ (ppm) = 8.10 (d, *J* = 8.2 Hz, 2H), 7.55 – 7.49 (m, 2H), 7.34 – 7.20 (m, 6H), 7.18 – 7.10 (m, 3H), 2.39 (s, 3H). ^{13}C NMR (100 MHz, $CDCl_3$) δ (ppm) = 157.5, 154.0, 139.4, 131.9, 131.5, 129.0, 127.6, 127.3, 126.1, 124.9, 123.3, 121.0, 111.0, 98.8, 21.4. MS: *m*/*z* (rel intensity) 364 (31), 363 (4), 284 (100), 269 (11), 255 (9), 241 (13), 178 (33), 165 (4), 15 (1), 77 (22).

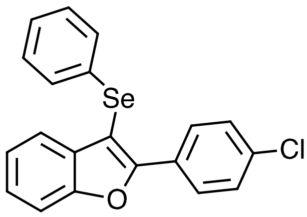

3j (70 %)

2-(4-Chlorophenyl)-3-(phenylselanyl)benzo[*b*]furan **3j**:[2] Yield: 0.069 g (70%), Yellow solid, mp = 71-73 °C. ^{1}H NMR (400 MHz, $CDCl_3$) *δ* (ppm) = 8.07 (d, *J* = 8,7 Hz, 2H), 7.44 – 7.41 (m, 2H), 7.30 (d, *J* = 8.7 Hz, 2H), 7.23 (t, *J* = 7.7 Hz, 1H), 7.19 – 7.11 (m, 3H), 7.08 – 7.01 (m, 3H). ^{13}C NMR (100 MHz, $CDCl_3$) *δ* (ppm) = 155.9, 154.0, 135.2, 131.8, 131.1, 129.3, 129.2, 128.9, 128.7, 128.5, 126.4, 125.4, 123.5, 121.2, 111.2, 100.2. MS: *m*/*z* (rel intensity) 384 (M^+, 51.34), 304 (100.0), 281 (7.35), 268 (25.64), 241 (19.30), 207 (17.43), 199 (16.27), 163 (25.01), 134 (7.98), 73 (10.12).

---

[1] Kazmierczak, J. C.; Recch, A. M. S.; Gritzenco, F.; Balbom, E. B.; Barcellos, T.; Sperança, A.; Godoi, B. *Euro J. Org. Chem.* **2017**, 6382-6389.
[2] L. Chen, L. Xiaoyan, X. Jianhua, L. Yang, Z. Peng, X. Yiwen. *Faming Zhuanli Shenqing*, 2018, CN 108191800 A 20180622.

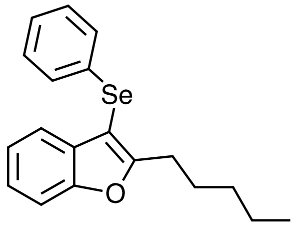

3k (72 %)

2-(^{n}Pentyl)-3-(phenylselanyl)benzo[b]furan **3k**: Yield.: 0.067 g (72%), Clear oil. ^{1}H NMR (400 MHz, $CDCl_3$) δ (ppm) = 7.47 – 7.42 (m, 2H), 7.28 – 7.22 (m, 4H), 7.18 – 7.10 (m, 3H), 2.97 (t, J = 7.5 Hz, 2H), 1.73 (p, *J* = 6.8 Hz, 1H), 1.32 – 1.29 (m, 4H), 0.84 (t, *J* = 6.5 Hz, 3H). ^{13}C NMR (100 MHz, $CDCl_3$) δ (ppm) = 163.8, 154.4, 131.8, 130.7, 129.13, 129.10, 126.0, 124.0, 123.0, 120.3, 110.9, 100.0, 31.3, 27.9, 27.3, 22.3, 13.9. EM: *m*/*z* (rel intensity) 344 (M^+, 80.1), 287 (36.2), 264 (41.0), 207 (100.0), 178 (33.6), 131 (37.1), 115 (8.5), 102 (8.7), 77 (10.0). HRMS calculated for $C_{19}H_{20}OSe$ + Na = 367.05764, found: 367.0549.

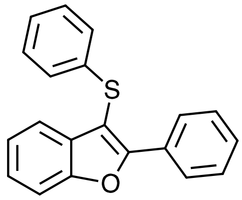

3l (44 %)

2-(4-Chlorophenyl)-3-(phenylselanyl)benzo[*b*]furan **3l**:[1] Yield: 0.035 g (44%), White solid, mp = 76 °C. ^{1}H NMR (400 MHz, $CDCl_3$) *δ* (ppm) = 8.23 (d, *J* = 7.4 Hz, 2H), 7.56 (d, *J* = 8.2 Hz, 1H), 7.49 – 7.37 (m, 5H), 7.36 – 7.28 (m, 2H), 7.24 – 7.19 (m, 4H). ^{13}C NMR (100 MHz, $CDCl_3$) *δ* (ppm) = 157.5, 153.9, 136.1, 130.8, 129.7, 129.4, 129.0, 128.5, 127.4, 126.5, 125.5, 125.2, 123.4, 120.4, 111.3. 104.6. MS: *m*/*z* (rel intensity) 302 (M^+, 100.0), 273 (10.5), 241 (17.1), 225 (36.0), 197 (32.1), 165 (34.9), 152 (10.8), 139 (9.5), 105 (32.1), 77 (13.0), 51 (7.6).

[1] Du, H.-A.; Zhang, X.-G.; Tang, R.-Y.; Li, J.-H. *J. Org. Chem.* **2009**, 74, 7844–7848.

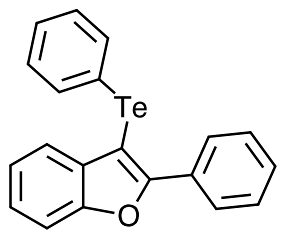

3m (30 %)

2-(Phenyl)-3-(phenylteluryl)benzo[*b*]furan **3m**:[1] Yield: 0.031 g (30%), Orange solid, mp = 80 °C.[2] ^{1}H NMR (400 MHz, $CDCl_3$) δ (ppm) = 8.12 (d, J = 7.1 Hz, 2H), 7.54 – 7.53 (m, 2H), 7.47 – 7.44 (m, 4H), 7.42 – 7.38 (m, 1H), 7.34 (dt, J = 7.5 and 1.4 Hz, 1H), 7.25 – 7.23 (m, 1H), 7.18 – 7.08 (m, 3H). ^{13}C NMR (100 MHz, $CDCl_3$) δ (ppm) = 159.2, 154.6, 134.9, 134.3, 130.6, 129.5, 129.3, 128.5, 128.3, 127.2, 125.2, 123.3, 123.1, 114.8, 111.0, 82.6. EM: *m*/*z* (rel intensity) 400 (M^+, 19.7), 270 (100.0), 241 (19.2), 207 (5.7), 193 (5.9), 165 (63.1), 139 (12.4), 115 (8.8), 77 (21.7), 51 (8.4).

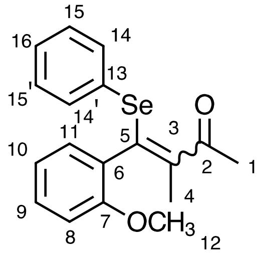

4a (52 %)

4-(2-Methoxyphenyl)-3-methyl-4-(phenylselanyl)but-3-en-2-one **4a**: Yield: 0.037 g (42%), Yelow oil. ^{1}H NMR (400 MHz, $CDCl_3$) δ (ppm) = 7.47 (dd, J = 7.7, 1.8 Hz, $1H_{11}$), 7.40 (ddd, J = 8.4 Hz, 7.4 Hz and 1.8 Hz, 1H, H_9), 7.28 – 7.25 (m, $2H_{14,14'}$), 7.19 – 7.13 (m, $3H_{15,15',16}$), 6.92 – 6.86 (m, 2H, $H_{8,10}$), 3.77 (s, 3H, H_{12}), 2.17 (s, $3H_1$), 2.04 (s, 3H, H_4). ^{13}C NMR (100 MHz, $CDCl_3$) δ (ppm): 194.2 (C_2), 158.4 (C_7), 146.2 (C_6), 133.3 (C_9), 131.2 (C_{11}), 131.1 ($C_{14,14'}$), 128.9 ($C_{15,15'}$), 127.6 (C_5), 127 (C_3), 126.3 (C_{16}), 120.3 (C_{10}), 111.4 (C_8), 55.7 (C_{12}), 24.9 (C_1), 22.6 (C_4). MS: *m*/*z* (rel intensity) 346 (19.6), 331 (3.4), 315 (2.9), 265 (3.7), 189 (21.9), 174 (17.8), 158 (20.1), 135 (100.0), 129 (14.1), 105 (3.5), 92 (11.2), 77 (34.4), 51 (4.9). IR (cm^{-1}) 2936, 1636, 1479, 1248, 1016, 734. HRMS calculated for $C_{18}H_{19}O_2Se$ + Na = 369.0360, found: 369.0366.

---

[1] Gay, R. M.; Manarin, F.; Schneider, C. C.; Barancelli, D. A.; Costa, M. D.; Zeni, G. *J. Org. Chem.* **2010**, 75, 5701–5706.

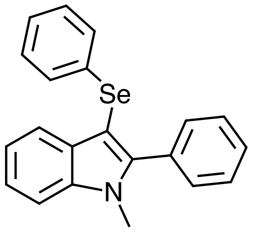

5a (25 %)

1-Methyl-2-phenyl-3-(phenylselanyl)indole **5a**:[1] Yield.: 0.023 g (25%), Pale yellow oil. ^{1}H NMR (500 MHz, $CDCl_3$) δ (ppm) = 7.66 (d, J = 6.5 Hz, 1H), 7.35-7.38 (m, 6H), 7.28-7.31 (m, 1H), 7.18-7.19 (m, 1H), 7.13-7.14 (m, 2H), 7.02-7.07 (m, 3H), 3.67 (s, 3H). ^{13}C NMR (125 MHz, $CDCl_3$) δ (ppm) = 145.8, 137.7, 134.6, 131.2, 130.7, 130.6, 128.8, 128.6, 128.3, 128.1, 125.2, 122.6, 120.8, 120.6, 109.7, 96.3, 31.7. EM: *m*/*z* (rel intensity) 363 (M^+ 21.0), 283 (100.0), 267 (11.7), 204 (10.8), 190 (3.8), 165 (4.7), 141 (6.3), 77 (5.2).

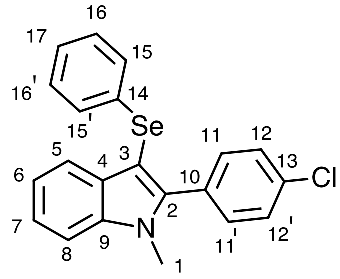

5b (20 %)

2-(4-Chlorophenyl)-2-(phenylselanyl)-1-methylindol **5b**:[2] Yield: 0.020 g (20%), Yellow solid, mp = 103 - 104 °C. ^{1}H NMR (400 MHz, $CDCl_3$) *δ* (ppm) = 7.60 (d, *J* = 7.9 Hz, 1H, H_5), 7.35 – 7.32 (m, 3H, $H_{8,11,11'}$), 7.28 – 7.22 (m, 3H, $H_{7,12,12'}$), 7.13 (ddd, *J* = 7.5 Hz, 7.0 Hz and 1.0 Hz, 1H, H_6), 7.08 – 6.99 (m, 5H, $H_{15\text{-}17}$), 3.65 (s, 3H, H_1). ^{13}C NMR (100 MHz, $CDCl_3$) *δ* (ppm) = 144.4 (C_2), 137.7 (C_9), 134.8 (C_{13}), 134.3 (C_{14}), 132.0 ($C_{11,11'}$), 130.5 (C_4), 129.6 (C_{10}), 128.9 ($C_{12,12'}$), 128.4 ($C_{15,15',16}$), 128.3 ($C_{15,15',16}$), 125.3 (C_{17}), 122.9 (C_7), 121.0 (C_6), 120.7 (C_5), 109.7 (C_8), 96.9 (C_3), 31.7 (C_1). ^{15}N NMR (40 MHz, $CDCl_3$) *δ* (ppm) = 130 ppm. EM: *m*/*z* (rel intensity) 397 (M^+, 23.4), 317 (100.0), 281 (16.7), 267 (8.8), 204 (9.7), 141 (15.1), 77 (3.7).

---

[1] Kota Nishino, Shouya Tsukahara, Yohei Ogiwara, Norio Sakai. Eur. J. Org. Chem. 2019, 1588–1593.

[2] Esperança, A.; Godoi. B.; Menezes, P. H.; Zeni, G. *Synlett*. **2013**, 24, 1125-1132.

SELECTED SPECTRA

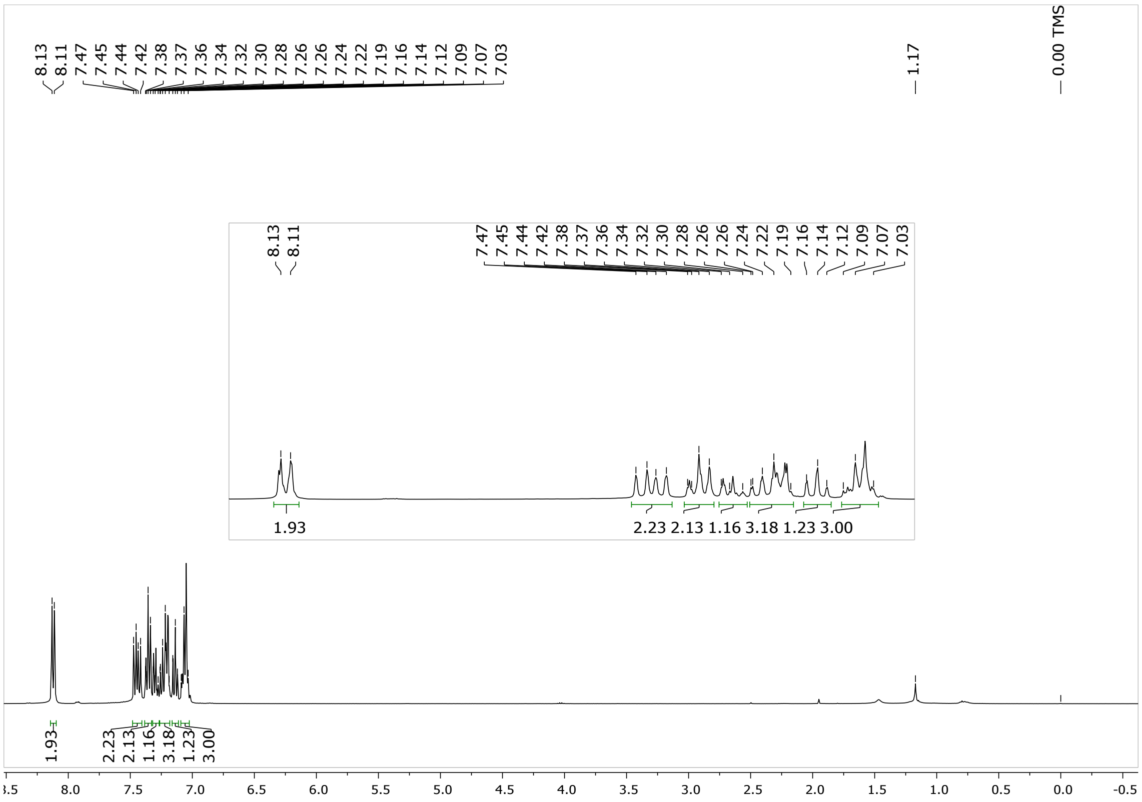

^{1}H NMR (400 MHz, $CDCl_3$) spectrum of compound **3a**.

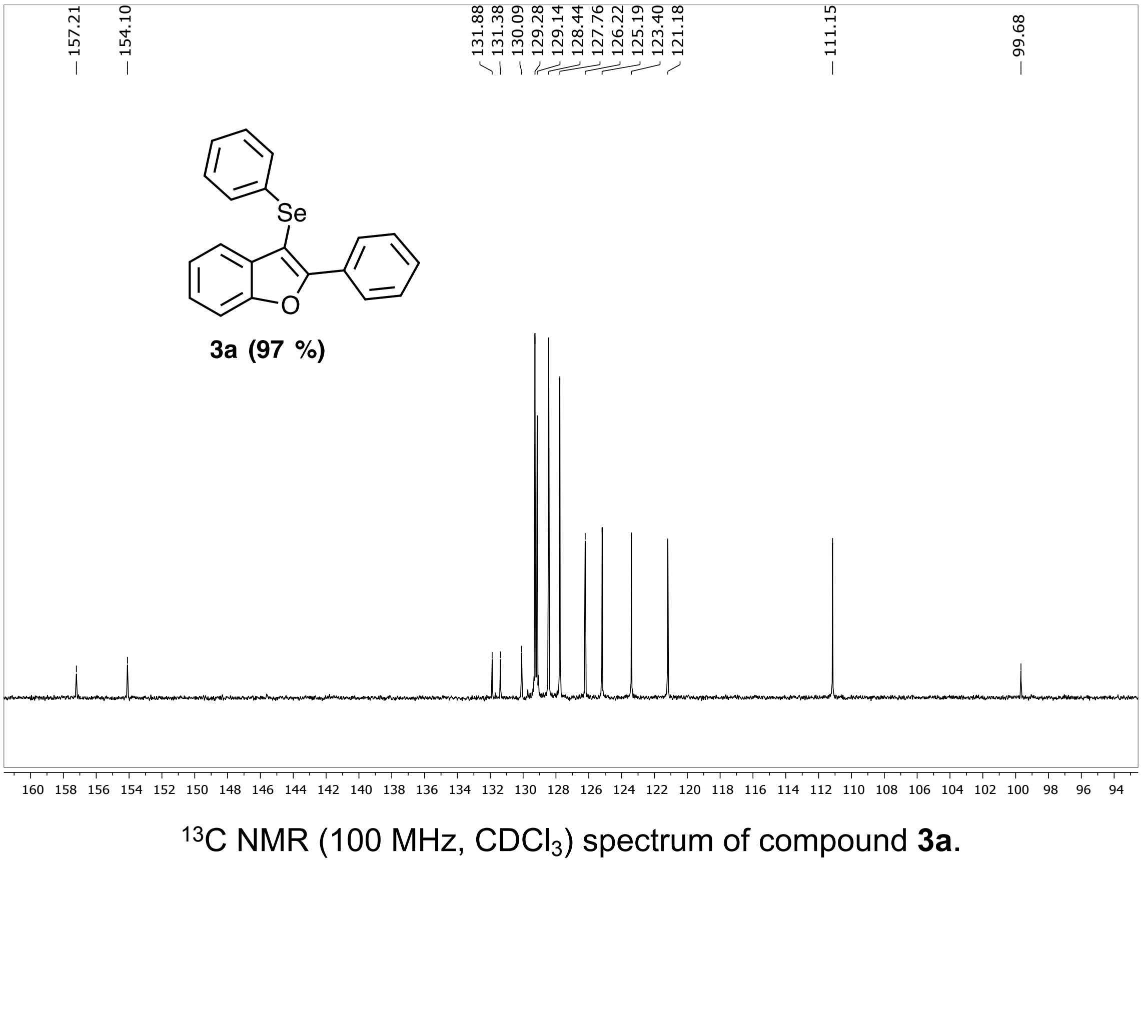

^{13}C NMR (100 MHz, $CDCl_3$) spectrum of compound **3a**.

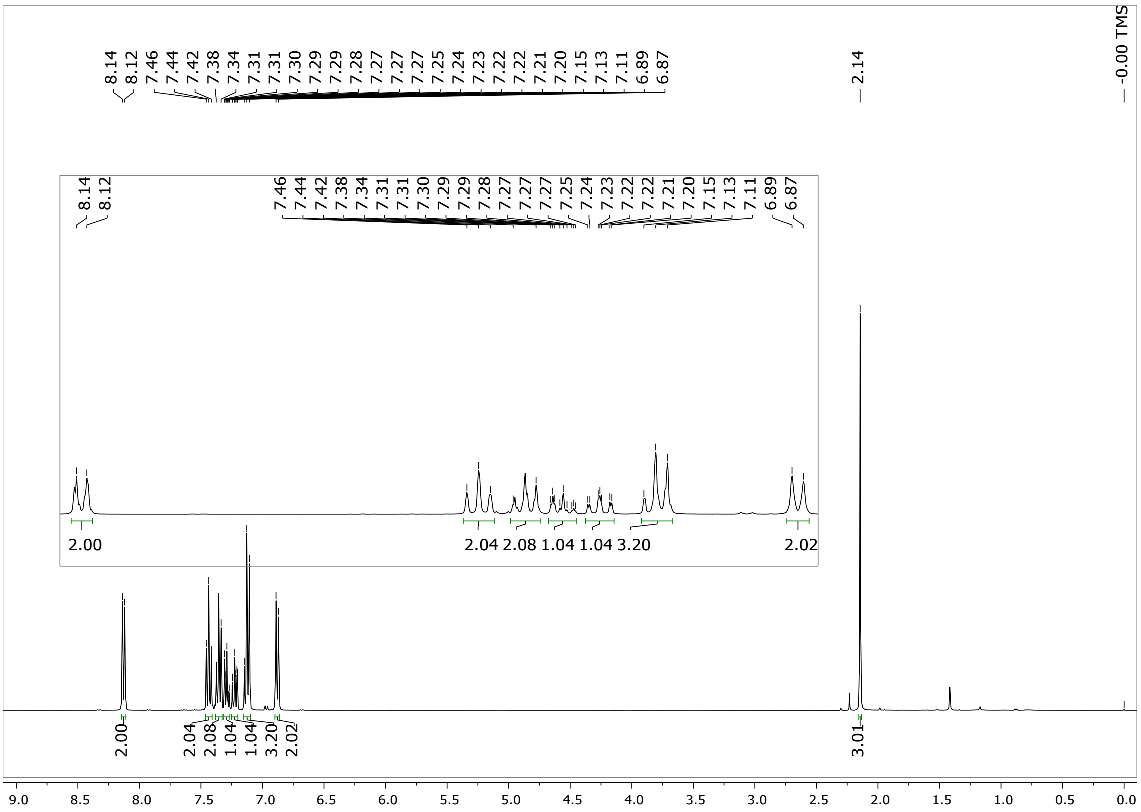

^{1}H NMR (400 MHz, $CDCl_3$) spectrum of compound **3b**.

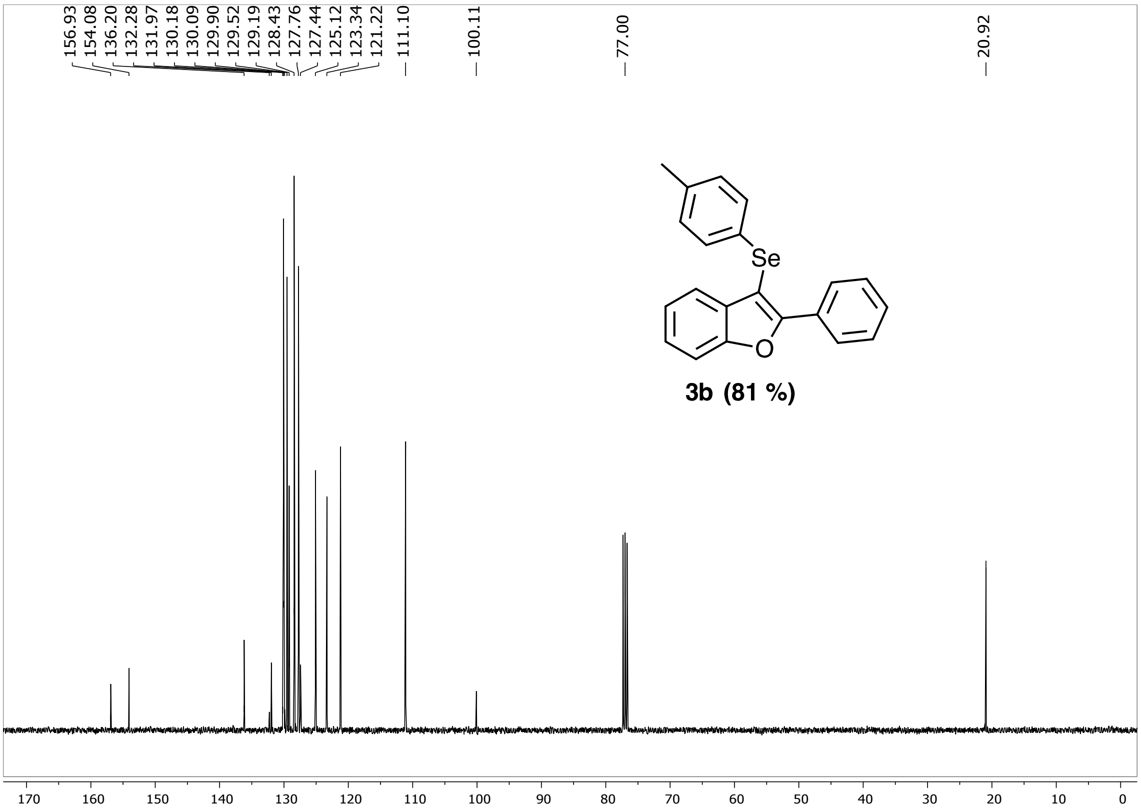

^{13}C NMR (100 MHz, $CDCl_3$) spectrum of compound **3b**.

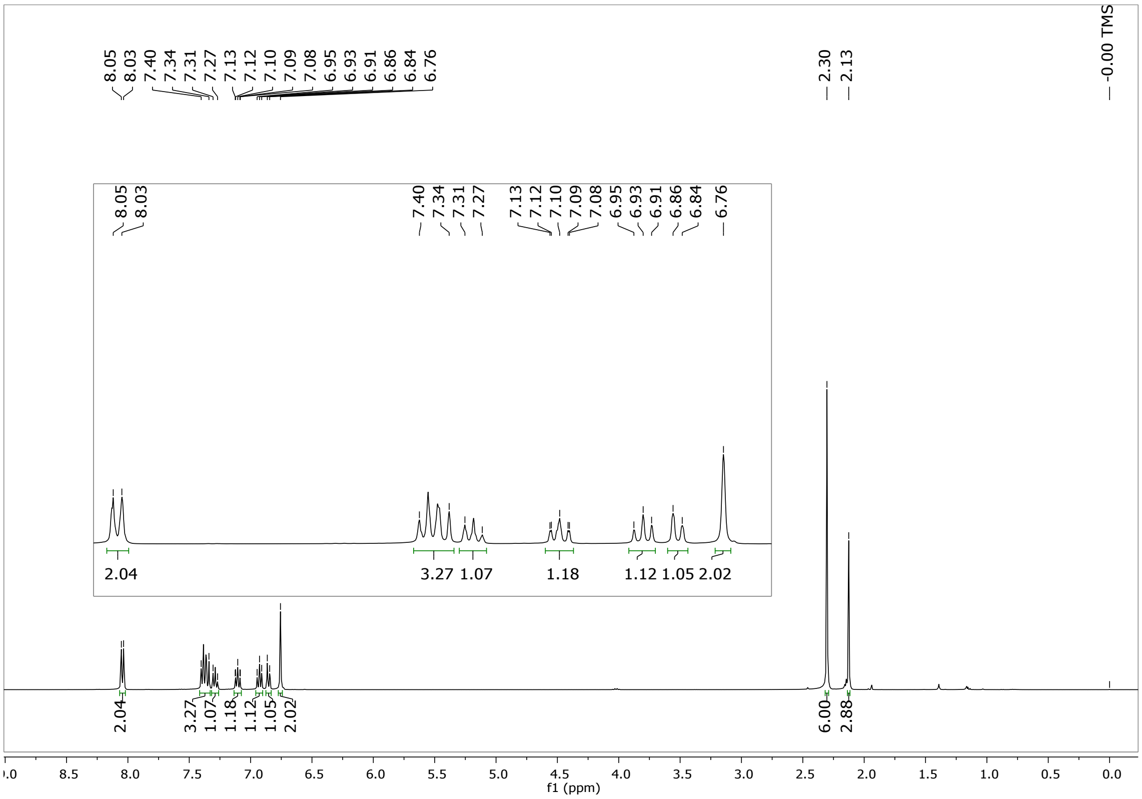

^{1}H NMR (400 MHz, $CDCl_3$) spectrum of compound **3c**.

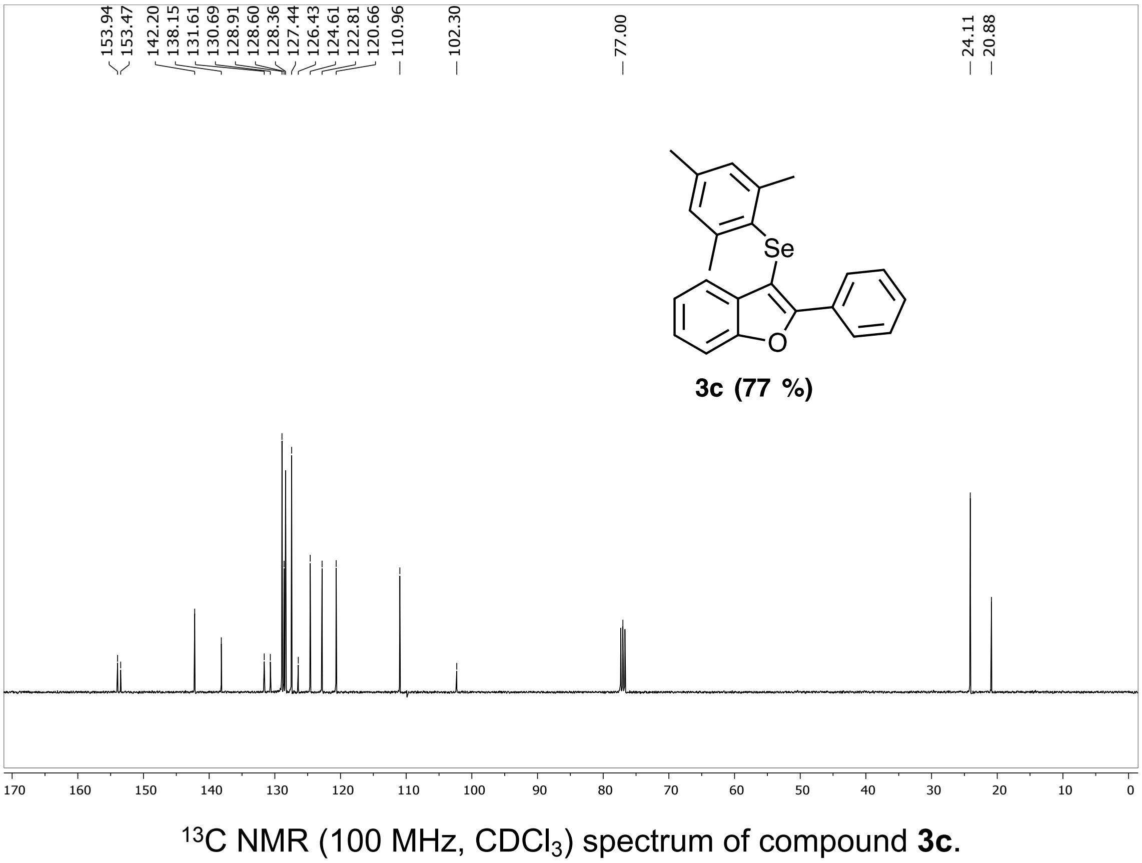

^{13}C NMR (100 MHz, $CDCl_3$) spectrum of compound **3c**.

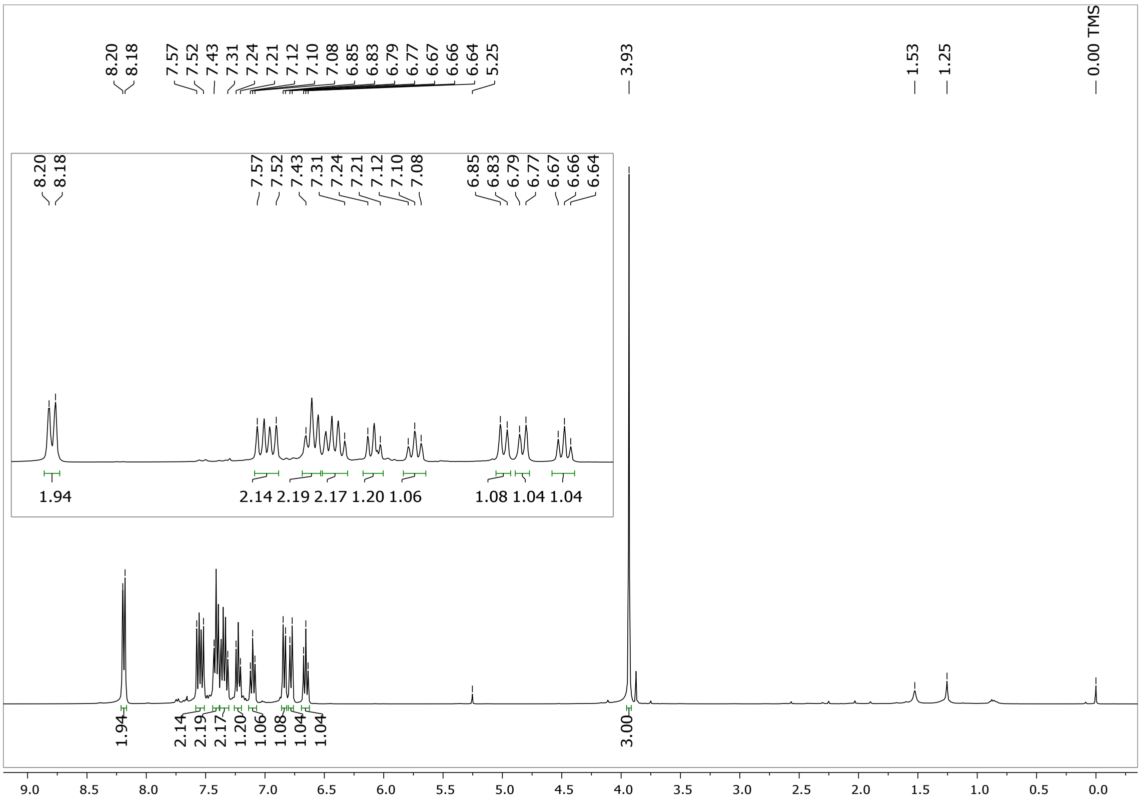

^{1}H NMR (400 MHz, $CDCl_3$) spectrum of compound **3d**.

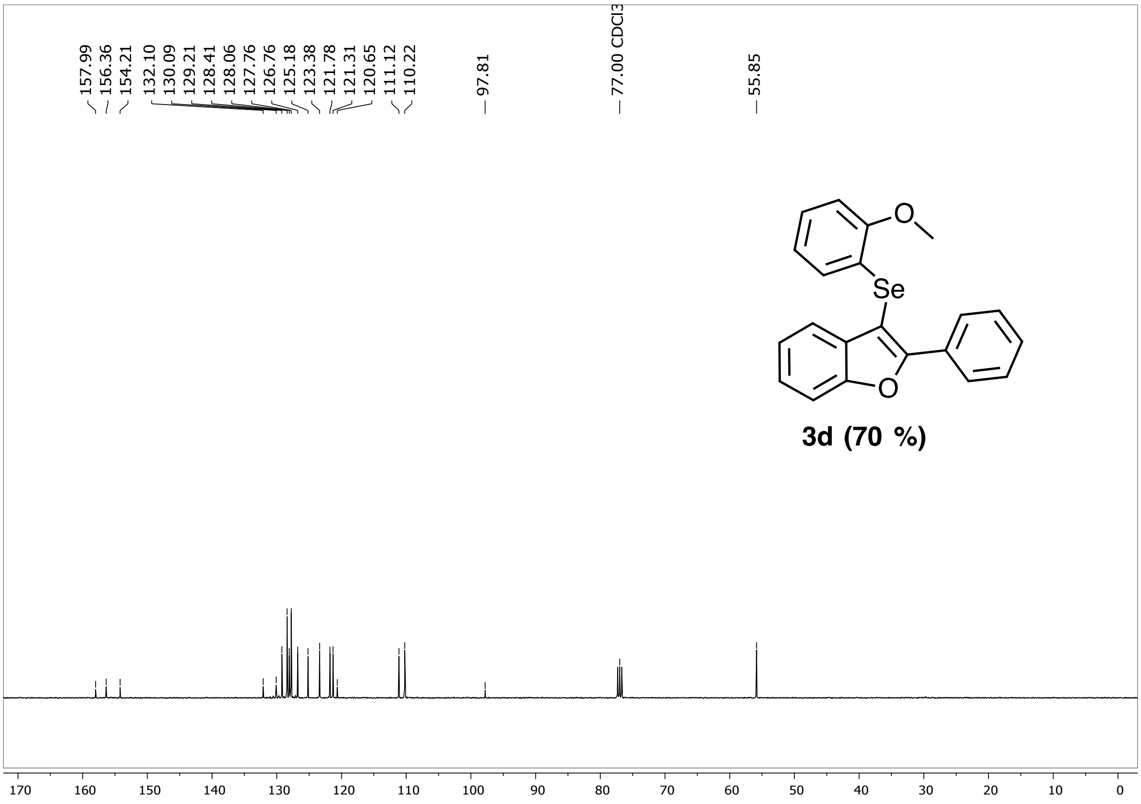

^{13}C NMR (100 MHz, $CDCl_3$) spectrum of compound **3d**.

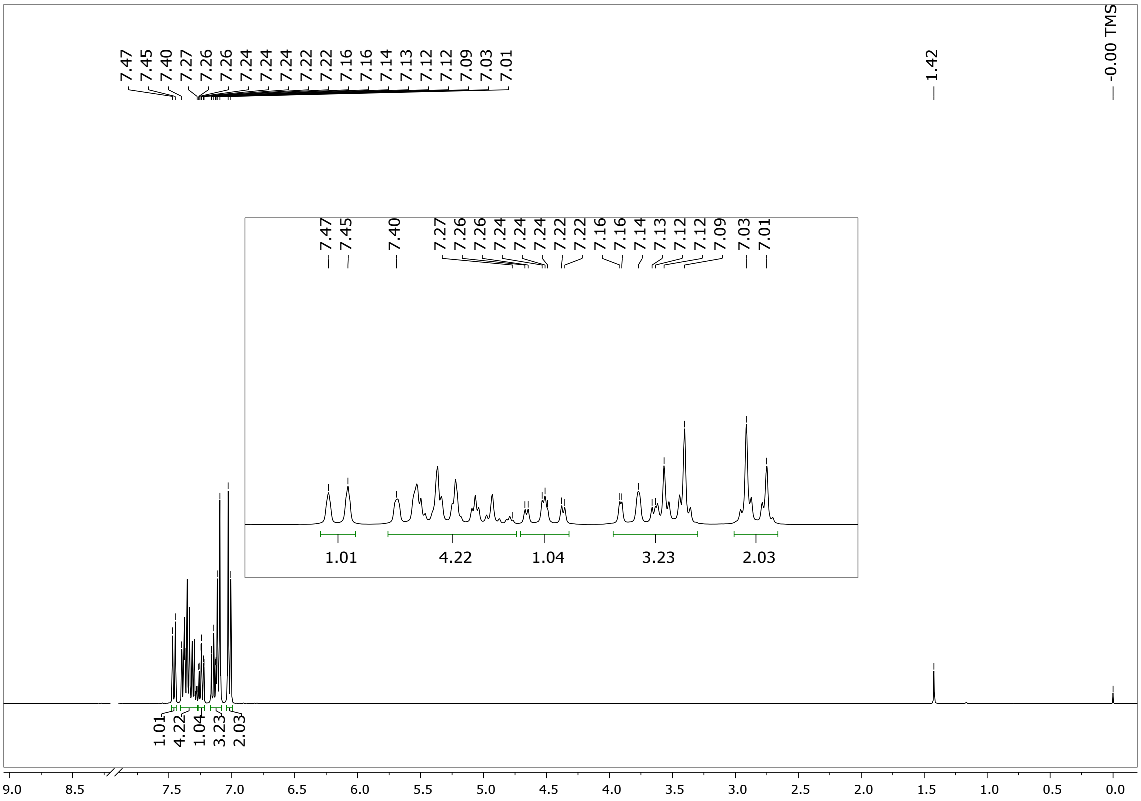

^{1}H NMR (400 MHz, $CDCl_3$) spectrum of compound **3e**.

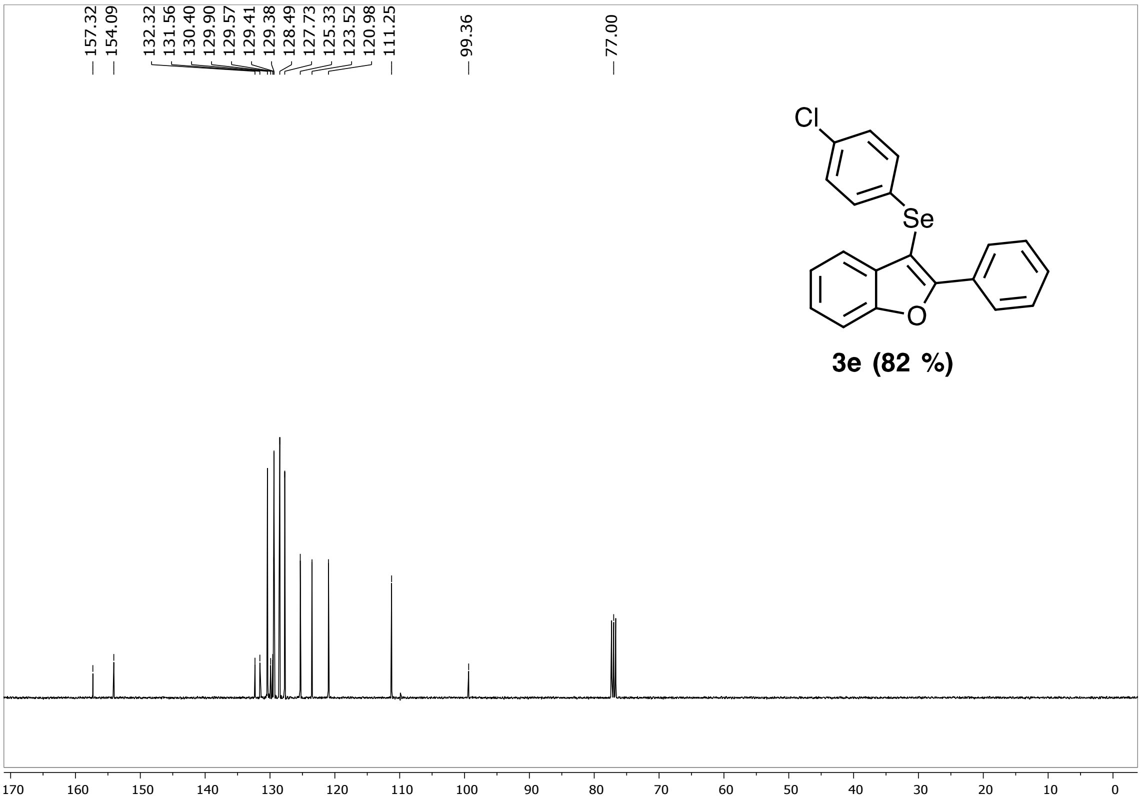

^{13}C NMR (100 MHz, $CDCl_3$) spectrum of compound **3e**.

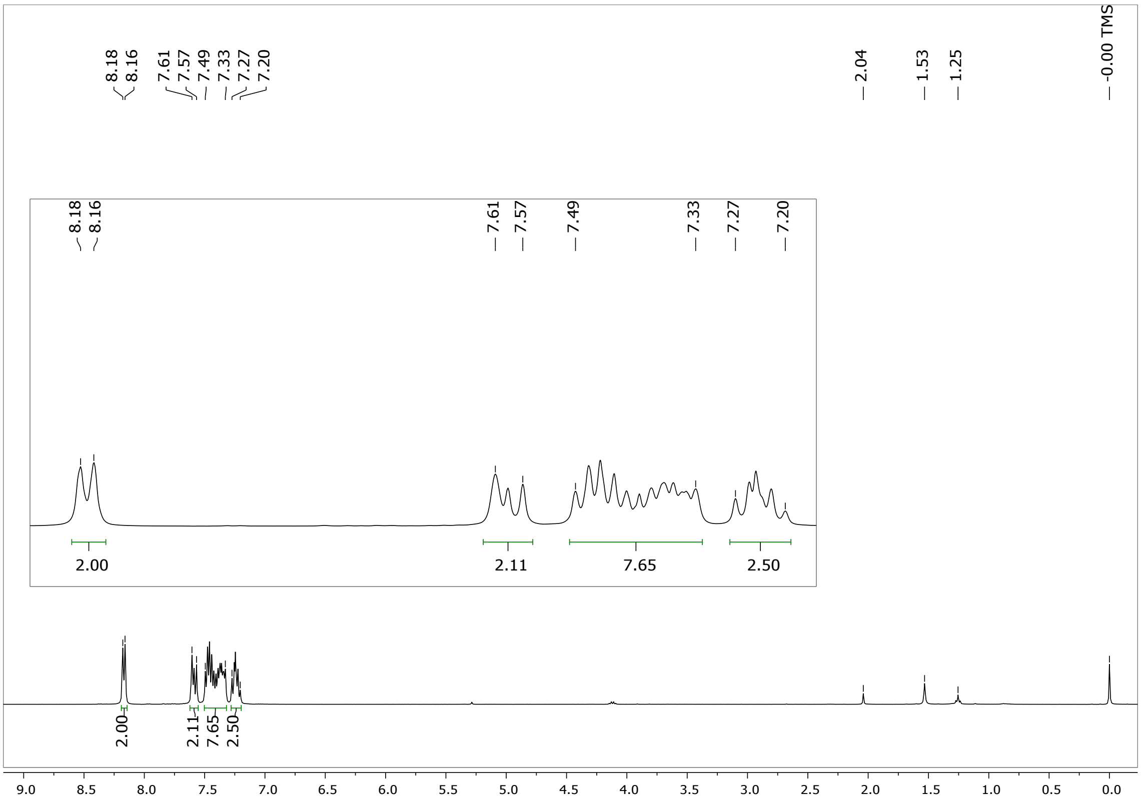

^{1}H NMR (400 MHz, $CDCl_3$) spectrum of compound **3f**.

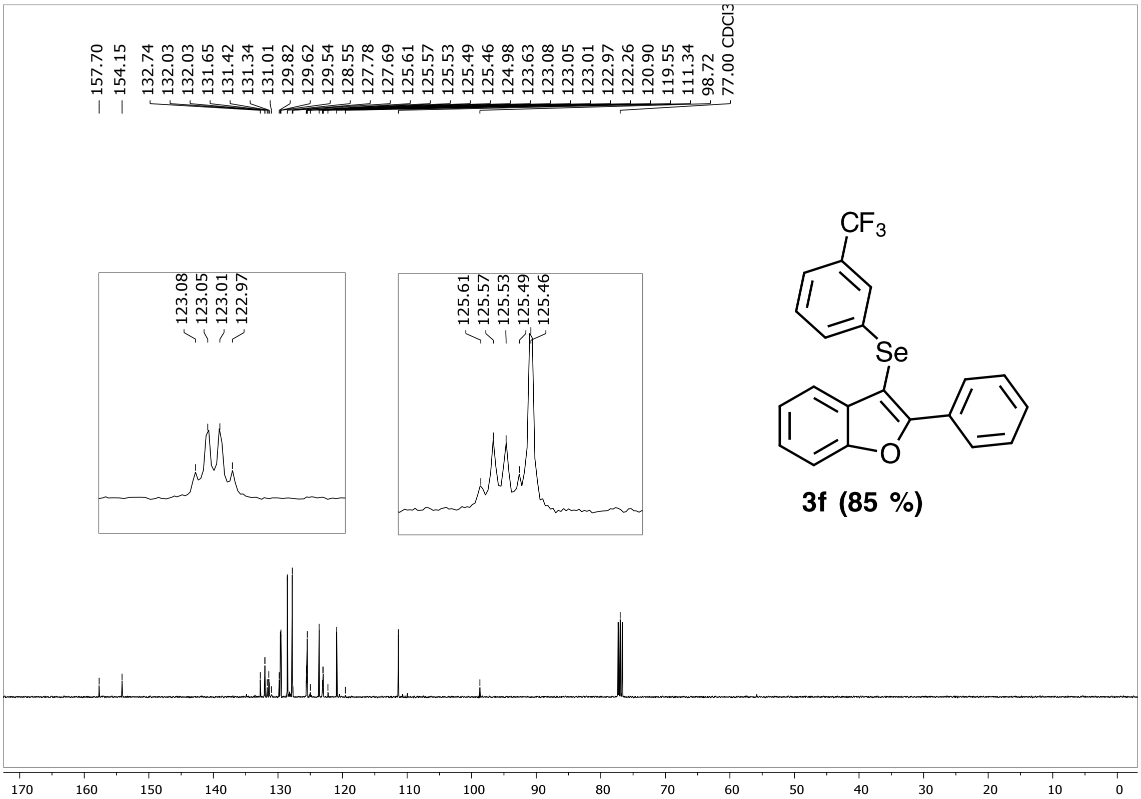

3f (85 %)

^{13}C NMR (100 MHz, $CDCl_3$) spectrum of compound **3f**.

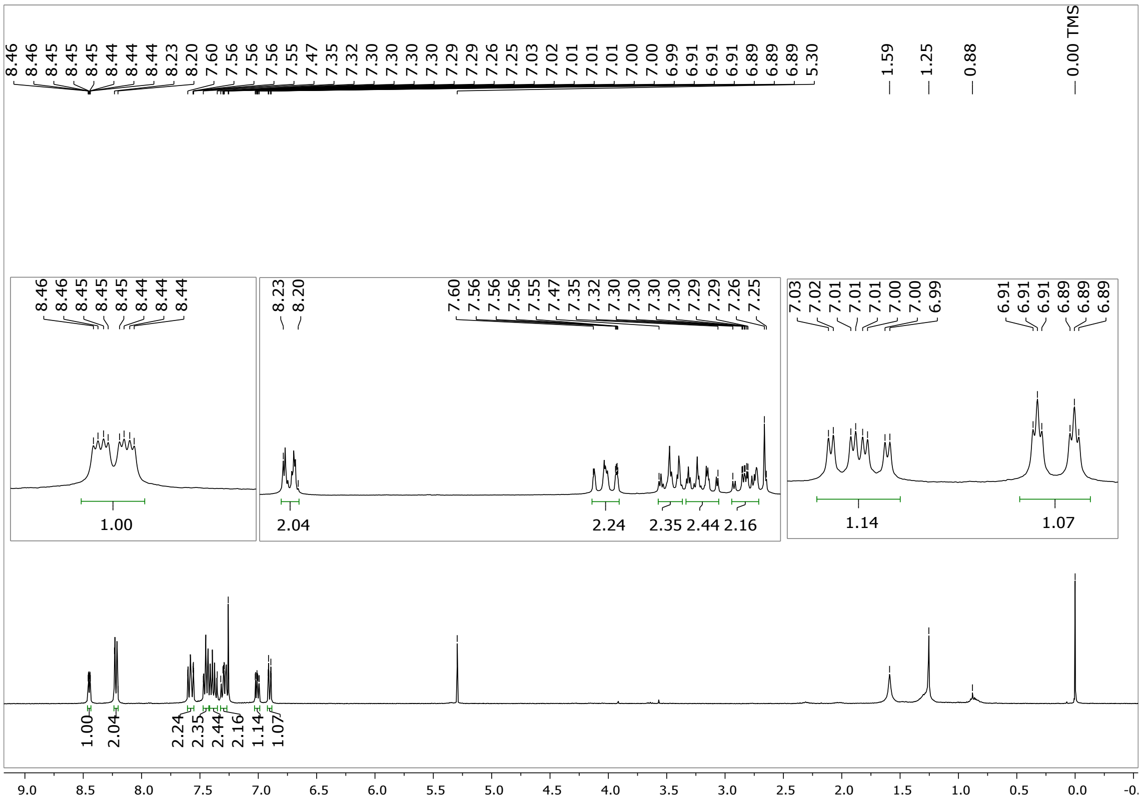

^{1}H NMR (400 MHz, $CDCl_3$) spectrum of compound **3g**.

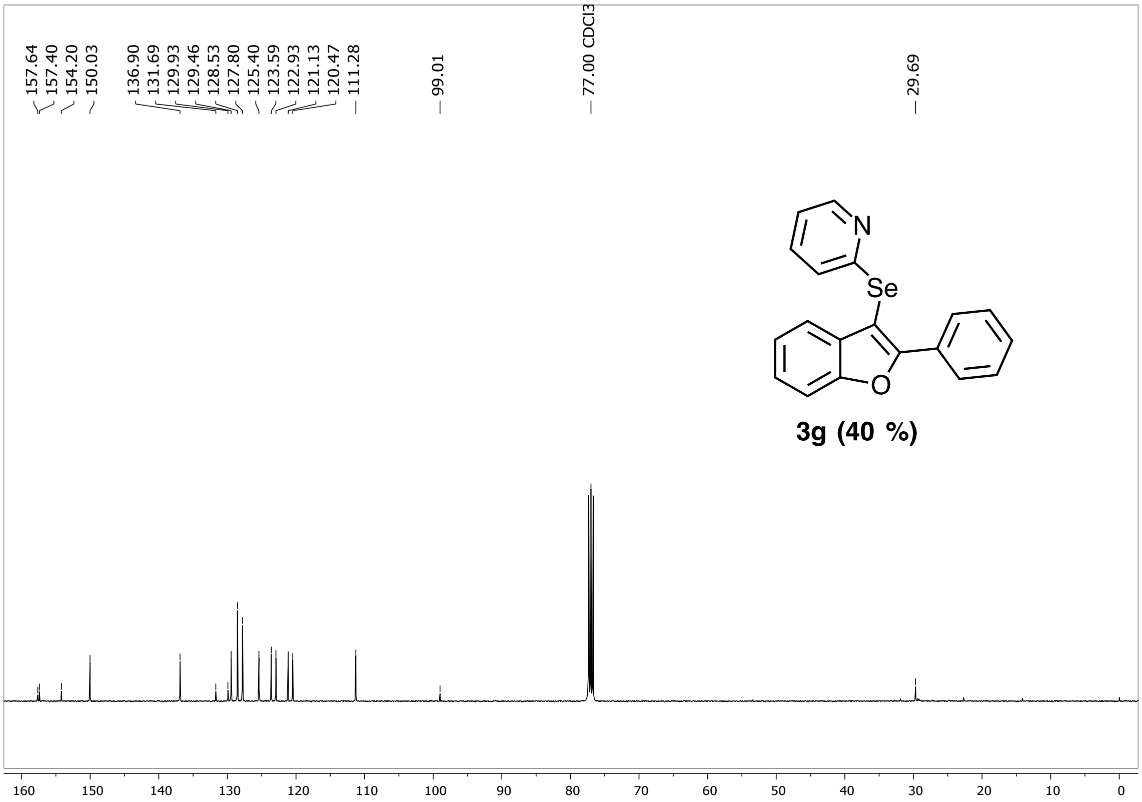

^{13}C NMR (100 MHz, $CDCl_3$) spectrum of compound **3g**.

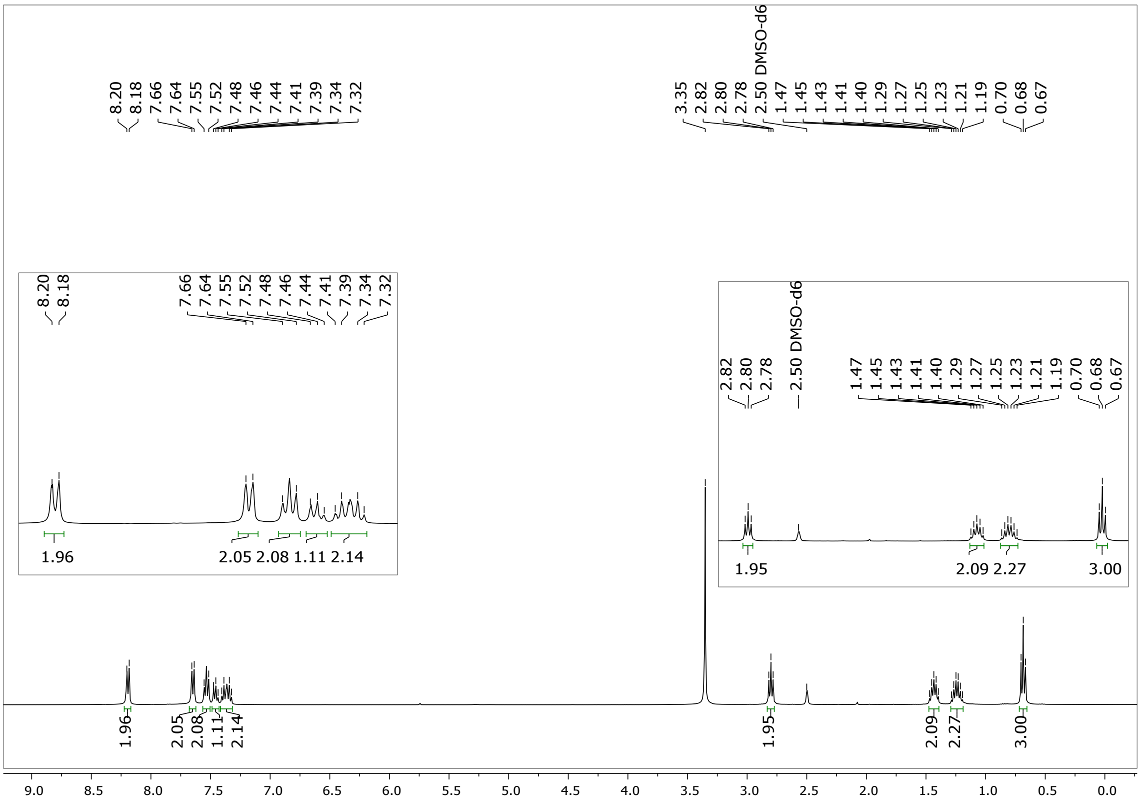

^{1}H NMR (400 MHz, DMSO-$_{d6}$) spectrum of compound **3h**.

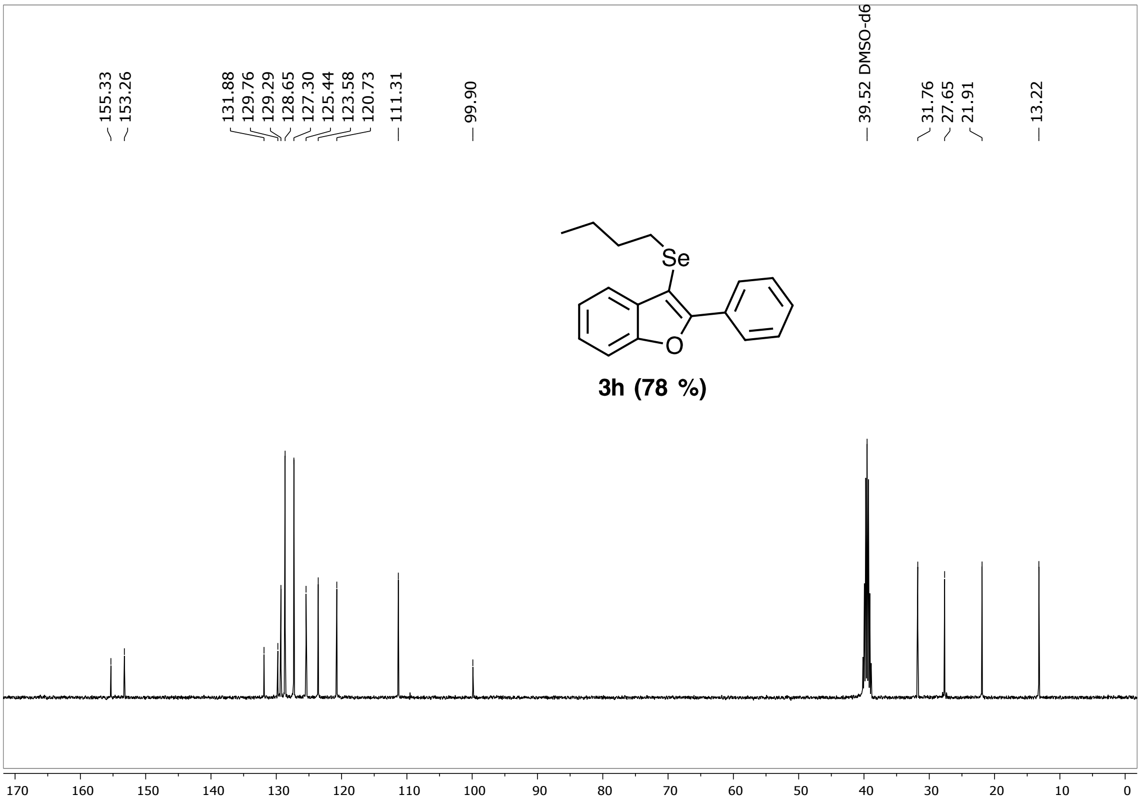

^{13}C NMR (100 MHz, DMSO-$_{d6}$) spectrum of compound **3h**.

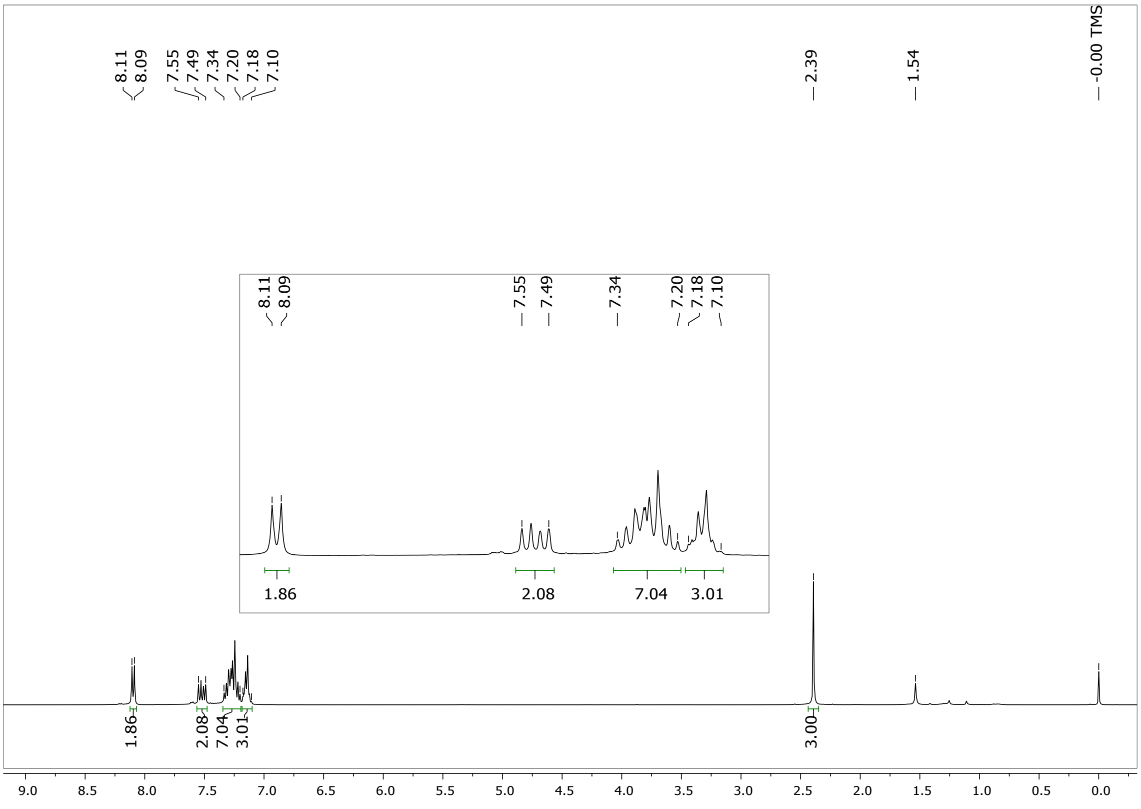

^{1}H NMR (400 MHz, $CDCl_3$) spectrum of compound **3i**.

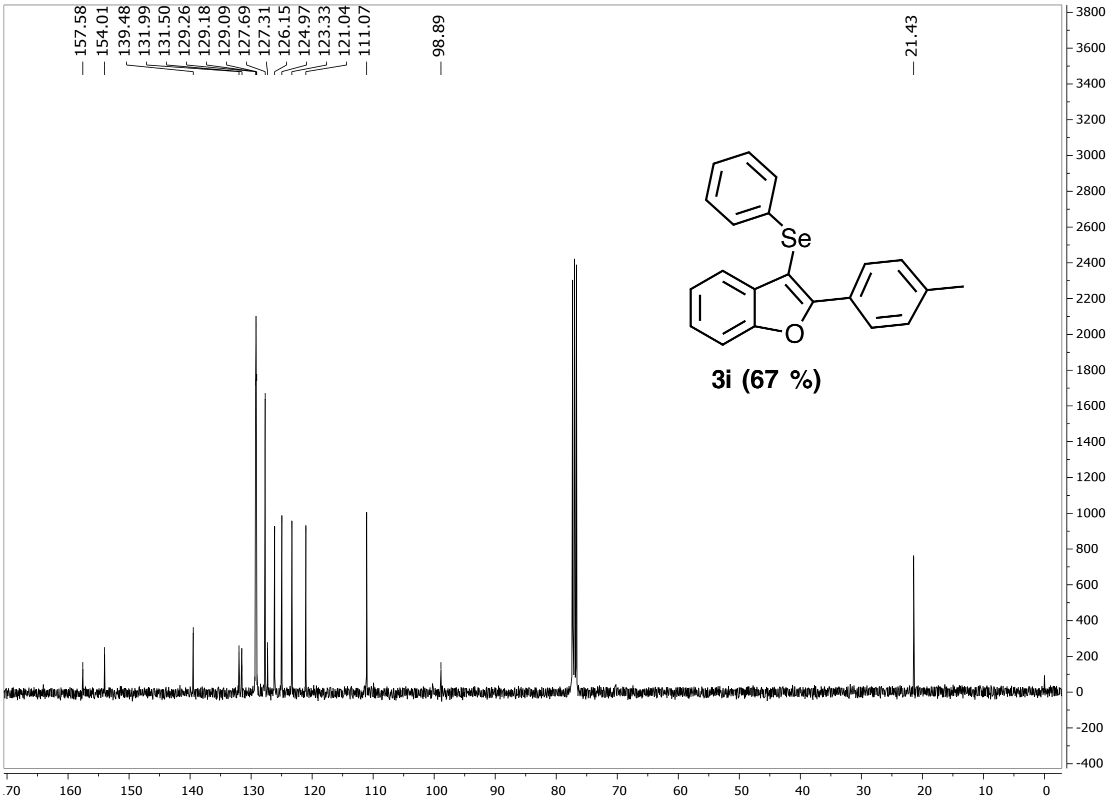

^{13}C NMR (100 MHz, $CDCl_3$) spectrum of compound **3i**.

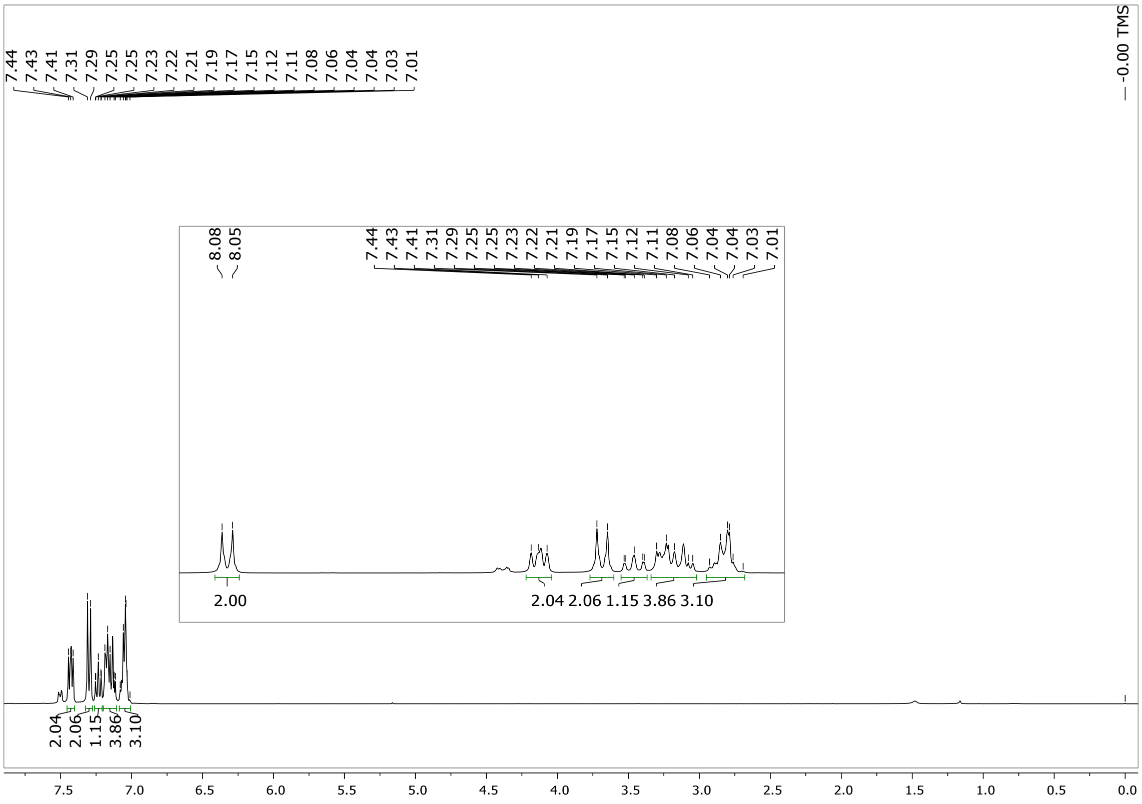

^{1}H NMR (400 MHz, $CDCl_3$) spectrum of compound **3j**.

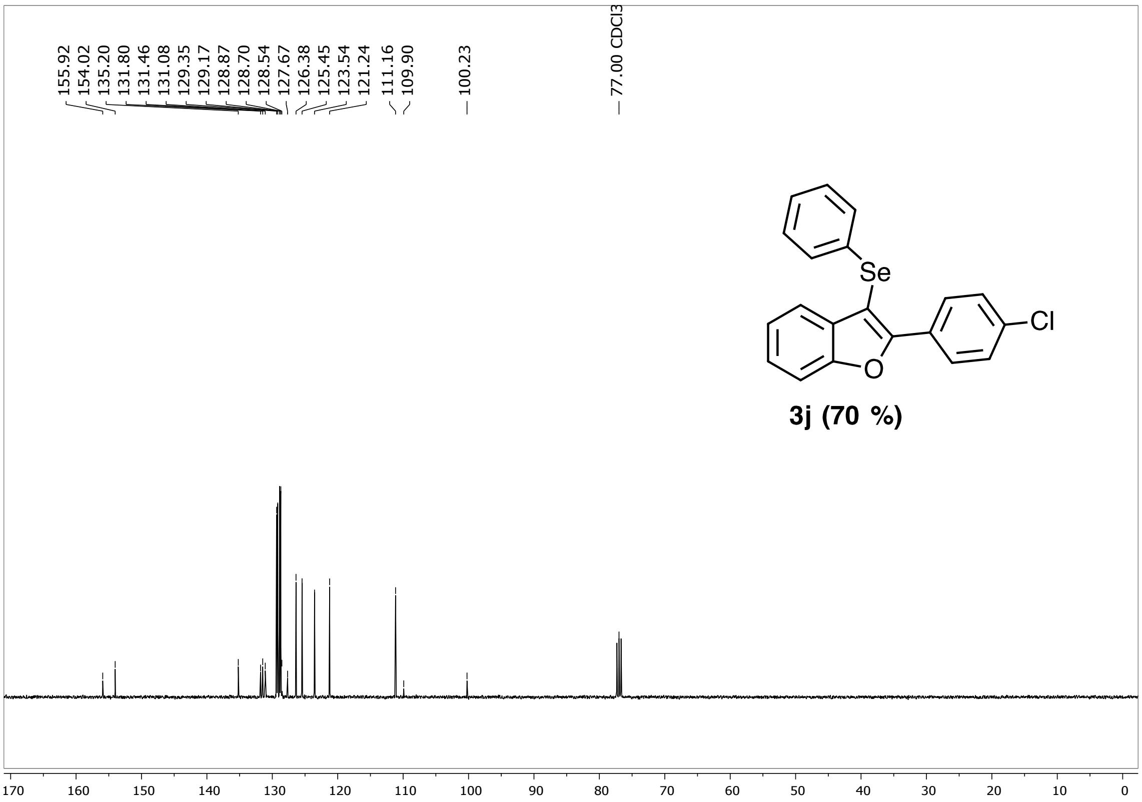

^{13}C NMR (100 MHz, $CDCl_3$) spectrum of compound **3j**.

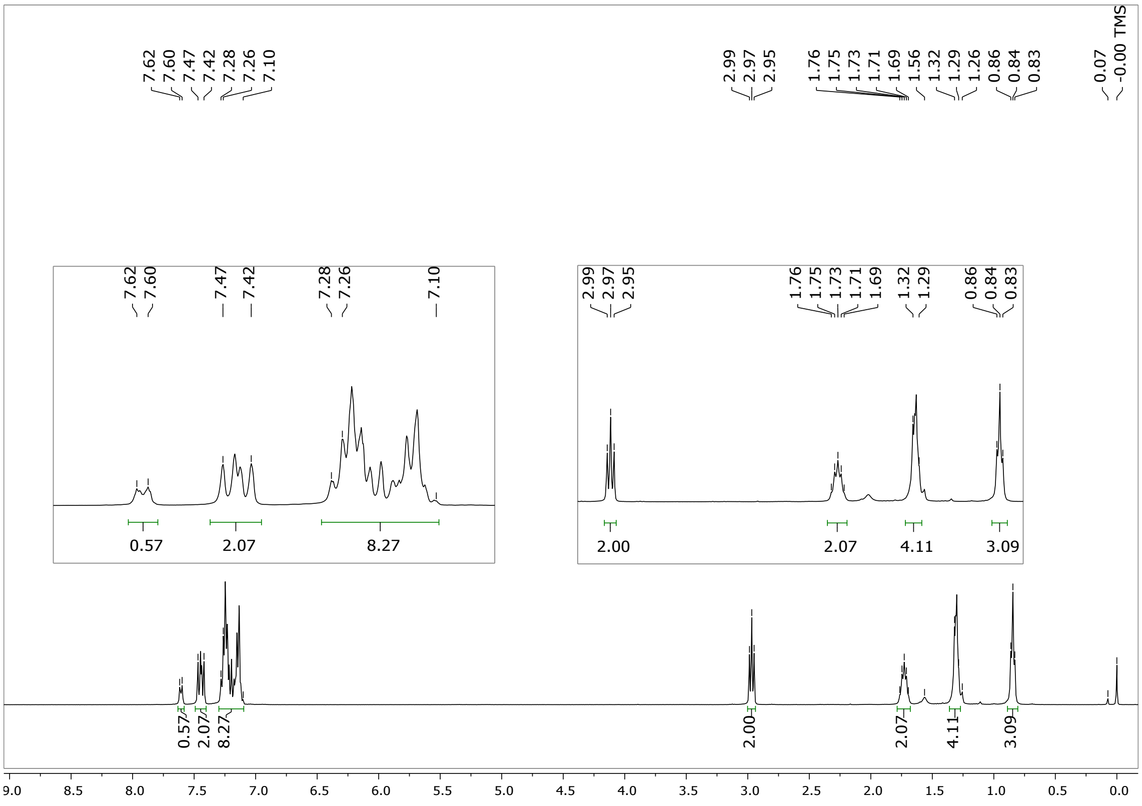

^{1}H NMR (400 MHz, $CDCl_3$) spectrum of compound **3k**.

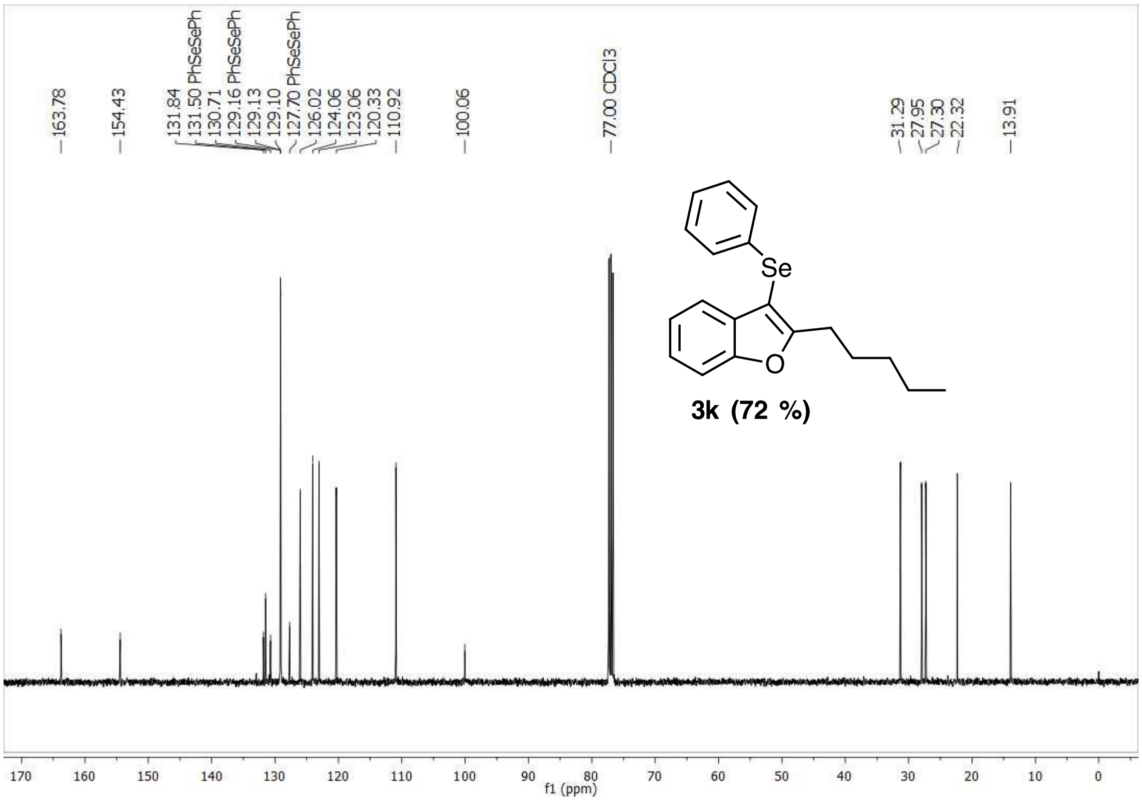

^{13}C NMR (100 MHz, $CDCl_3$) spectrum of compound **3k**.

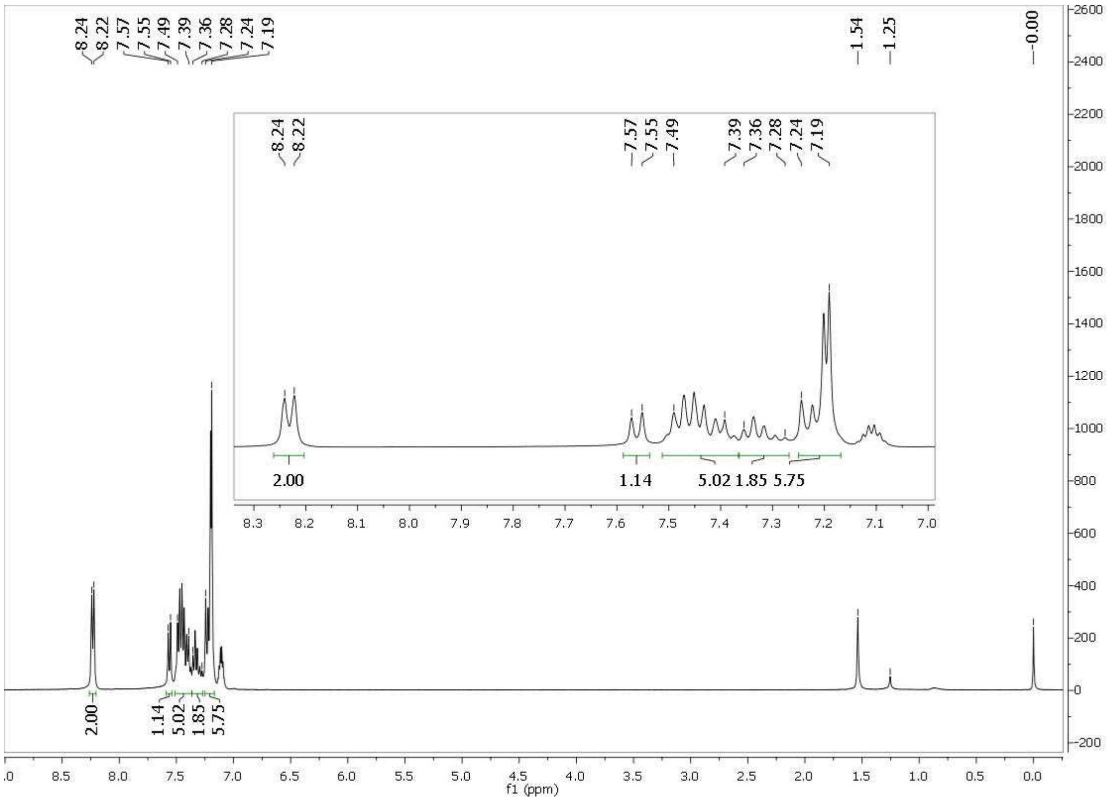

^{1}H NMR (400 MHz, $CDCl_3$) spectrum of compound **3l**.

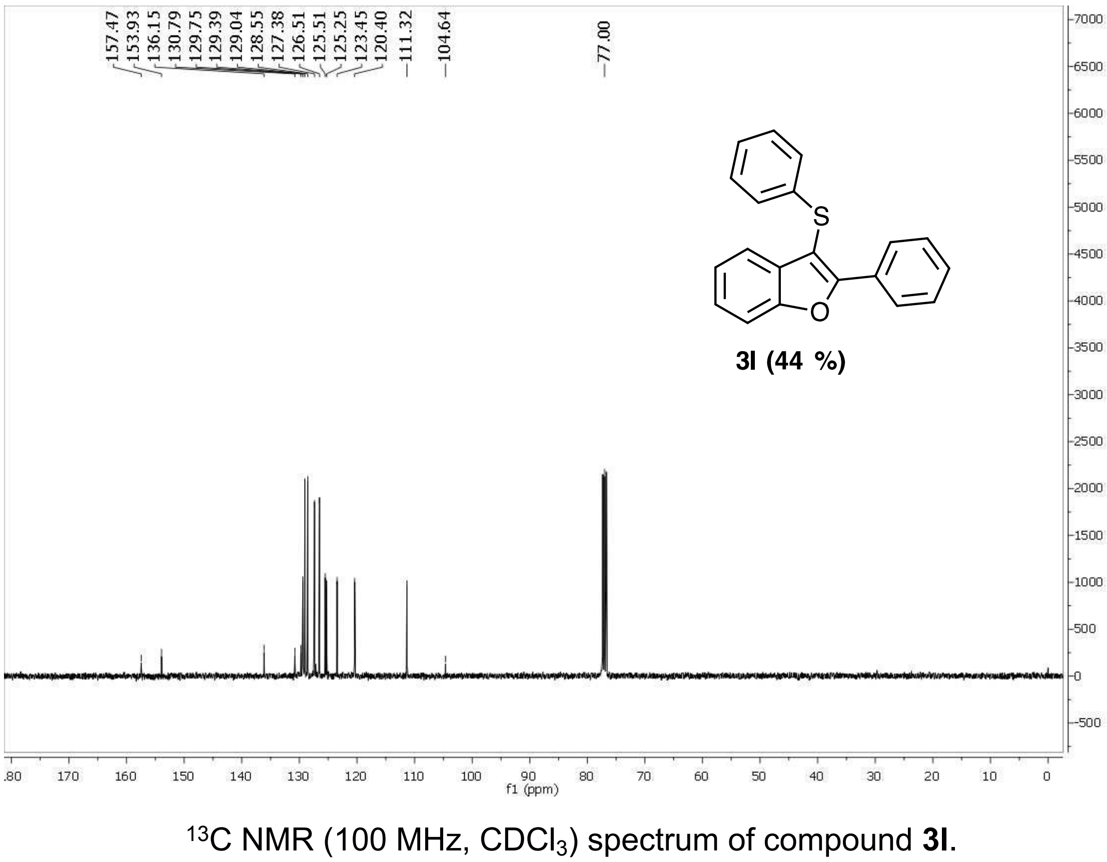

^{13}C NMR (100 MHz, $CDCl_3$) spectrum of compound **3l**.

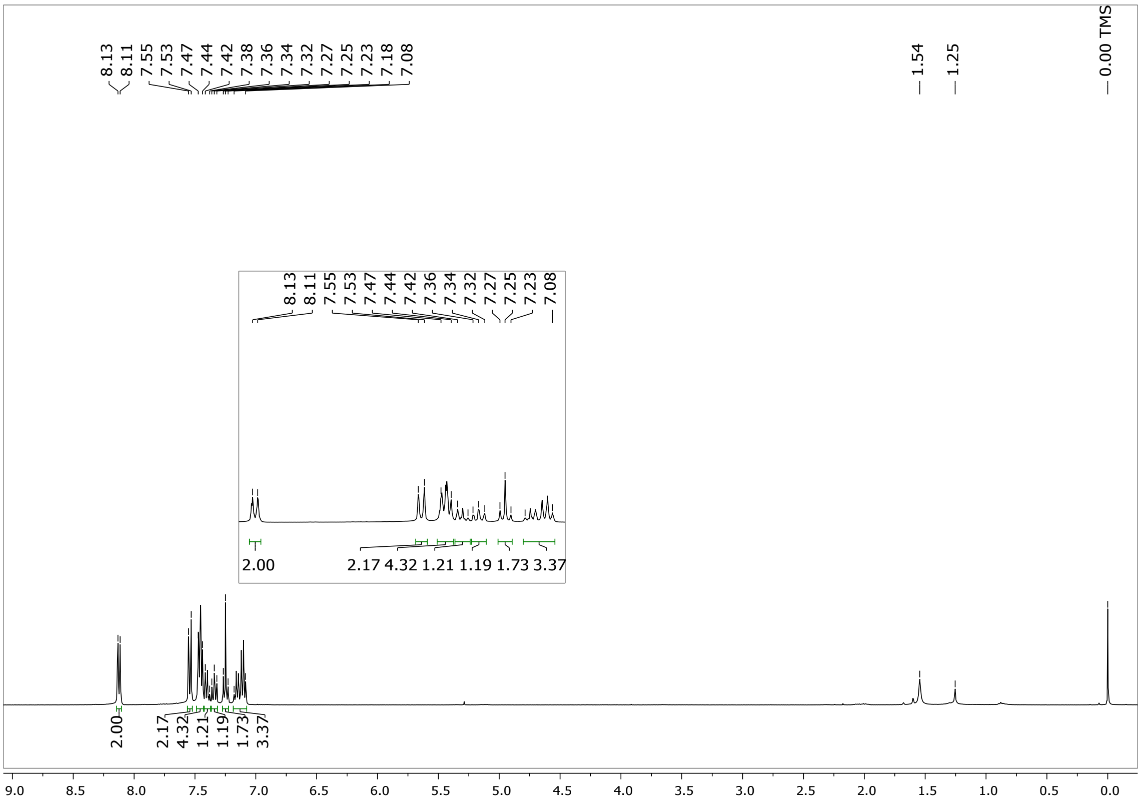

^{1}H NMR (400 MHz, $CDCl_3$) spectrum of compound **3m**.

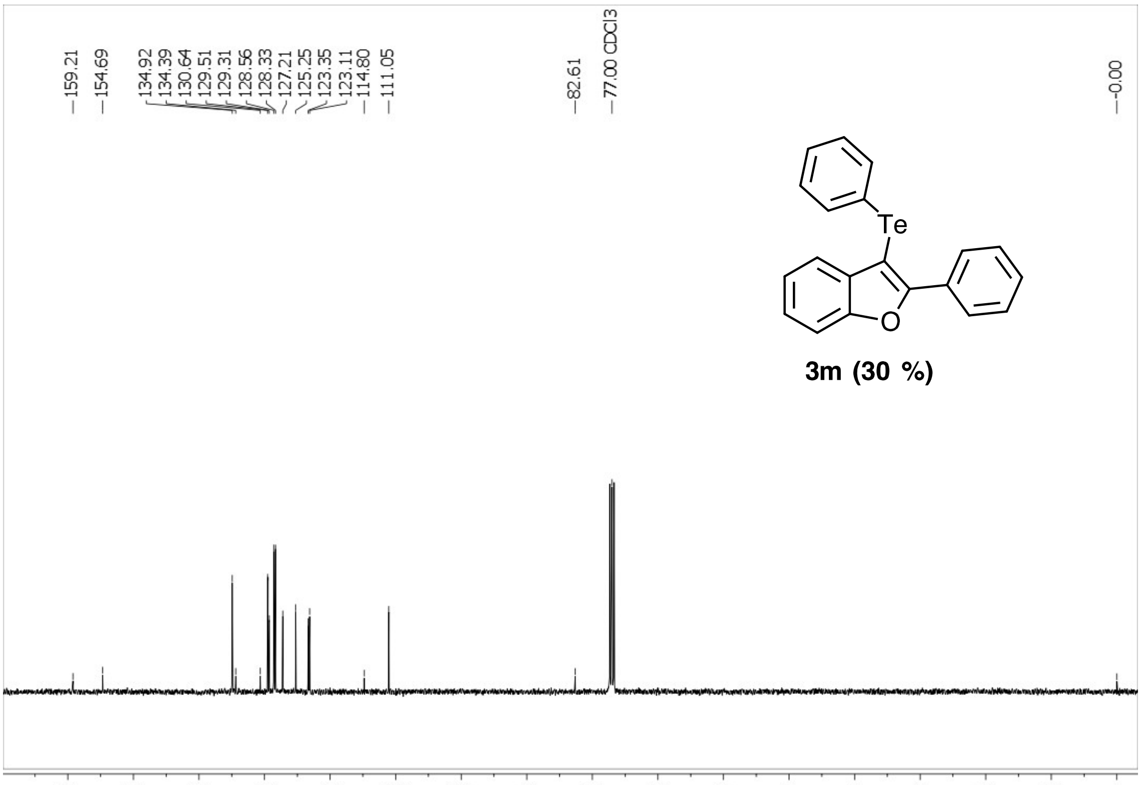

^{13}C NMR (100 MHz, $CDCl_3$) spectrum of compound **3m**.

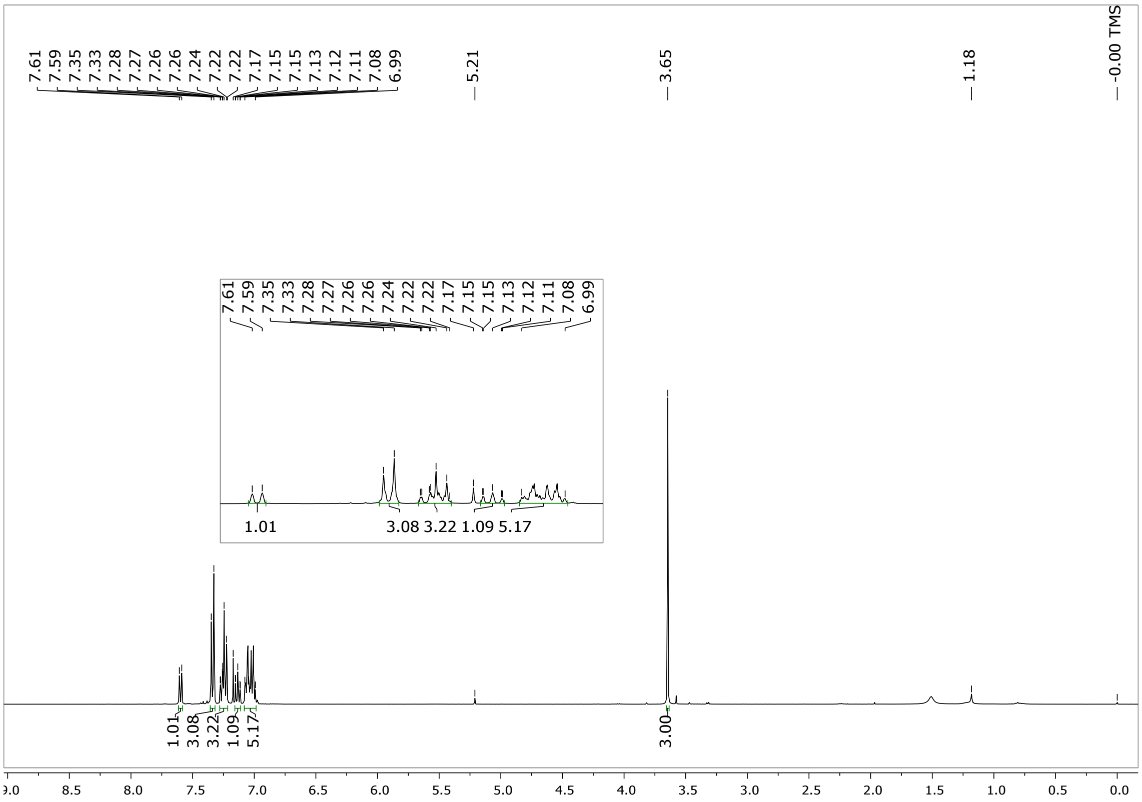

^1H NMR (400 MHz, $CDCl_3$) spectrum of compound **5b**.

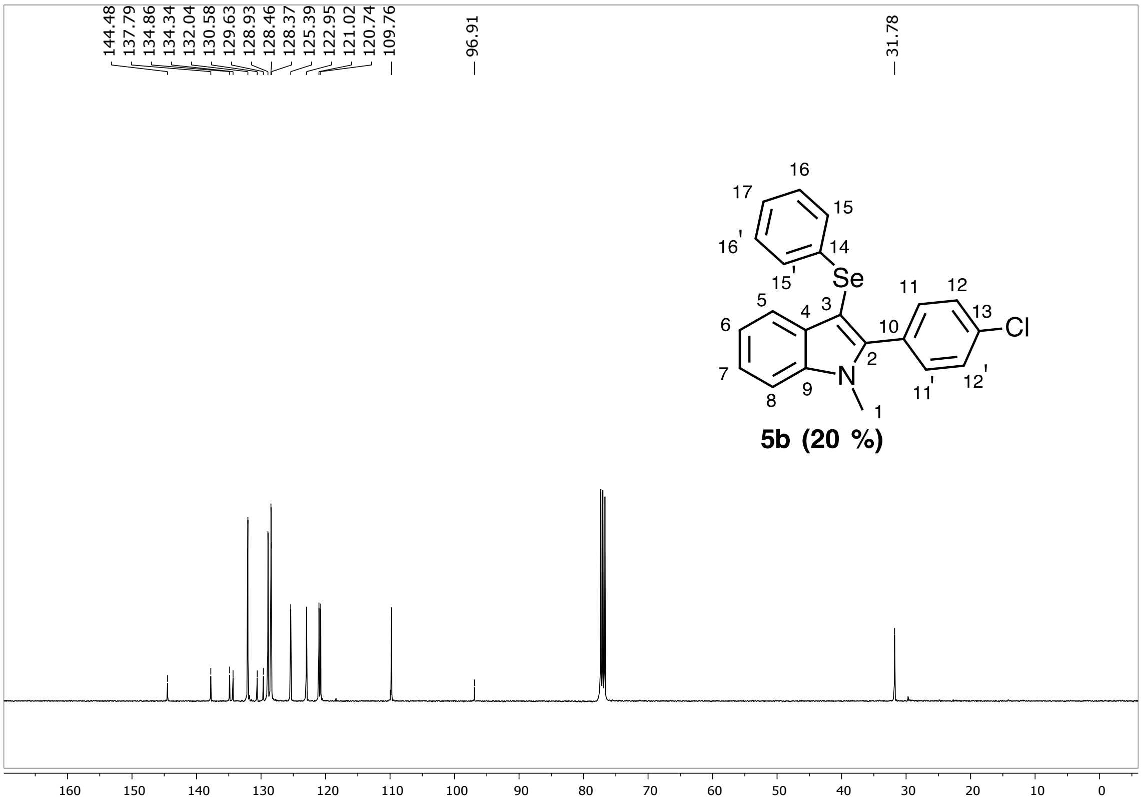

^{13}C NMR (100 MHz, $CDCl_3$) spectrum of compound **5b**.

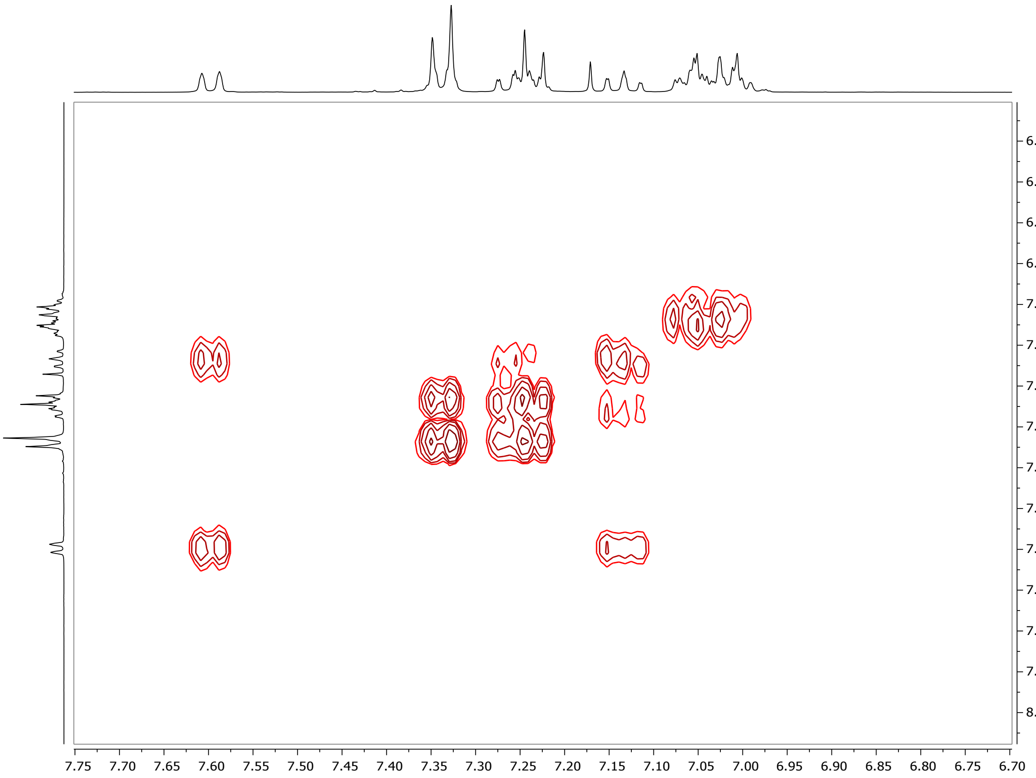

^{1}H – ^{1}H COSY (400 MHz, $CDCl_3$) spectrum of compound **5b**.

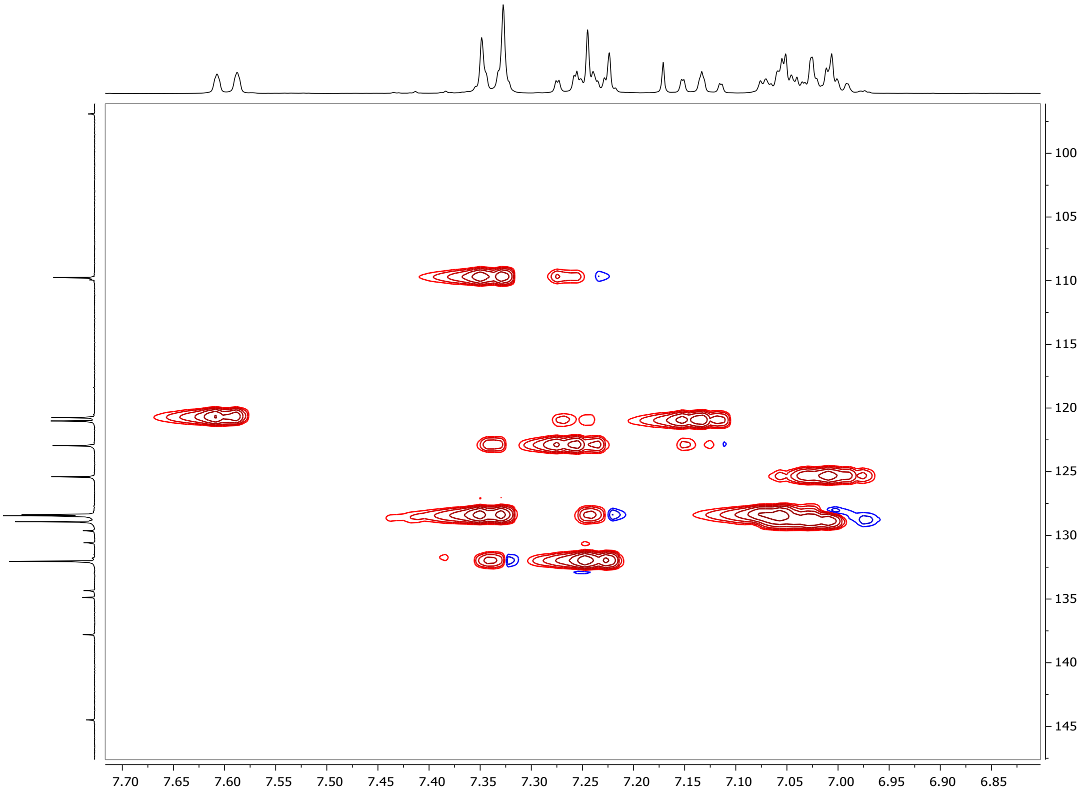

^{1}H – ^{13}C HSQC (400 MHz, $CDCl_3$) spectrum of compound **5b**.

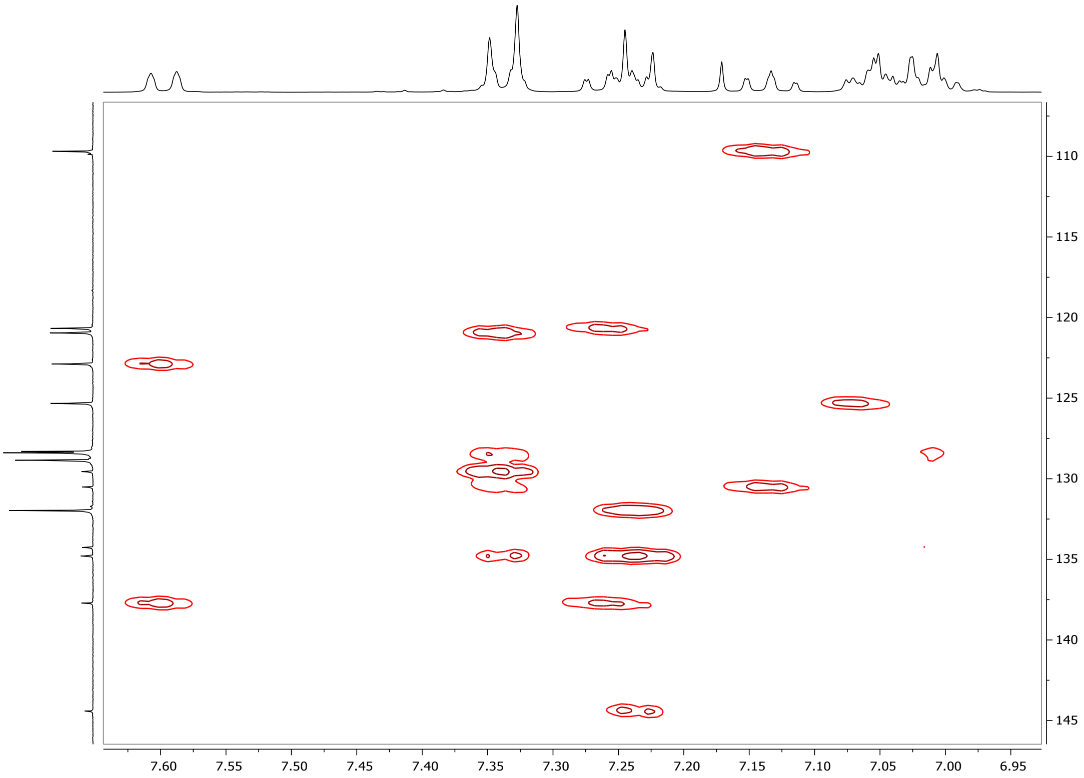

^{1}H – ^{13}C HMBC (400 MHz, $CDCl_3$) spectrum of compound **5b**.

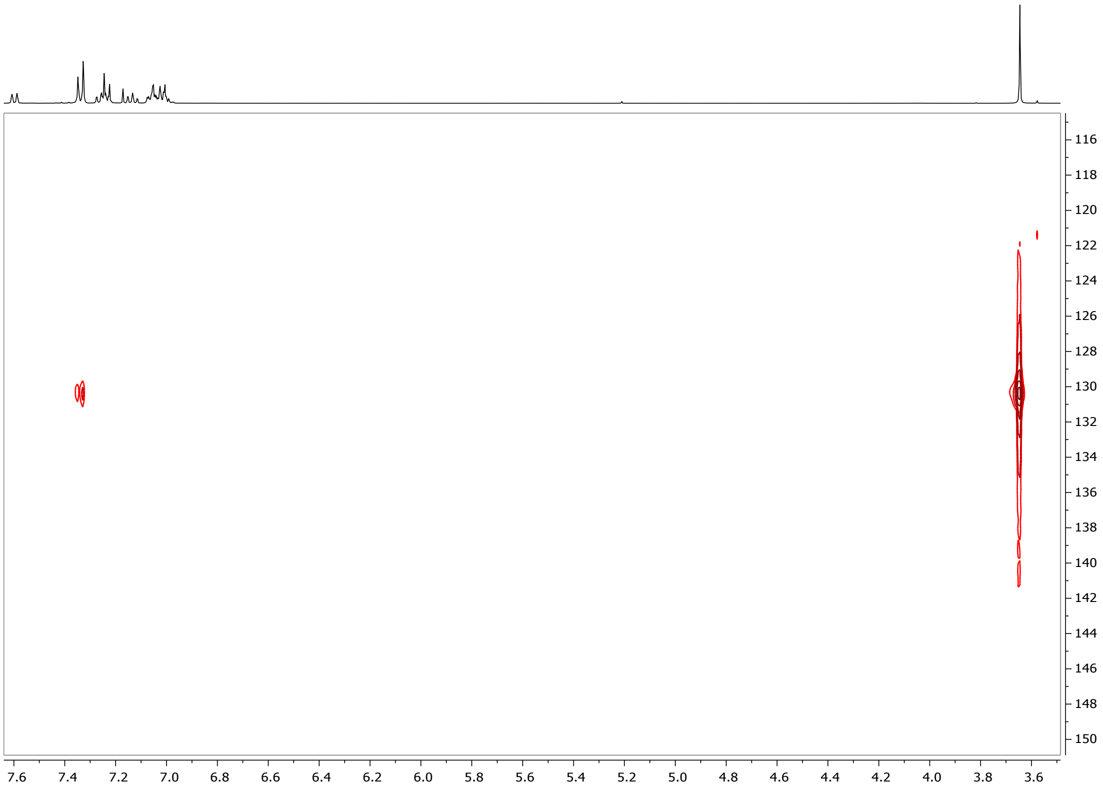

^{1}H – ^{15}N HMBC (400 MHz, $CDCl_3$) spectrum of compound **5b**.

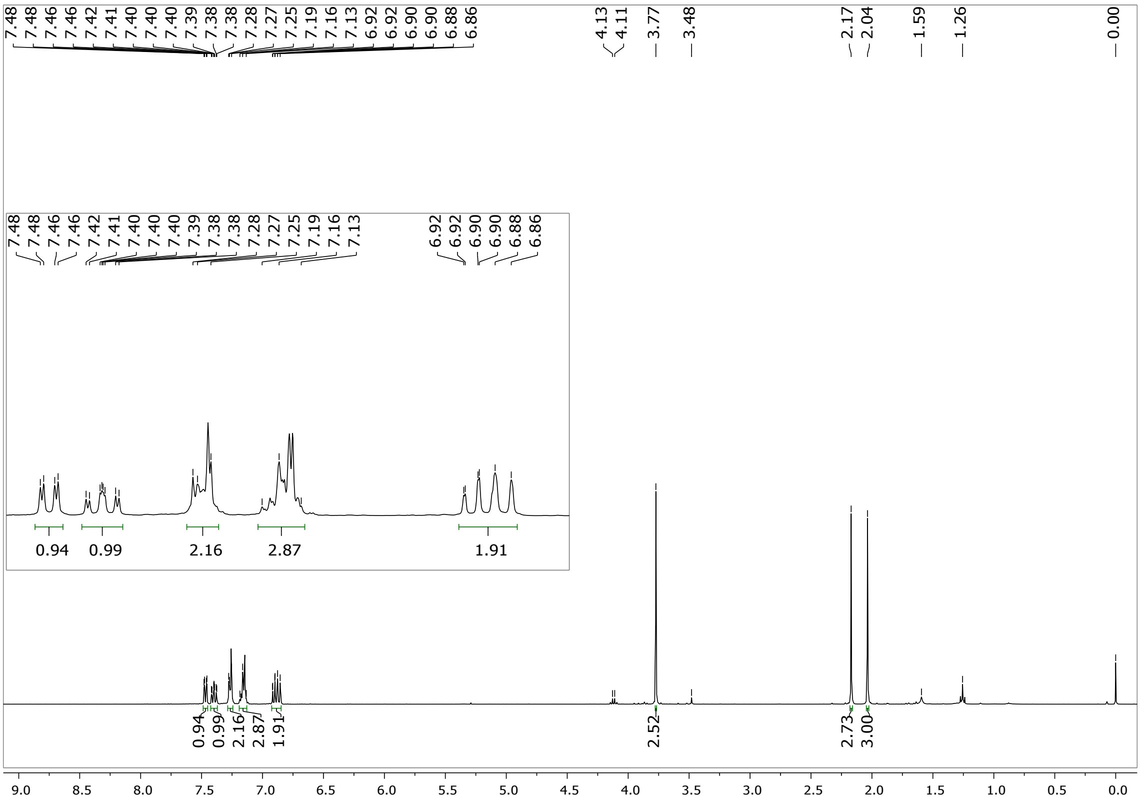

^{1}H NMR (400 MHz, $CDCl_3$) spectrum of compound **4a**.

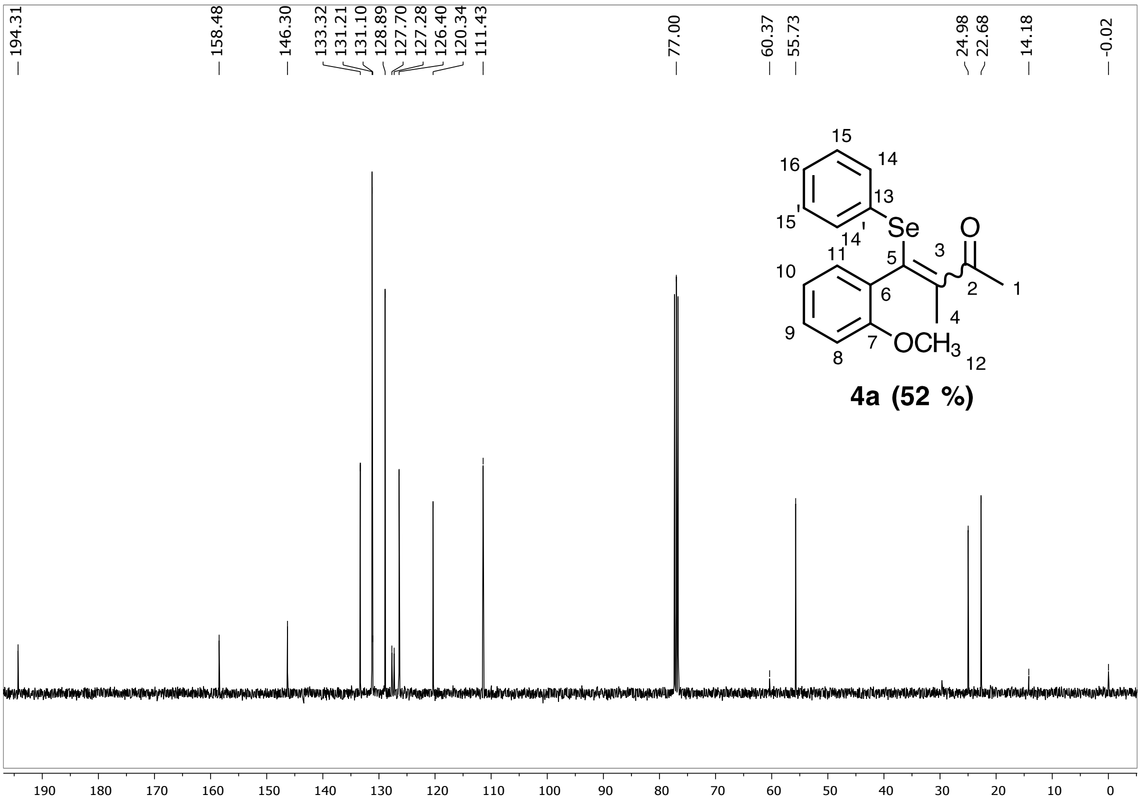

^{13}C NMR (100 MHz, $CDCl_3$) spectrum of compound **4a**.

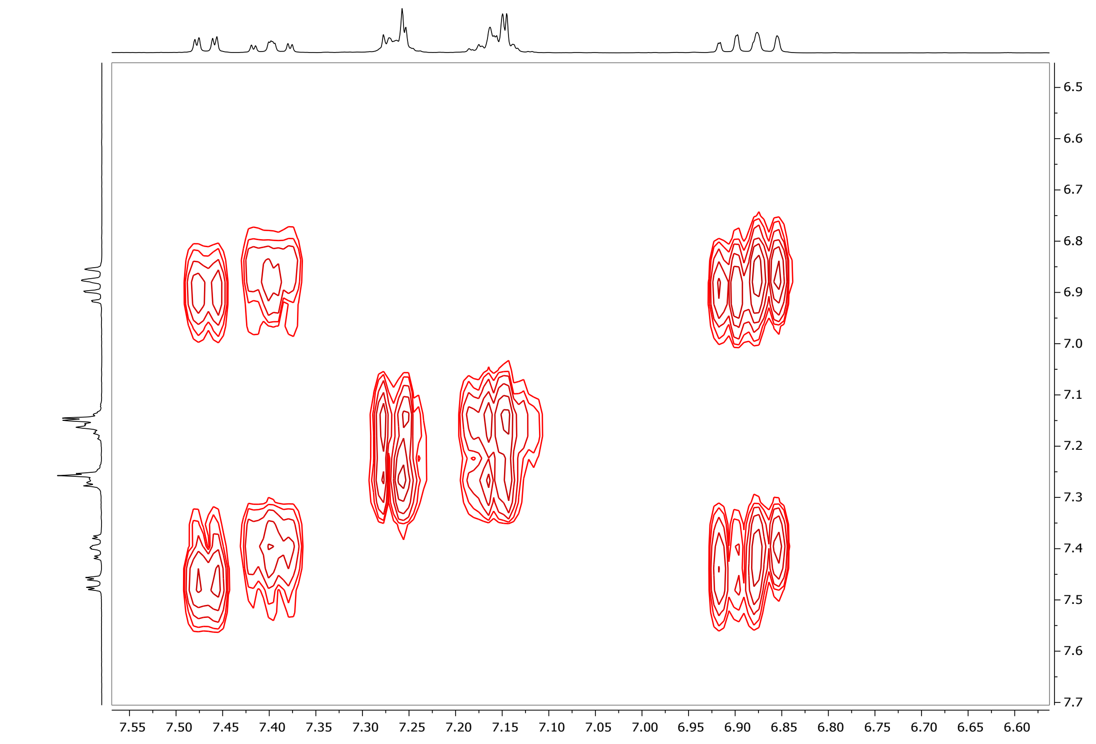

^{1}H – ^{1}H COSY (400 MHz, $CDCl_3$) spectrum of compound **4a**.

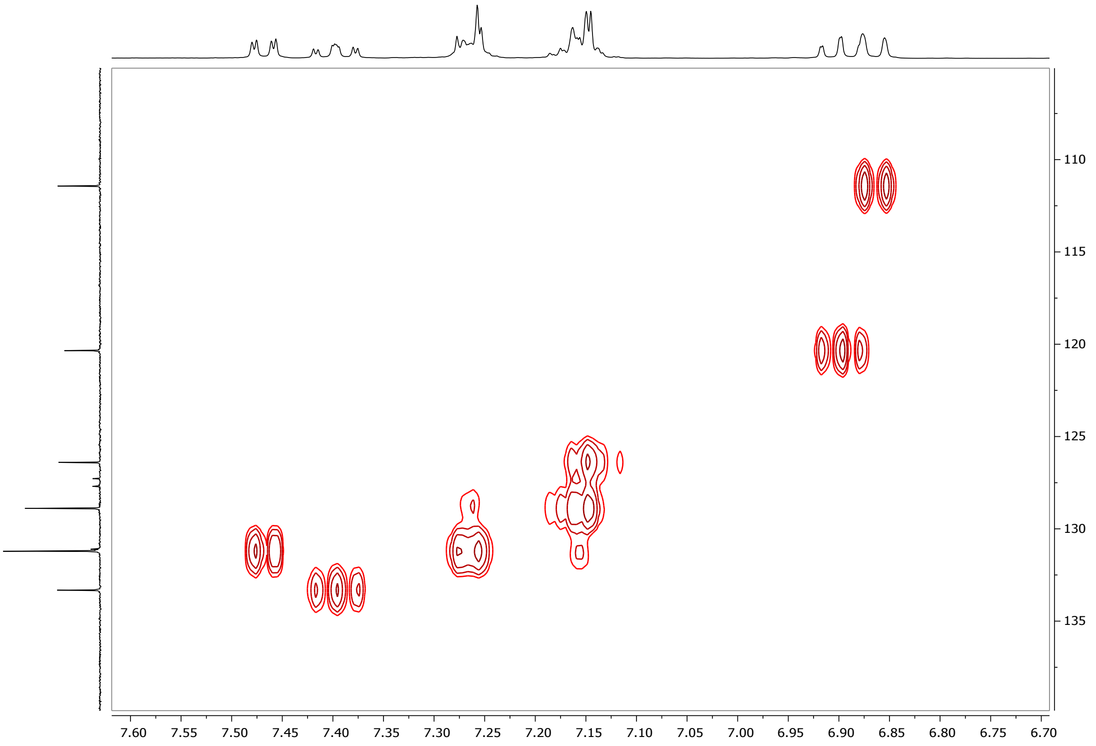

^{1}H – ^{13}C HSQC (400 MHz, $CDCl_3$) spectrum of compound **4a**.

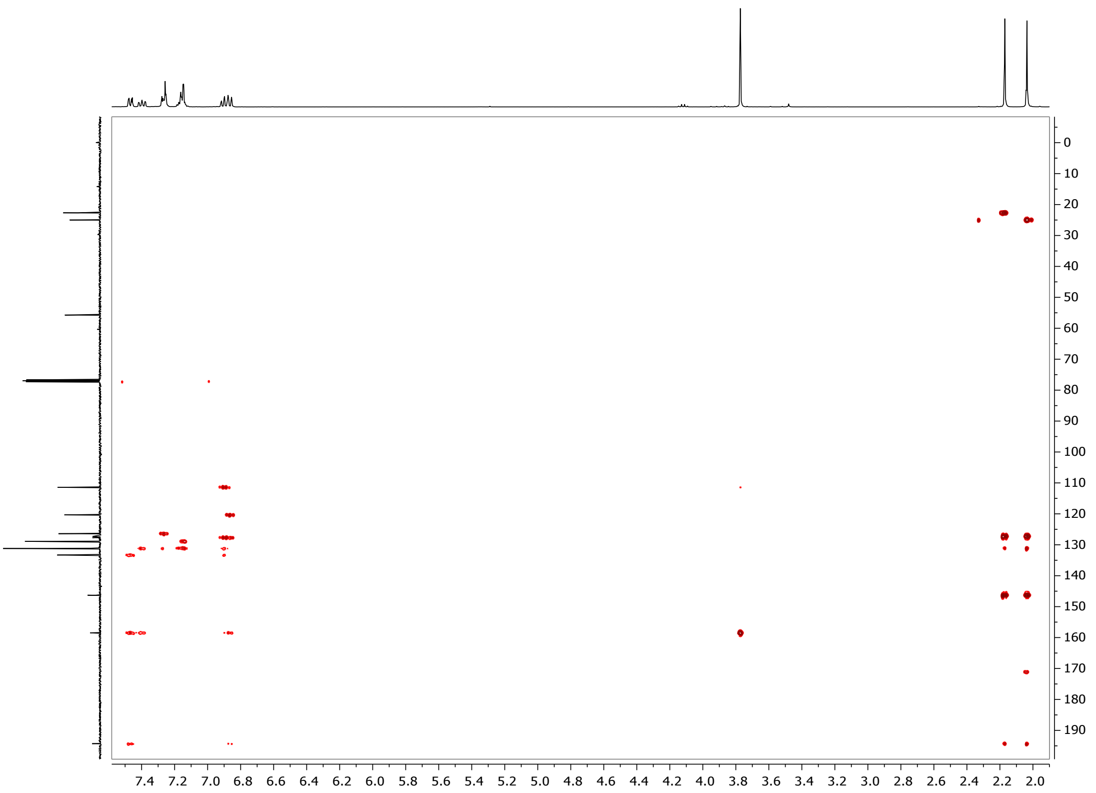

^{1}H – ^{13}C HMBC (400 MHz, $CDCl_3$) spectrum of compound **4a**.

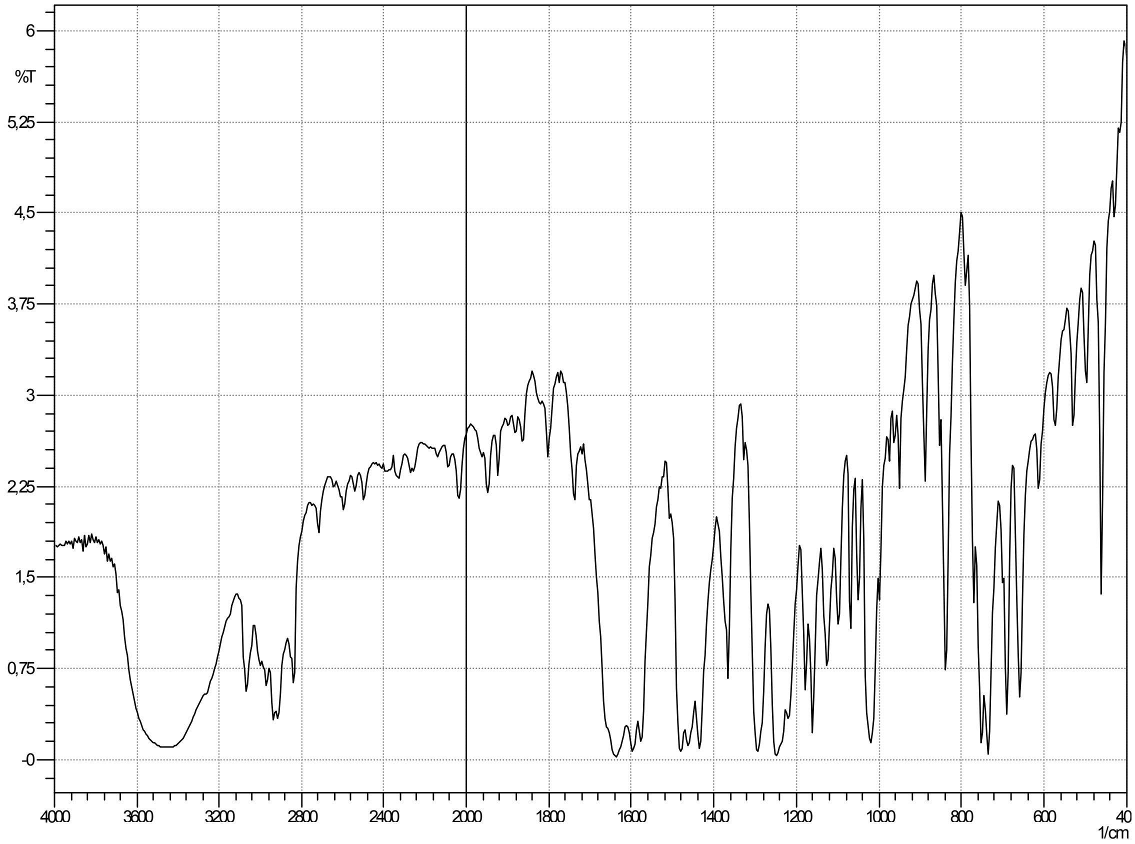

Infra-red spectrum of compound **4a**.

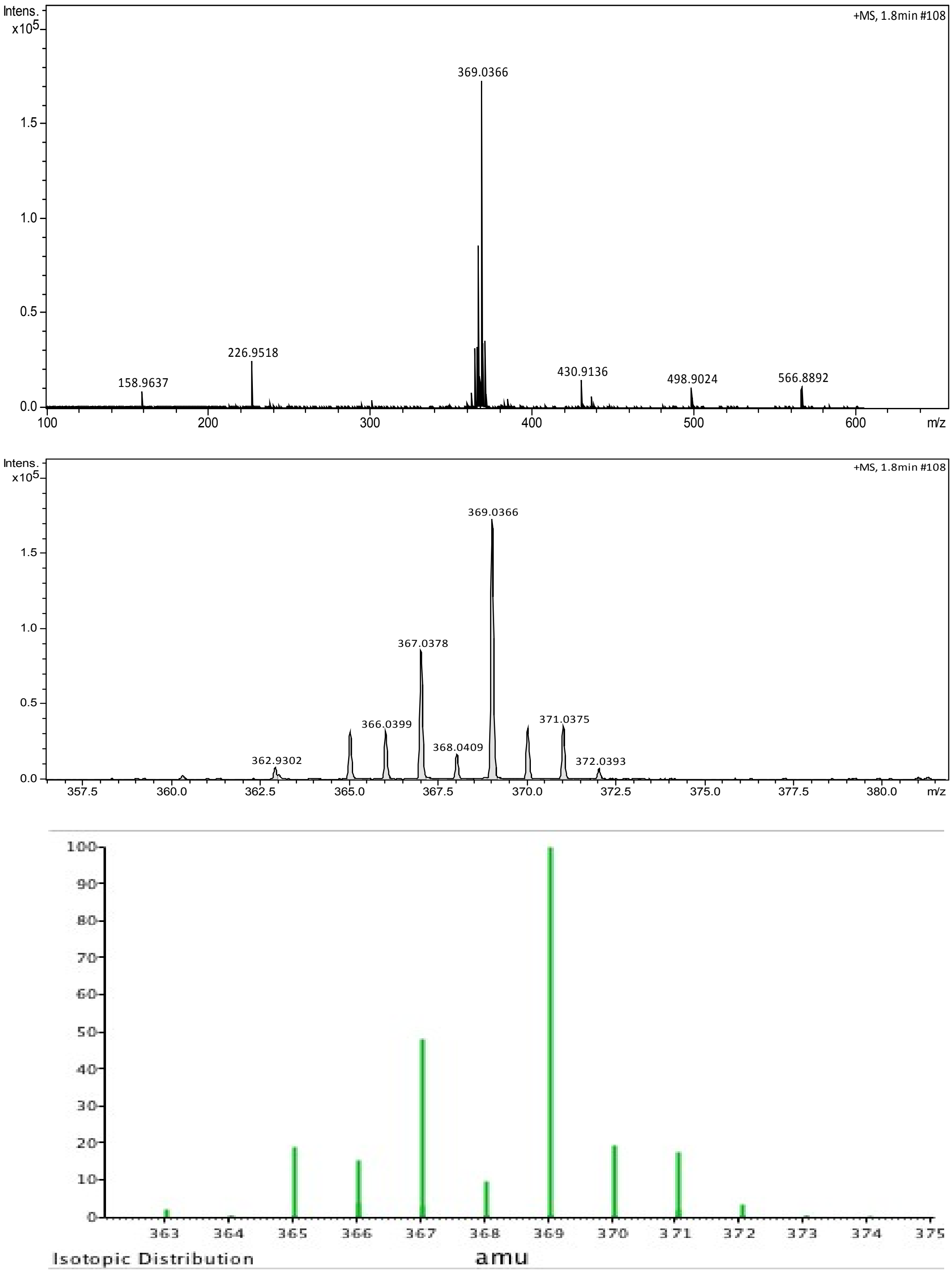

High resolution mass spectrometry of compound **4a** (MW + Na).

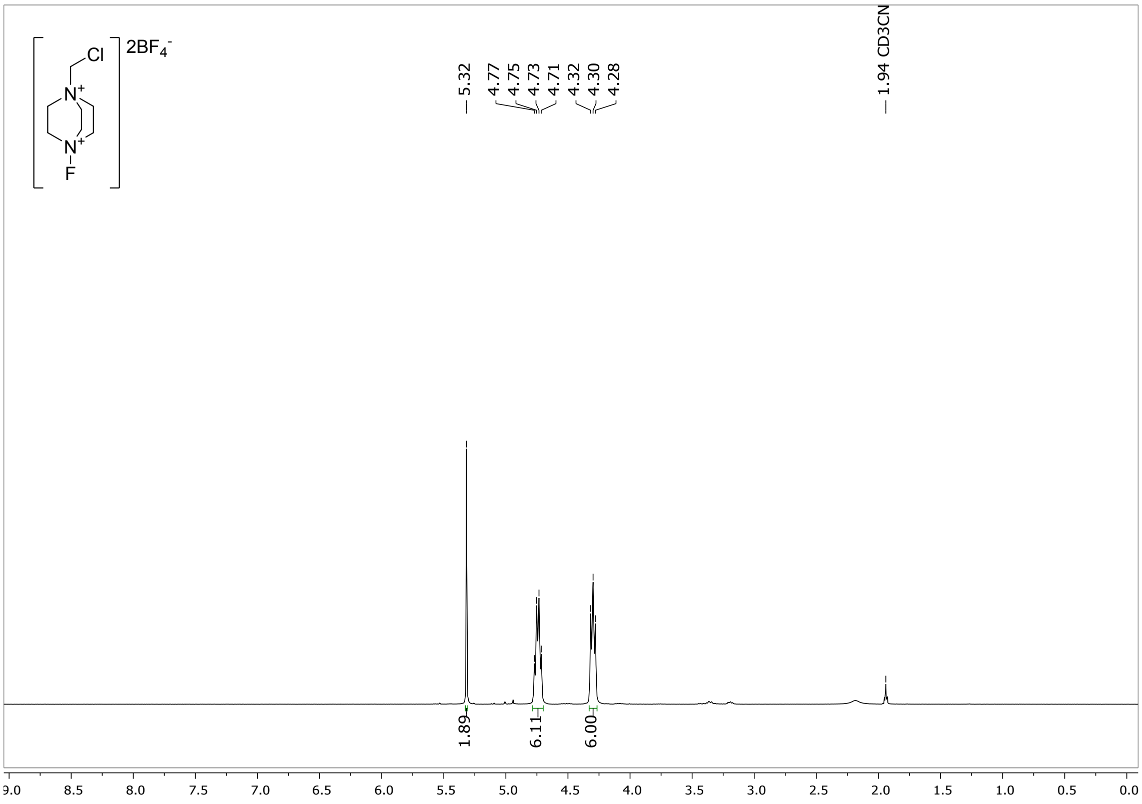

^{1}H NMR (CD_3CN, 400 MHz) spectrum of Selectfluor® compound.

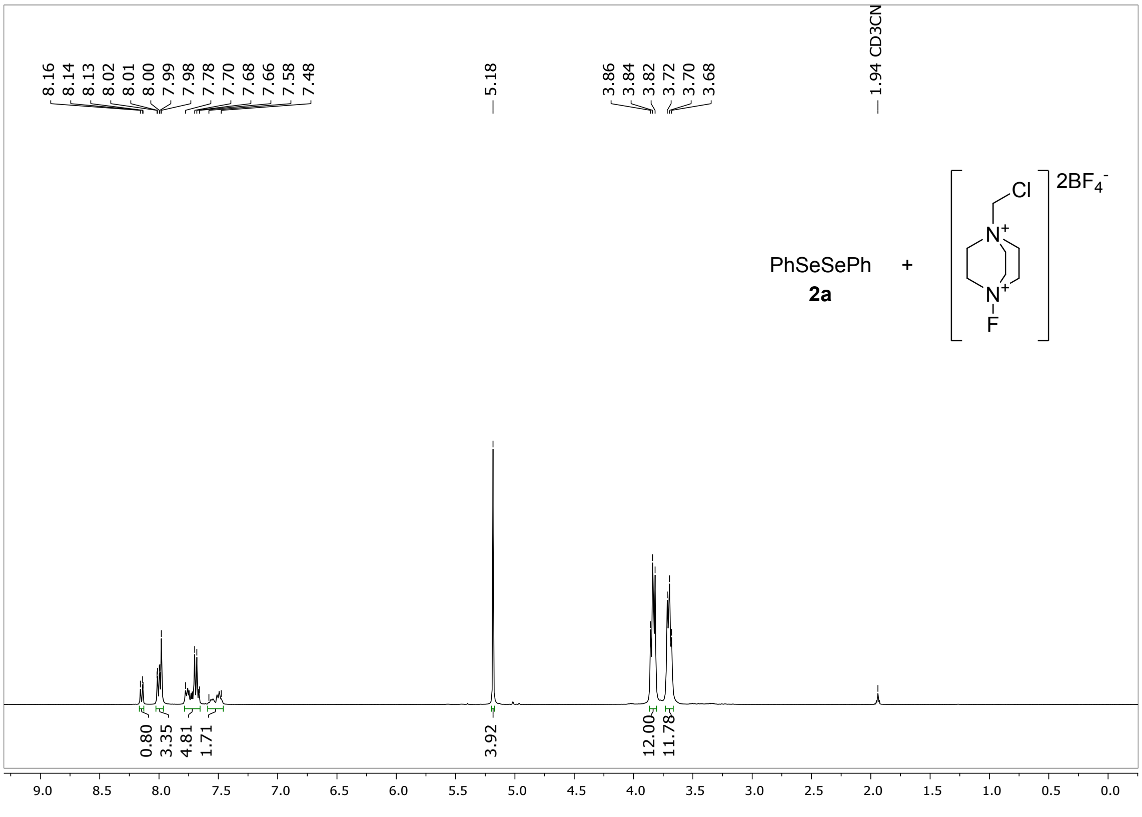

^{1}H NMR (CD_3CN, 400 MHz) spectrum of diphenyl diselenide and Selectfluor® mixture (1:2 ratio).

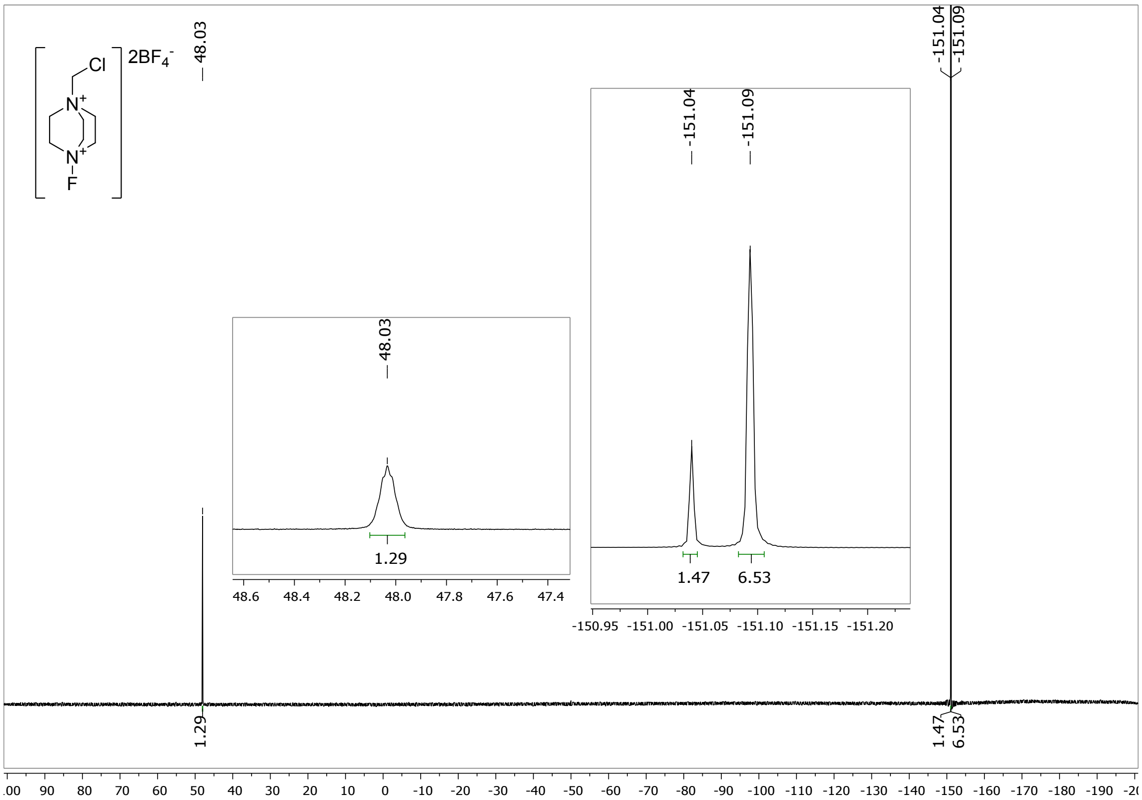

^{19}F NMR (CD_3CN, 376 MHz) spectrum of Selectfluor® compound.

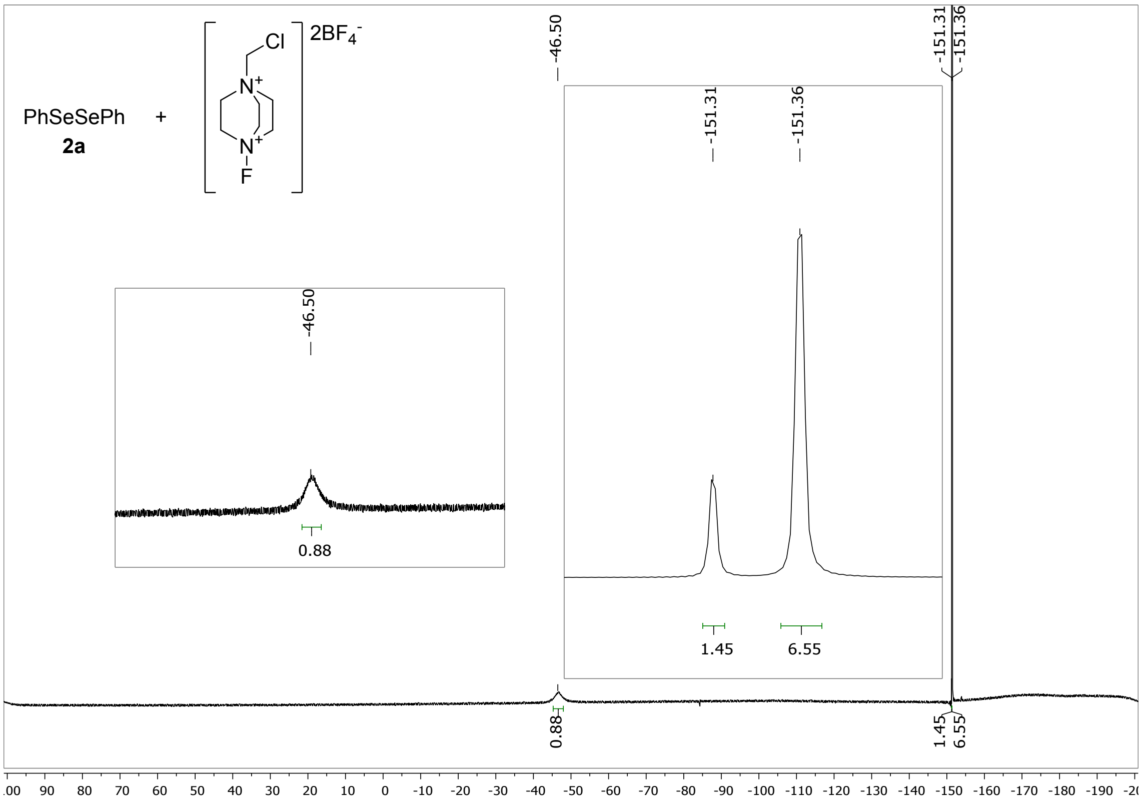

^{19}F NMR (CD_3CN, 376 MHz) spectrum of diphenyl diselenide and Selectfluor® mixture (1:1 ratio).

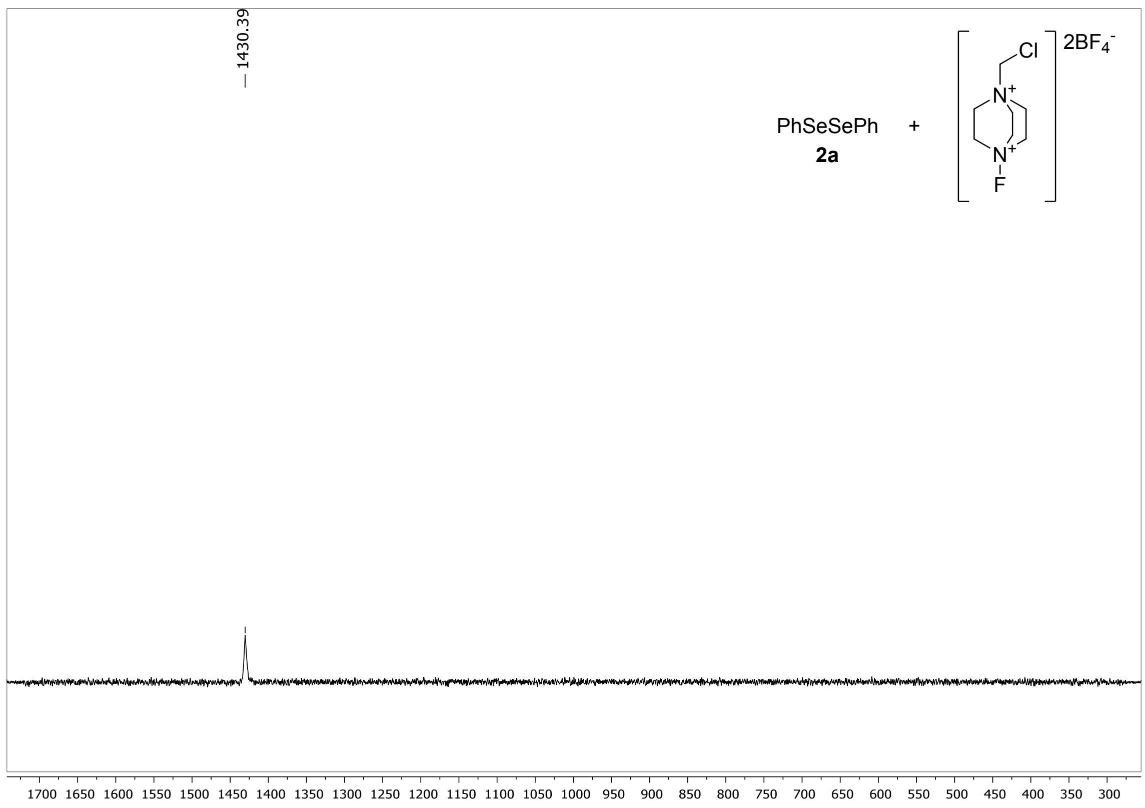

^{77}Se-{^{1}H} NMR (CD_3CN, 76 MHz) spectrum of diphenyl diselenide and Selectfluor® mixture (1:2 ratio).

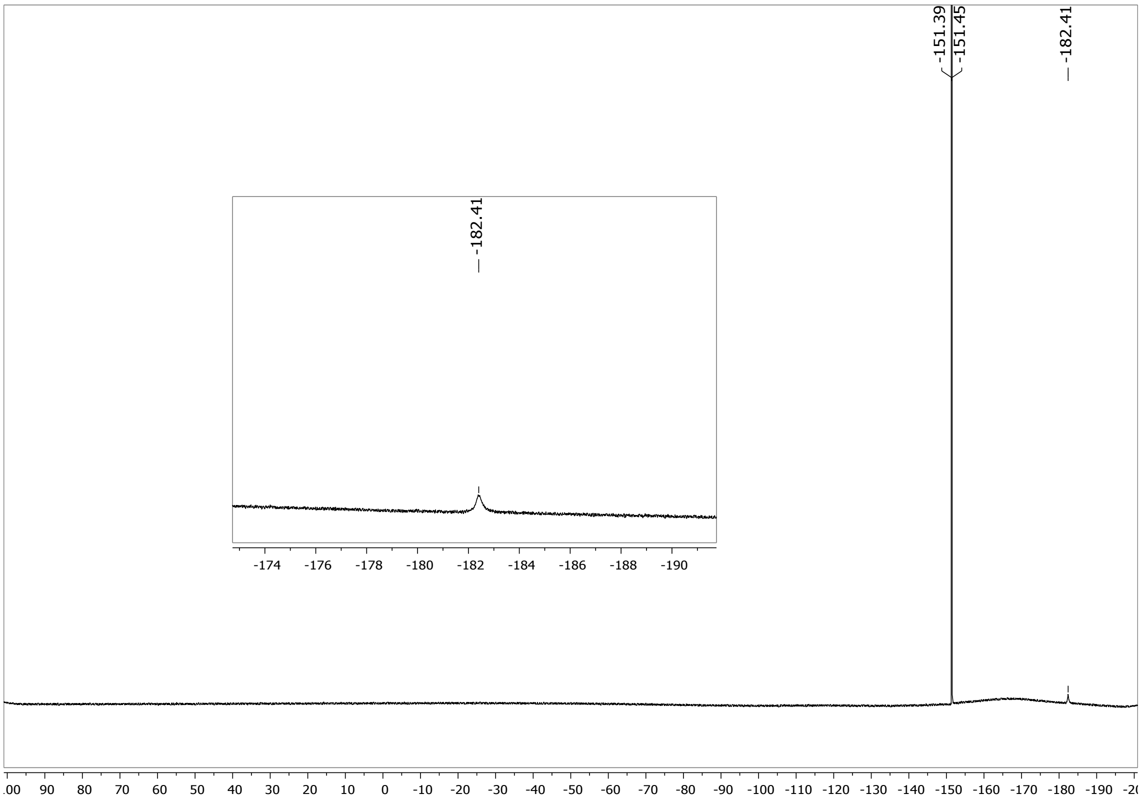

^{19}F NMR (CD_3CN, 376 MHz) spectrum of diphenyl diselenide and Selectfluor® mixture (1:2 ratio).
